# Author Correction: Type I IFNs promote cancer cell stemness by triggering the epigenetic regulator KDM1B

**DOI:** 10.1038/s41590-024-01984-w

**Published:** 2024-09-17

**Authors:** Martina Musella, Andrea Guarracino, Nicoletta Manduca, Claudia Galassi, Eliana Ruggiero, Alessia Potenza, Ester Maccafeo, Gwenola Manic, Luca Mattiello, Sara Soliman Abdel Rehim, Michele Signore, Marco Pietrosanto, Manuela Helmer-Citterich, Matteo Pallocca, Maurizio Fanciulli, Tiziana Bruno, Francesca De Nicola, Giacomo Corleone, Anna Di Benedetto, Cristiana Ercolani, Edoardo Pescarmona, Laura Pizzuti, Francesco Guidi, Francesca Sperati, Sara Vitale, Daniele Macchia, Massimo Spada, Giovanna Schiavoni, Fabrizio Mattei, Adele De Ninno, Luca Businaro, Valeria Lucarini, Laura Bracci, Eleonora Aricò, Giovanna Ziccheddu, Francesco Facchiano, Stefania Rossi, Massimo Sanchez, Alessandra Boe, Mauro Biffoni, Ruggero De Maria, Ilio Vitale, Antonella Sistigu

**Affiliations:** 1 Dipartimento di Medicina e Chirurgia Traslazionale, Università Cattolica del Sacro Cuore, Rome, Italy; 2 Department of Biology, University of Rome ‘Tor Vergata’, Rome, Italy; 3 Genomics Research Centre, Human Technopole, Milan, Italy; 4 Department of Radiation Oncology, Weill Cornell Medical College, New York, NY USA; 5 Experimental Hematology Unit, IRCCS San Raffaele Scientific Institute, Milan, Italy; 6 Italian Institute for Genomic Medicine (IIGM), Candiolo, Italy; 7 Candiolo Cancer Institute, FPO - IRCCS, Candiolo, Italy; 8 RPPA Unit, Proteomics Area, Core Facilities, Istituto Superiore di Sanità, Rome, Italy; 9 UOSD Clinical Trial Center, Biostatistics and Bioinformatics, IRCCS Regina Elena National Cancer Institute, Rome, Italy; 10 SAFU Unit, IRCCS Regina Elena National Cancer Institute, Rome, Italy; 11 Pathology Unit, IRCCS Regina Elena National Cancer Institute, Rome, Italy; 12 Division of Medical Oncology 2, IRCCS Regina Elena National Cancer Institute, Rome, Italy; 13 Fondazione Policlinico Universitario ‘A. Gemelli’ - IRCCS, Rome, Italy; 14 UOSD Clinical Trial Center, Biostatistics and Bioinformatics, IRCCS San Gallicano Dermatological Institute, Rome, Italy; 15 Center of Animal Research and Welfare, Istituto Superiore di Sanità, Rome, Italy; 16 Department of Oncology and Molecular Medicine, Istituto Superiore di Sanità, Rome, Italy; 17 Institute for Photonics and Nanotechnologies, Italian National Research Council, Rome, Italy; 18 Department of Paediatric Haematology/Oncology and of Cell and Gene Therapy, Ospedale Pediatrico Bambino Gesù, IRCCS, Rome, Italy; 19 FaBioCell, Core Facilities, Istituto Superiore di Sanità, Rome, Italy; 20 Oncogenomics and Epigenetics, IRCCS Regina Elena National Cancer Institute, Rome, Italy; 21 Cytometry Unit, Core Facilities, Istituto Superiore di Sanità, Rome, Italy; 22 Tumor Immunology and Immunotherapy Unit, IRCCS Regina Elena National Cancer Institute, Rome, Italy

**Keywords:** Immunoediting, Cancer immunotherapy, Immunoediting

Correction to: *Nature Immunology* 10.1038/s41590-022-01290-3, published online 24 August 2022.

In the version of this article initially published, due to an error during figure assembly, the image shown in the top-right WT mice, PAR cells, panel of Fig. 4d was a duplicate of the lower-left image in the CD4^–/–^CD8^–/–^ mice, CD44L cells, panel. The figure has been updated in the HTML and PDF versions of the article.

