# Supplementary material for: Type I IFNs promote cancer cell stemness by triggering the epigenetic regulator KDM1B
Source: Nat Immunol. 2022 Aug 24;23(9):1379–92. doi: 10.1038/s41590-022-01290-3 (PMC9477743; doi:10.1038/s41590-022-01290-3)
Supplement: Supplementary file 1 — Supplementary legends, Figs. 1–4 and Tables 1–6. [file 41590_2022_1290_MOESM1_ESM.pdf]

nature immunology
SUPPLEMENTARY INFORMATION
https://doi.org/10.1038/s41590-022-01290-3
In the format provided by the authors and unedited.

Type I IFNs promote cancer cell stemness by triggering the epigenetic regulator KDM1B

In the format provided by the authors and unedited

SUPPLEMENTARY FIGURE LEGENDS

Supplementary Figure 1. Gating strategy for flow cytometry. (**a,b**) Representative gating strategies for *in vitro* cancer cell analysis (**a**) and *ex vivo* tumor infiltrating lymphocyte (TIL) analysis (**b**). During flow cytometer acquisition and data analysis, samples were sequentially gated (1) for morphology, by plotting forward scatter area (FSC-A) and side scatter area (SSC-A); (2) for singlets, by plotting FSC-A and forward scatter height (FSC-H) or SSC-A and side scatter width (SSC-W); and (3) for viability, by plotting DAPI and SSC-A or FSC-H. Viable cells were finally analysed for specific surface markers [*e.g.*, CD133 within $CD24^{+}CD44^{+}$ cells (**a**) or TIM3 within CD8+ cells (**b**)].

Supplementary Figure 2. Multiparametric flow cytometry analysis of cancer stem cell (CSC) surface markers in MCA205 *Ifih*$^{-/-}$ cell clones treated with oxaliplatin (OXP, 300 µM 24h) alone or in combination with the AIM2 inhibitor thalidomide (AIM2 inh, 10 µg/mL) or inhibitors or the RIG-I pathway amlexanox (RIG-I inh#1, 5 µM), BX795 (RIG-I inh#2, 100 nM) and MRT67307 (RIG-I inh#3, 500 nM) as indicated. The histograms represent the percentage (mean±SEM and individual data points, the number of independent experiments each with 3 technical replicates is shown) of $CD133^{+}CD24^{+}CD44^{+high}$ (CD44H) and $CD133^{+}CD24^{+}CD44^{+low}$ (CD44L) cells. Ordinary one-way ANOVA test followed by Bonferroni's correction.

Supplementary Figure 3. Chromatin remodeling following type I interferon (IFN-I) exposure. Transcriptional factor (TF) motifs enriched at least 2-fold for each locus as identified by ATAC-seq are shown in **a**, while a list of selected genes upregulated (red) and downregulated (blue) in CD44H IFN-CSCs and used to generate a gene ontology (GO) network analysis is reported in **b**. (**a**) One-sided binomial test followed by Benjamini-Hochberg correction for multiple comparisons. * adjusted $P \leq 0.001$. ATAC-seq, assay for transposase-accessible chromatin using sequencing; CD44H, $CD133^{+}CD24^{+}CD44^{+high}$.

Supplementary Figure 4. Evaluation of gene regulatory mechanisms downstream of KDM1B by Chip-seq on immunogenic cell death (ICD)-induced CD44H cells isolated from MCA205 cells and Gene Ontology (GO) terms enrichment analysis.

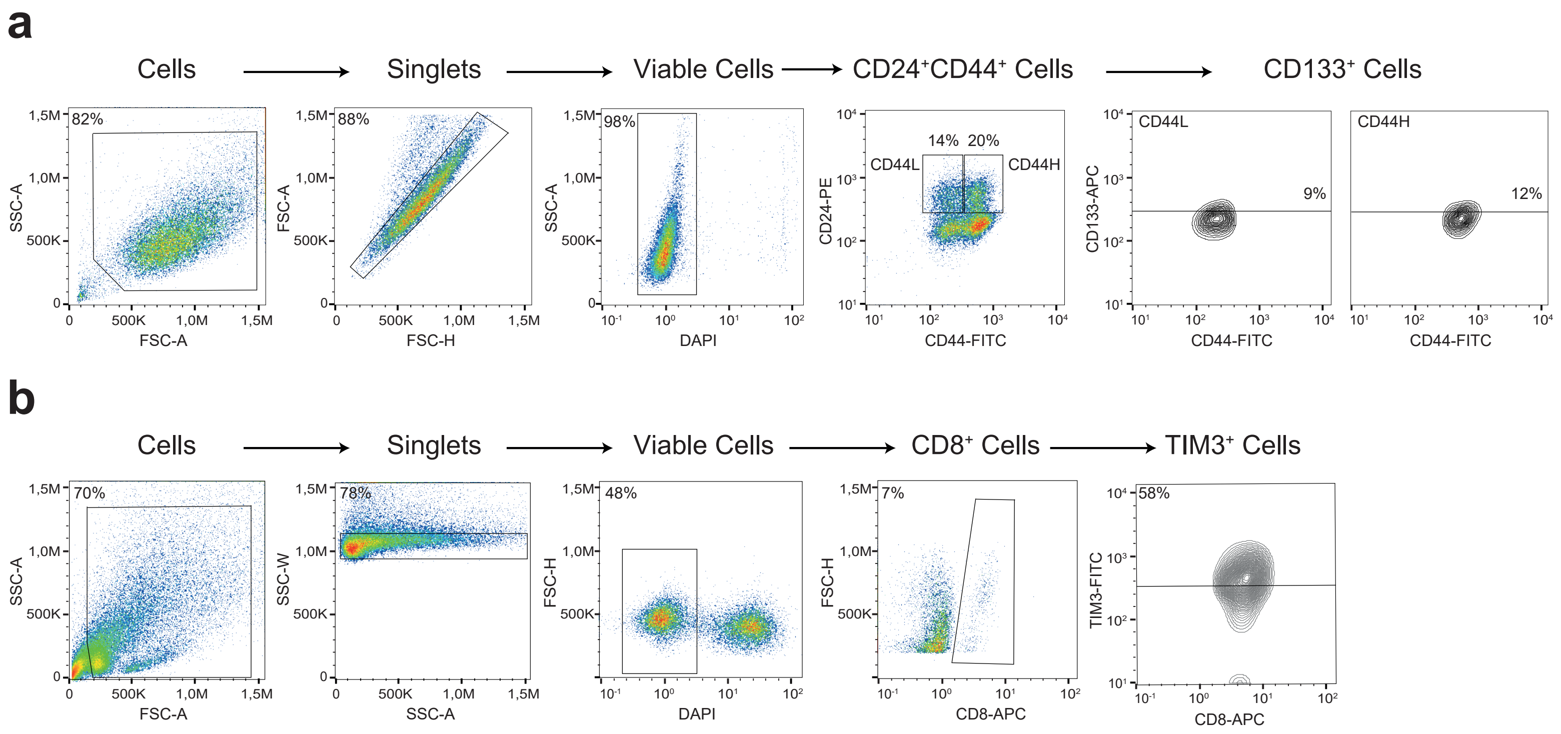

Supplementary Figure 1

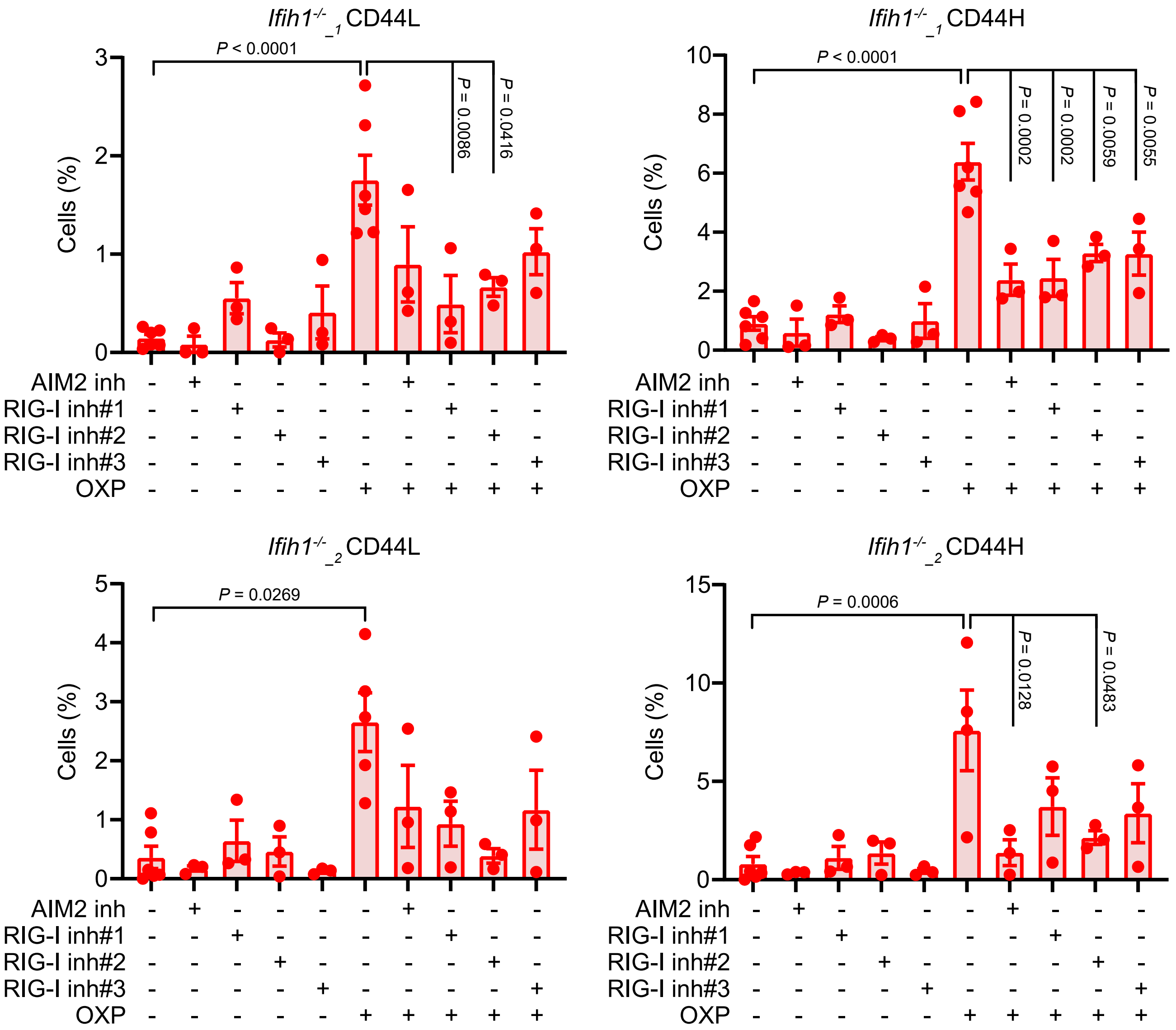

Supplementary Figure 2

a

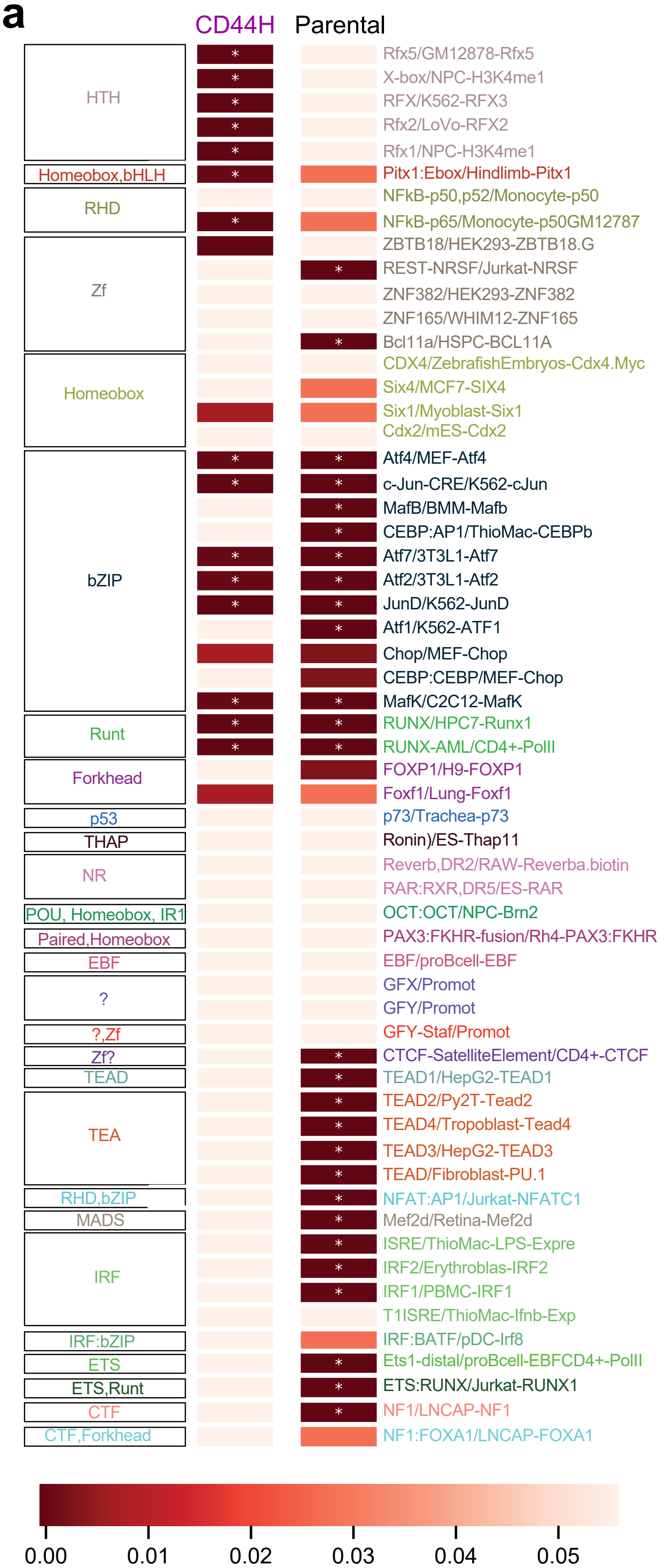

b

cell growth arrest
Kifc1,Cdk1,Ptgs2,Bub1,Bub1b,Cenpe,Ccnb1,Prr11,Aurka, Cdkn3,Plk1,Fhl1,Sema3a,Cth,Grem1,Sema3g
GO:0045786, GO:0030308

cell differentiation
Ptgs2,Kif14,Ccnb1,Ereg,Aurka,Grem1,Cdc20,Sema3a, Fam83d,Snai2,Sema3g
GO:0021700, GO:0048762

oxidative phosphorylation
Cdk1,Ccnb1
GO:1903862

protein dephosphorylation
Dusp5,Prkar2b,Errfi1,Plk1,Ptpn22,Psph,Inpp4b,Cdca2, Dusp5,Cdc25c,Cdkn3,Ptpn22
GO:0033673, GO:0016311

Type-I-IFN signaling
Oas3,Cd74,Oasl2,Bst2,Parp10,Irf7,Oas2,Irgm2,Zbp1, Rtp4,Trim12a,Bcl2l11,Dtx3l,Parp9,Ddx58
GO:0050792, GO:0009615

Type-II-IFN signaling
Gbp9,Irgm2,Gbp3,Parp14,Gbp7,Irgm1,Bst2,Parp9
GO:0034341

immune suppression
Gpnmb,Cd74,Grn,Nr1d1,Parp3
GO:0002695

APC machinery
H2-Q4,Tap1,Tapbp,H2-K1,H2-D1
GO:0019885

lipid metabolism
Irs1,Cpt1a,Cd74,Vdr,Wdr81,Slc27a1,C3/Nr1d1
GO:0019216

macroautophagy
Bmf,Cd74,Trp53inp1,Mmp9,Bcl2l11,Steap3,Fgfr1,Hsph1, Irgm2,Trim12a,Trp53inp1,Wdr81,Irgm1,Trp53inp2,Dcn, Map1lc3b
GO:2001233, GO:0006914

cell growth
Irs1,Sox9,Fgfr1
GO:0010464

cell stemness
Sox9,Klf10,Sox4
GO:0035019

tissue remodeling
Gpnmb,Vdr,Mmp9,Sema3c,Tpp1,P2rx7,Mmp2
GO:0048771

DNA damage response (stress response)
Dtx3l,Steap3,Parp9,Parp3
GO:2001022

chromatin binding
Dtx3l,Parp9
GO:0035563

Supplementary Figure 3

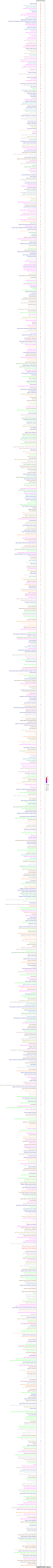

Supplementary Figure 4

Supplementary Table 1. *P* values and sample size for qRT-PCR studies.

**Figure 1b**		
**CD133$^-$**		
Klf4	<0,0001	CTR *n* = 3, IFN *n* = 3
Myc	0,4125	CTR *n* = 3, IFN *n* = 3
Oct3/4	0,0079	CTR *n* = 3, IFN *n* = 3
Sox2	0,0129	CTR *n* = 3, IFN *n* = 3
Nanog	<0,0001	CTR *n* = 3, IFN *n* = 3
Hes1	0,3648	CTR *n* = 3, IFN *n* = 3
Nes	0,0035	CTR *n* = 3, IFN *n* = 3
**CD133$^+$**		
Klf4	0,0755	CTR *n* = 3, IFN *n* = 3
Myc	0,314	CTR *n* = 3, IFN *n* = 3
Oct3/4	0,201	CTR *n* = 3, IFN *n* = 3
Sox2	0,4115	CTR *n* = 2, IFN *n* = 2
Nanog	0,0004	CTR *n* = 3, IFN *n* = 2
Hes1	0,1664	CTR *n* = 3, IFN *n* = 2
Nes	0,3223	CTR *n* = 3, IFN *n* = 2
**Figure 1d**		
Klf4	0,0001	CTR *n* = 4, IFN *n* = 3
Myc	0,111	CTR *n* = 3, IFN *n* = 3
Oct3/4	<0,0001	CTR *n* = 3, IFN *n* = 4
Sox2	0,0008	CTR *n* = 3, IFN *n* = 4
Nanog	0,002	CTR *n* = 3, IFN *n* = 3
Hes1	0,0001	CTR *n* = 3, IFN *n* = 3
Nes	0,0195	CTR *n* = 3, IFN *n* = 3
**Figure 2c**		
Klf4		
Wt_1		
IFNs-I	0,0100	CTR *n* = 2, IFN *n* = 2
OXP 3 µM	0,0185	CTR *n* = 2, OXP *n* = 2
OXP 30 µM	0,0080	CTR *n* = 2, OXP *n* = 2
OXP 300 µM	0,4171	CTR *n* = 2, OXP *n* = 2
Wt_2		
IFNs-I	0,0116	CTR *n* = 2, IFN *n* = 2
OXP 3 µM	0,0002	CTR *n* = 2, OXP *n* = 2
OXP 30 µM	<0,0001	CTR *n* = 2, OXP *n* = 2
OXP 300 µM	0,0784	CTR *n* = 2, OXP *n* = 2
Ifnar1$^{-/-}$_1		
IFNs-I	0,6759	CTR *n* = 2, IFN *n* = 2
OXP 3 µM	0,8610	CTR *n* = 2, OXP *n* = 2
OXP 30 µM	0,4078	CTR *n* = 2, OXP *n* = 2
OXP 300 µM	0,9175	CTR *n* = 2, OXP *n* = 2
Ifnar1$^{-/-}$_2		
IFNs-I	0,8839	CTR *n* = 2, IFN *n* = 2
OXP 3 µM	0,8716	CTR *n* = 2, OXP *n* = 2
OXP 30 µM	0,8765	CTR *n* = 2, OXP *n* = 2

OXP 300 µM	0,9081	CTR *n* = 2, OXP *n* = 2
Sting1 $^{-/-}$		
IFNs-I	0,1532	CTR *n* = 2, IFN *n* = 2
OXP 3 µM	0,6202	CTR *n* = 2, OXP *n* = 2
OXP 30 µM	0,1547	CTR *n* = 2, OXP *n* = 2
OXP 300 µM	0,9132	CTR *n* = 2, OXP *n* = 2
Tlr3 $^{-/-}$ ***_1***		
IFNs-I	0,0123	CTR *n* = 2, IFN *n* = 2
OXP 3 µM	0,6546	CTR *n* = 2, OXP *n* = 2
OXP 30 µM	0,7738	CTR *n* = 2, OXP *n* = 2
OXP 300 µM	0,0041	CTR *n* = 2, OXP *n* = 2
Tlr3 $^{-/-}$ ***_2***		
IFNs-I	0,9175	CTR *n* = 2, IFN *n* = 2
OXP 3 µM	0,2811	CTR *n* = 2, OXP *n* = 2
OXP 30 µM	0,3591	CTR *n* = 2, OXP *n* = 2
OXP 300 µM	0,3376	CTR *n* = 2, OXP *n* = 2
Ticam1 $^{-/-}$ ***_1***		
IFNs-I	0,0119	CTR *n* = 2, IFN *n* = 2
OXP 3 µM	0,0514	CTR *n* = 2, OXP *n* = 2
OXP 30 µM	0,4147	CTR *n* = 2, OXP *n* = 2
OXP 300 µM	0,0055	CTR *n* = 2, OXP *n* = 2
Ticam1 $^{-/-}$ ***_2***		
IFNs-I	0,0402	CTR *n* = 2, IFN *n* = 2
OXP 3 µM	0,1353	CTR *n* = 2, OXP *n* = 2
OXP 30 µM	0,0670	CTR *n* = 2, OXP *n* = 2
OXP 300 µM	0,0688	CTR *n* = 2, OXP *n* = 2
Ifih1 $^{-/-}$ ***_1***		
IFNs-I	0,0098	CTR *n* = 2, IFN *n* = 2
OXP 3 µM	0,7395	CTR *n* = 2, OXP *n* = 2
OXP 30 µM	0,4738	CTR *n* = 2, OXP *n* = 2
OXP 300 µM	0,4842	CTR *n* = 2, OXP *n* = 2
Ifih1 $^{-/-}$ ***_2***		
IFNs-I	<0,0001	CTR *n* = 2, IFN *n* = 2
OXP 3 µM	0,0051	CTR *n* = 2, OXP *n* = 2
OXP 30 µM	0,3588	CTR *n* = 2, OXP *n* = 2
OXP 300 µM	<0,0001	CTR *n* = 2, OXP *n* = 2
Mavs $^{-/-}$ ***_1***		
IFNs-I	0,0392	CTR *n* = 2, IFN *n* = 2
OXP 3 µM	0,7080	CTR *n* = 2, OXP *n* = 2
OXP 30 µM	0,9883	CTR *n* = 2, OXP *n* = 2
OXP 300 µM	0,0014	CTR *n* = 2, OXP *n* = 2
Mavs $^{-/-}$ ***_2***		
IFNs-I	0,0068	CTR *n* = 2, IFN *n* = 2
OXP 3 µM	0,7433	CTR *n* = 2, OXP *n* = 2
OXP 30 µM	0,8319	CTR *n* = 2, OXP *n* = 2
OXP 300 µM	0,0101	CTR *n* = 2, OXP *n* = 2
Myc		

Wt_1		
IFNs-I	0,0045	CTR *n* = 2, IFN *n* = 2
OXP 3 µM	0,0004	CTR *n* = 2, OXP *n* = 2
OXP 30 µM	0,0134	CTR *n* = 2, OXP *n* = 2
OXP 300 µM	0,8981	CTR *n* = 2, OXP *n* = 2
Wt_2		
IFNs-I	0,0006	CTR *n* = 2, IFN *n* = 2
OXP 3 µM	<0,0001	CTR *n* = 2, OXP *n* = 2
OXP 30 µM	<0,0001	CTR *n* = 2, OXP *n* = 2
OXP 300 µM	0,8270	CTR *n* = 2, OXP *n* = 2
Ifnar1$^{-/-}$_1		
IFNs-I	0,6779	CTR *n* = 2, IFN *n* = 2
OXP 3 µM	0,7380	CTR *n* = 2, OXP *n* = 2
OXP 30 µM	0,0627	CTR *n* = 2, OXP *n* = 2
OXP 300 µM	0,8643	CTR *n* = 2, OXP *n* = 2
Ifnar1$^{-/-}$_2		
IFNs-I	0,0818	CTR *n* = 2, IFN *n* = 2
OXP 3 µM	0,2561	CTR *n* = 2, OXP *n* = 2
OXP 30 µM	0,3276	CTR *n* = 2, OXP *n* = 2
OXP 300 µM	0,7825	CTR *n* = 2, OXP *n* = 2
Sting1$^{-/-}$		
IFNs-I	0,2802	CTR *n* = 2, IFN *n* = 2
OXP 3 µM	0,2298	CTR *n* = 2, OXP *n* = 2
OXP 30 µM	0,1842	CTR *n* = 2, OXP *n* = 2
OXP 300 µM	0,5089	CTR *n* = 2, OXP *n* = 2
Tlr3$^{-/-}$_1		
IFNs-I	0,0379	CTR *n* = 2, IFN *n* = 2
OXP 3 µM	0,0468	CTR *n* = 2, OXP *n* = 2
OXP 30 µM	0,2349	CTR *n* = 2, OXP *n* = 2
OXP 300 µM	0,0036	CTR *n* = 2, OXP *n* = 2
Tlr3$^{-/-}$_2		
IFNs-I	0,9322	CTR *n* = 2, IFN *n* = 2
OXP 3 µM	0,9840	CTR *n* = 2, OXP *n* = 2
OXP 30 µM	0,9586	CTR *n* = 2, OXP *n* = 2
OXP 300 µM	0,0003	CTR *n* = 2, OXP *n* = 2
Ticam1$^{-/-}$_1		
IFNs-I	0,9284	CTR *n* = 2, IFN *n* = 2
OXP 3 µM	0,1554	CTR *n* = 2, OXP *n* = 2
OXP 30 µM	0,8954	CTR *n* = 2, OXP *n* = 2
OXP 300 µM	0,0010	CTR *n* = 2, OXP *n* = 2
Ticam1$^{-/-}$_2		
IFNs-I	0,0032	CTR *n* = 2, IFN *n* = 2
OXP 3 µM	0,0626	CTR *n* = 2, OXP *n* = 2
OXP 30 µM	0,0814	CTR *n* = 2, OXP *n* = 2
OXP 300 µM	0,8608	CTR *n* = 2, OXP *n* = 2
Ifih1$^{-/-}$_1		
IFNs-I	0,0088	CTR *n* = 2, IFN *n* = 2

OXP 3 µM	0,4611	CTR *n* = 2, OXP *n* = 2
OXP 30 µM	0,3180	CTR *n* = 2, OXP *n* = 2
OXP 300 µM	0,1003	CTR *n* = 2, OXP *n* = 2
Ifih1$^{-/-}$_2		
IFNs-I	0,0057	CTR *n* = 2, IFN *n* = 2
OXP 3 µM	0,0175	CTR *n* = 2, OXP *n* = 2
OXP 30 µM	0,5570	CTR *n* = 2, OXP *n* = 2
OXP 300 µM	0,0175	CTR *n* = 2, OXP *n* = 2
Mavs$^{-/-}$_1		
IFNs-I	0,0516	CTR *n* = 2, IFN *n* = 2
OXP 3 µM	0,5869	CTR *n* = 2, OXP *n* = 2
OXP 30 µM	0,8687	CTR *n* = 2, OXP *n* = 2
OXP 300 µM	0,0104	CTR *n* = 2, OXP *n* = 2
Mavs$^{-/-}$_2		
IFNs-I	0,5921	CTR *n* = 2, IFN *n* = 2
OXP 3 µM	0,1304	CTR *n* = 2, OXP *n* = 2
OXP 30 µM	0,2390	CTR *n* = 2, OXP *n* = 2
OXP 300 µM	0,0068	CTR *n* = 2, OXP *n* = 2
Oct3/4		
Wt_1		
IFNs-I	0,0162	CTR *n* = 2, IFN *n* = 2
OXP 3 µM	0,0087	CTR *n* = 2, OXP *n* = 2
OXP 30 µM	0,0004	CTR *n* = 2, OXP *n* = 2
OXP 300 µM	0,0002	CTR *n* = 2, OXP *n* = 2
Wt_2		
IFNs-I	0,0039	CTR *n* = 2, IFN *n* = 2
OXP 3 µM	<0,0001	CTR *n* = 2, OXP *n* = 2
OXP 30 µM	<0,0001	CTR *n* = 2, OXP *n* = 2
OXP 300 µM	<0,0001	CTR *n* = 2, OXP *n* = 2
Ifnar1$^{-/-}$_1		
IFNs-I	0,3309	CTR *n* = 2, IFN *n* = 2
OXP 3 µM	0,0877	CTR *n* = 2, OXP *n* = 2
OXP 30 µM	0,4261	CTR *n* = 2, OXP *n* = 2
OXP 300 µM	>0,9999	CTR *n* = 2, OXP *n* = 2
Ifnar1$^{-/-}$_2		
IFNs-I	0,3896	CTR *n* = 2, IFN *n* = 2
OXP 3 µM	0,8105	CTR *n* = 2, OXP *n* = 2
OXP 30 µM	0,8340	CTR *n* = 2, OXP *n* = 2
OXP 300 µM	>0,9999	CTR *n* = 2, OXP *n* = 2
Sting1$^{-/-}$		
IFNs-I	0,4406	CTR *n* = 2, IFN *n* = 2
OXP 3 µM	0,4610	CTR *n* = 2, OXP *n* = 2
OXP 30 µM	0,1788	CTR *n* = 2, OXP *n* = 2
OXP 300 µM	0,0049	CTR *n* = 2, OXP *n* = 2
Tlr3$^{-/-}$_1		
IFNs-I	<0,0001	CTR *n* = 2, IFN *n* = 2
OXP 3 µM	0,0825	CTR *n* = 2, OXP *n* = 2

OXP 30 µM	0,0310	CTR *n* = 2, OXP *n* = 2
OXP 300 µM	0,0062	CTR *n* = 2, OXP *n* = 2
Tlr3$^{-/-}$_2		
IFNs-I	0,6993	CTR *n* = 2, IFN *n* = 2
OXP 3 µM	0,6342	CTR *n* = 2, OXP *n* = 2
OXP 30 µM	0,0580	CTR *n* = 2, OXP *n* = 2
OXP 300 µM	0,0017	CTR *n* = 2, OXP *n* = 2
Ticam1$^{-/-}$_1		
IFNs-I	0,0041	CTR *n* = 2, IFN *n* = 2
OXP 3 µM	0,0049	CTR *n* = 2, OXP *n* = 2
OXP 30 µM	0,0049	CTR *n* = 2, OXP *n* = 2
OXP 300 µM	0,8966	CTR *n* = 2, OXP *n* = 2
Ticam1$^{-/-}$_2		
IFNs-I	<0,0001	CTR *n* = 2, IFN *n* = 2
OXP 3 µM	<0,0001	CTR *n* = 2, OXP *n* = 2
OXP 30 µM	<0,0001	CTR *n* = 2, OXP *n* = 2
OXP 300 µM	0,0072	CTR *n* = 2, OXP *n* = 2
Ifih1$^{-/-}$_1		
IFNs-I	0,0080	CTR *n* = 2, IFN *n* = 2
OXP 3 µM	0,1846	CTR *n* = 2, OXP *n* = 2
OXP 30 µM	0,8198	CTR *n* = 2, OXP *n* = 2
OXP 300 µM	<0,0001	CTR *n* = 2, OXP *n* = 2
Ifih1$^{-/-}$_2		
IFNs-I	<0,0001	CTR *n* = 2, IFN *n* = 2
OXP 3 µM	<0,0001	CTR *n* = 2, OXP *n* = 2
OXP 30 µM	0,0019	CTR *n* = 2, OXP *n* = 2
OXP 300 µM	0,0005	CTR *n* = 2, OXP *n* = 2
Mavs$^{-/-}$_1		
IFNs-I	0,0299	CTR *n* = 2, IFN *n* = 2
OXP 3 µM	0,0686	CTR *n* = 2, OXP *n* = 2
OXP 30 µM	0,0238	CTR *n* = 2, OXP *n* = 2
OXP 300 µM	0,0005	CTR *n* = 2, OXP *n* = 2
Mavs$^{-/-}$_2		
IFNs-I	0,0441	CTR *n* = 2, IFN *n* = 2
OXP 3 µM	0,5620	CTR *n* = 2, OXP *n* = 2
OXP 30 µM	0,0279	CTR *n* = 2, OXP *n* = 2
OXP 300 µM	0,0075	CTR *n* = 2, OXP *n* = 2
Sox2		
Wt_1		
IFNs-I	0,0231	CTR *n* = 2, IFN *n* = 2
OXP 3 µM	0,0111	CTR *n* = 2, OXP *n* = 2
OXP 30 µM	0,0010	CTR *n* = 2, OXP *n* = 2
OXP 300 µM	0,0002	CTR *n* = 2, OXP *n* = 2
Wt_2		
IFNs-I	0,0027	CTR *n* = 2, IFN *n* = 2
OXP 3 µM	0,0004	CTR *n* = 2, OXP *n* = 2
OXP 30 µM	<0,0001	CTR *n* = 2, OXP *n* = 2

OXP 300 µM	0,0061	CTR *n* = 2, OXP *n* = 2
Ifnar1**$^{-/-}$**_1		
IFNs-I	0,1143	CTR *n* = 2, IFN *n* = 2
OXP 3 µM	0,1149	CTR *n* = 2, OXP *n* = 2
OXP 30 µM	0,0740	CTR *n* = 2, OXP *n* = 2
OXP 300 µM	0,5209	CTR *n* = 2, OXP *n* = 2
Ifnar1**$^{-/-}$**_2		
IFNs-I	0,2743	CTR *n* = 2, IFN *n* = 2
OXP 3 µM	0,3011	CTR *n* = 2, OXP *n* = 2
OXP 30 µM	0,8811	CTR *n* = 2, OXP *n* = 2
OXP 300 µM	0,3018	CTR *n* = 2, OXP *n* = 2
***Sting1**$^{-/-}$*		
IFNs-I	0,0189	CTR *n* = 2, IFN *n* = 2
OXP 3 µM	0,2952	CTR *n* = 2, OXP *n* = 2
OXP 30 µM	0,0913	CTR *n* = 2, OXP *n* = 2
OXP 300 µM	0,0002	CTR *n* = 2, OXP *n* = 2
Tlr3**$^{-/-}$**_1		
IFNs-I	0,9650	CTR *n* = 2, IFN *n* = 2
OXP 3 µM	0,7404	CTR *n* = 2, OXP *n* = 2
OXP 30 µM	0,8833	CTR *n* = 2, OXP *n* = 2
OXP 300 µM	0,4490	CTR *n* = 2, OXP *n* = 2
Tlr3**$^{-/-}$**_2		
IFNs-I	0,1818	CTR *n* = 2, IFN *n* = 2
OXP 3 µM	0,7997	CTR *n* = 2, OXP *n* = 2
OXP 30 µM	0,8234	CTR *n* = 2, OXP *n* = 2
OXP 300 µM	<0,0001	CTR *n* = 2, OXP *n* = 2
Ticam1**$^{-/-}$**_1		
IFNs-I	0,0498	CTR *n* = 2, IFN *n* = 2
OXP 3 µM	0,0024	CTR *n* = 2, OXP *n* = 2
OXP 30 µM	0,0124	CTR *n* = 2, OXP *n* = 2
OXP 300 µM	<0,0001	CTR *n* = 2, OXP *n* = 2
Ticam1**$^{-/-}$**_2		
IFNs-I	0,0013	CTR *n* = 2, IFN *n* = 2
OXP 3 µM	0,1773	CTR *n* = 2, OXP *n* = 2
OXP 30 µM	0,2323	CTR *n* = 2, OXP *n* = 2
OXP 300 µM	0,8169	CTR *n* = 2, OXP *n* = 2
Ifih1**$^{-/-}$**_1		
IFNs-I	0,0251	CTR *n* = 2, IFN *n* = 2
OXP 3 µM	0,9248	CTR *n* = 2, OXP *n* = 2
OXP 30 µM	0,9510	CTR *n* = 2, OXP *n* = 2
OXP 300 µM	0,0089	CTR *n* = 2, OXP *n* = 2
Ifih1**$^{-/-}$**_2		
IFNs-I	0,0006	CTR *n* = 2, IFN *n* = 2
OXP 3 µM	0,2741	CTR *n* = 2, OXP *n* = 2
OXP 30 µM	0,3492	CTR *n* = 2, OXP *n* = 2
OXP 300 µM	0,0002	CTR *n* = 2, OXP *n* = 2
Mavs**$^{-/-}$**_1		

IFNs-I	>0,9999	CTR *n* = 2, IFN *n* = 2
OXP 3 µM	0,6666	CTR *n* = 2, OXP *n* = 2
OXP 30 µM	>0,9999	CTR *n* = 2, OXP *n* = 2
OXP 300 µM	0,0380	CTR *n* = 2, OXP *n* = 2
Mavs $^{-/-}$***_2***		
IFNs-I	0,0853	CTR *n* = 2, IFN *n* = 2
OXP 3 µM	>0,9999	CTR *n* = 2, OXP *n* = 2
OXP 30 µM	>0,9999	CTR *n* = 2, OXP *n* = 2
OXP 300 µM	<0,0001	CTR *n* = 2, OXP *n* = 2
Nanog		
Wt_1		
IFNs-I	0,0029	CTR *n* = 2, IFN *n* = 2
OXP 3 µM	0,0009	CTR *n* = 2, OXP *n* = 2
OXP 30 µM	0,0027	CTR *n* = 2, OXP *n* = 2
OXP 300 µM	0,0001	CTR *n* = 2, OXP *n* = 2
Wt_2		
IFNs-I	0,0098	CTR *n* = 2, IFN *n* = 2
OXP 3 µM	0,0003	CTR *n* = 2, OXP *n* = 2
OXP 30 µM	<0,0001	CTR *n* = 2, OXP *n* = 2
OXP 300 µM	<0,0001	CTR *n* = 2, OXP *n* = 2
Ifnar1 $^{-/-}$***_1***		
IFNs-I	0,6839	CTR *n* = 2, IFN *n* = 2
OXP 3 µM	0,6876	CTR *n* = 2, OXP *n* = 2
OXP 30 µM	0,7227	CTR *n* = 2, OXP *n* = 2
OXP 300 µM	0,8034	CTR *n* = 2, OXP *n* = 2
Ifnar1 $^{-/-}$***_2***		
IFNs-I	0,9005	CTR *n* = 2, IFN *n* = 2
OXP 3 µM	0,9362	CTR *n* = 2, OXP *n* = 2
OXP 30 µM	0,9782	CTR *n* = 2, OXP *n* = 2
OXP 300 µM	0,8638	CTR *n* = 2, OXP *n* = 2
Sting1 $^{-/-}$		
IFNs-I	0,3157	CTR *n* = 2, IFN *n* = 2
OXP 3 µM	0,0881	CTR *n* = 2, OXP *n* = 2
OXP 30 µM	0,0171	CTR *n* = 2, OXP *n* = 2
OXP 300 µM	0,0013	CTR *n* = 2, OXP *n* = 2
Tlr3 $^{-/-}$***_1***		
IFNs-I	0,4037	CTR *n* = 2, IFN *n* = 2
OXP 3 µM	0,8491	CTR *n* = 2, OXP *n* = 2
OXP 30 µM	0,8828	CTR *n* = 2, OXP *n* = 2
OXP 300 µM	0,0148	CTR *n* = 2, OXP *n* = 2
Tlr3 $^{-/-}$***_2***		
IFNs-I	<0,0001	CTR *n* = 2, IFN *n* = 2
OXP 3 µM	0,1002	CTR *n* = 2, OXP *n* = 2
OXP 30 µM	0,8696	CTR *n* = 2, OXP *n* = 2
OXP 300 µM	0,0006	CTR *n* = 2, OXP *n* = 2
Ticam1 $^{-/-}$***_1***		
IFNs-I	0,1542	CTR *n* = 2, IFN *n* = 2

OXP 3 µM	0,0227	CTR *n* = 2, OXP *n* = 2
OXP 30 µM	0,0369	CTR *n* = 2, OXP *n* = 2
OXP 300 µM	0,0003	CTR *n* = 2, OXP *n* = 2
Ticam1**$^{-/-}$**_2		
IFNs-I	0,0078	CTR *n* = 2, IFN *n* = 2
OXP 3 µM	<0,0001	CTR *n* = 2, OXP *n* = 2
OXP 30 µM	0,0369	CTR *n* = 2, OXP *n* = 2
OXP 300 µM	0,0206	CTR *n* = 2, OXP *n* = 2
Ifih1**$^{-/-}$**_1		
IFNs-I	<0,0001	CTR *n* = 2, IFN *n* = 2
OXP 3 µM	>0,9999	CTR *n* = 2, OXP *n* = 2
OXP 30 µM	<0,0001	CTR *n* = 2, OXP *n* = 2
OXP 300 µM	<0,0001	CTR *n* = 2, OXP *n* = 2
Ifih1**$^{-/-}$**_2		
IFNs-I	<0,0001	CTR *n* = 2, IFN *n* = 2
OXP 3 µM	0,0293	CTR *n* = 2, OXP *n* = 2
OXP 30 µM	0,0122	CTR *n* = 2, OXP *n* = 2
OXP 300 µM	0,0001	CTR *n* = 2, OXP *n* = 2
Mavs**$^{-/-}$**_1		
IFNs-I	0,0073	CTR *n* = 2, IFN *n* = 2
OXP 3 µM	0,0008	CTR *n* = 2, OXP *n* = 2
OXP 30 µM	0,0063	CTR *n* = 2, OXP *n* = 2
OXP 300 µM	0,0008	CTR *n* = 2, OXP *n* = 2
Mavs**$^{-/-}$**_2		
IFNs-I	0,0007	CTR *n* = 2, IFN *n* = 2
OXP 3 µM	0,3210	CTR *n* = 2, OXP *n* = 2
OXP 30 µM	0,0002	CTR *n* = 2, OXP *n* = 2
OXP 300 µM	<0,0001	CTR *n* = 2, OXP *n* = 2
Hes1		
Wt_1		
IFNs-I	0,0001	CTR *n* = 2, IFN *n* = 2
OXP 3 µM	0,0004	CTR *n* = 2, OXP *n* = 2
OXP 30 µM	0,0003	CTR *n* = 2, OXP *n* = 2
OXP 300 µM	0,0004	CTR *n* = 2, OXP *n* = 2
Wt_2		
IFNs-I	0,0057	CTR *n* = 2, IFN *n* = 2
OXP 3 µM	0,0003	CTR *n* = 2, OXP *n* = 2
OXP 30 µM	0,6222	CTR *n* = 2, OXP *n* = 2
OXP 300 µM	0,00163	CTR *n* = 2, OXP *n* = 2
Ifnar1**$^{-/-}$**_1		
IFNs-I	0,7837	CTR *n* = 2, IFN *n* = 2
OXP 3 µM	0,4327	CTR *n* = 2, OXP *n* = 2
OXP 30 µM	0,6014	CTR *n* = 2, OXP *n* = 2
OXP 300 µM	0,8227	CTR *n* = 2, OXP *n* = 2
Ifnar1**$^{-/-}$**_2		
IFNs-I	0,4860	CTR *n* = 2, IFN *n* = 2
OXP 3 µM	0,8942	CTR *n* = 2, OXP *n* = 2

OXP 30 µM	0,8476	CTR *n* = 2, OXP *n* = 2
OXP 300 µM	0,8854	CTR *n* = 2, OXP *n* = 2
Sting1 $^{-/-}$		
IFNs-I	0,9055	CTR *n* = 2, IFN *n* = 2
OXP 3 µM	0,0315	CTR *n* = 2, OXP *n* = 2
OXP 30 µM	0,7626	CTR *n* = 2, OXP *n* = 2
OXP 300 µM	0,0070	CTR *n* = 2, OXP *n* = 2
Tlr3 $^{-/-}$***_1***		
IFNs-I	0,3077	CTR *n* = 2, IFN *n* = 2
OXP 3 µM	0,0501	CTR *n* = 2, OXP *n* = 2
OXP 30 µM	0,0290	CTR *n* = 2, OXP *n* = 2
OXP 300 µM	0,0062	CTR *n* = 2, OXP *n* = 2
Tlr3 $^{-/-}$***_2***		
IFNs-I	0,2161	CTR *n* = 2, IFN *n* = 2
OXP 3 µM	0,8679	CTR *n* = 2, OXP *n* = 2
OXP 30 µM	0,6074	CTR *n* = 2, OXP *n* = 2
OXP 300 µM	<0,0001	CTR *n* = 2, OXP *n* = 2
Ticam1 $^{-/-}$***_1***		
IFNs-I	0,0039	CTR *n* = 2, IFN *n* = 2
OXP 3 µM	0,1189	CTR *n* = 2, OXP *n* = 2
OXP 30 µM	0,7613	CTR *n* = 2, OXP *n* = 2
OXP 300 µM	0,0045	CTR *n* = 2, OXP *n* = 2
Ticam1 $^{-/-}$***_2***		
IFNs-I	0,0094	CTR *n* = 2, IFN *n* = 2
OXP 3 µM	0,5360	CTR *n* = 2, OXP *n* = 2
OXP 30 µM	0,6054	CTR *n* = 2, OXP *n* = 2
OXP 300 µM	0,0151	CTR *n* = 2, OXP *n* = 2
Ifih1 $^{-/-}$***_1***		
IFNs-I	0,0085	CTR *n* = 2, IFN *n* = 2
OXP 3 µM	0,8605	CTR *n* = 2, OXP *n* = 2
OXP 30 µM	0,0205	CTR *n* = 2, OXP *n* = 2
OXP 300 µM	0,0002	CTR *n* = 2, OXP *n* = 2
Ifih1 $^{-/-}$***_2***		
IFNs-I	<0,0001	CTR *n* = 2, IFN *n* = 2
OXP 3 µM	0,0075	CTR *n* = 2, OXP *n* = 2
OXP 30 µM	0,7466	CTR *n* = 2, OXP *n* = 2
OXP 300 µM	<0,0001	CTR *n* = 2, OXP *n* = 2
Mavs $^{-/-}$***_1***		
IFNs-I	0,0079	CTR *n* = 2, IFN *n* = 2
OXP 3 µM	0,3355	CTR *n* = 2, OXP *n* = 2
OXP 30 µM	0,0809	CTR *n* = 2, OXP *n* = 2
OXP 300 µM	0,0211	CTR *n* = 2, OXP *n* = 2
Mavs $^{-/-}$***_2***		
IFNs-I	0,4357	CTR *n* = 2, IFN *n* = 2
OXP 3 µM	0,0650	CTR *n* = 2, OXP *n* = 2
OXP 30 µM	0,0007	CTR *n* = 2, OXP *n* = 2
OXP 300 µM	0,0034	CTR *n* = 2, OXP *n* = 2

Nes		
Wt_1		
IFNs-I	0,0359	CTR *n* = 2, IFN *n* = 2
OXP 3 µM	0,0596	CTR *n* = 2, OXP *n* = 2
OXP 30 µM	0,0003	CTR *n* = 2, OXP *n* = 2
OXP 300 µM	0,2326	CTR *n* = 2, OXP *n* = 2
Wt_2		
IFNs-I	0,0157	CTR *n* = 2, IFN *n* = 2
OXP 3 µM	0,0007	CTR *n* = 2, OXP *n* = 2
OXP 30 µM	<0,0001	CTR *n* = 2, OXP *n* = 2
OXP 300 µM	0,2399	CTR *n* = 2, OXP *n* = 2
Ifnar1$^{-/-}$_1		
IFNs-I	0,0600	CTR *n* = 2, IFN *n* = 2
OXP 3 µM	0,6575	CTR *n* = 2, OXP *n* = 2
OXP 30 µM	0,5958	CTR *n* = 2, OXP *n* = 2
OXP 300 µM	0,1100	CTR *n* = 2, OXP *n* = 2
Ifnar1$^{-/-}$_2		
IFNs-I	0,8216	CTR *n* = 2, IFN *n* = 2
OXP 3 µM	0,9079	CTR *n* = 2, OXP *n* = 2
OXP 30 µM	0,8056	CTR *n* = 2, OXP *n* = 2
OXP 300 µM	0,8921	CTR *n* = 2, OXP *n* = 2
Sting1$^{-/-}$		
IFNs-I	0,6654	CTR *n* = 2, IFN *n* = 2
OXP 3 µM	0,6775	CTR *n* = 2, OXP *n* = 2
OXP 30 µM	0,6666	CTR *n* = 2, OXP *n* = 2
OXP 300 µM	0,7014	CTR *n* = 2, OXP *n* = 2
Tlr3$^{-/-}$_1		
IFNs-I	0,8550	CTR *n* = 2, IFN *n* = 2
OXP 3 µM	0,7707	CTR *n* = 2, OXP *n* = 2
OXP 30 µM	0,5145	CTR *n* = 2, OXP *n* = 2
OXP 300 µM	0,0065	CTR *n* = 2, OXP *n* = 2
Tlr3$^{-/-}$_2		
IFNs-I	0,4183	CTR *n* = 2, IFN *n* = 2
OXP 3 µM	0,1355	CTR *n* = 2, OXP *n* = 2
OXP 30 µM	0,1417	CTR *n* = 2, OXP *n* = 2
OXP 300 µM	0,0005	CTR *n* = 2, OXP *n* = 2
Ticam1$^{-/-}$_1		
IFNs-I	0,0010	CTR *n* = 2, IFN *n* = 2
OXP 3 µM	0,3177	CTR *n* = 2, OXP *n* = 2
OXP 30 µM	0,3857	CTR *n* = 2, OXP *n* = 2
OXP 300 µM	0,2265	CTR *n* = 2, OXP *n* = 2
Ticam1$^{-/-}$_2		
IFNs-I	0,0632	CTR *n* = 2, IFN *n* = 2
OXP 3 µM	0,3912	CTR *n* = 2, OXP *n* = 2
OXP 30 µM	0,2280	CTR *n* = 2, OXP *n* = 2
OXP 300 µM	0,7776	CTR *n* = 2, OXP *n* = 2
Ifih1$^{-/-}$_1		

IFNs-I	0,0005	CTR *n* = 2, IFN *n* = 2
OXP 3 µM	>0,9999	CTR *n* = 2, OXP *n* = 2
OXP 30 µM	>0,9999	CTR *n* = 2, OXP *n* = 2
OXP 300 µM	0,0005	CTR *n* = 2, OXP *n* = 2
Ifih1$^{-/-}$_2		
IFNs-I	0,0013	CTR *n* = 2, IFN *n* = 2
OXP 3 µM	0,1339	CTR *n* = 2, OXP *n* = 2
OXP 30 µM	0,9877	CTR *n* = 2, OXP *n* = 2
OXP 300 µM	0,3768	CTR *n* = 2, OXP *n* = 2
Mavs$^{-/-}$_1		
IFNs-I	0,5814	CTR *n* = 2, IFN *n* = 2
OXP 3 µM	0,0159	CTR *n* = 2, OXP *n* = 2
OXP 30 µM	0,1429	CTR *n* = 2, OXP *n* = 2
OXP 300 µM	0,0089	CTR *n* = 2, OXP *n* = 2
Mavs$^{-/-}$_2		
IFNs-I	0,8998	CTR *n* = 2, IFN *n* = 2
OXP 3 µM	0,5690	CTR *n* = 2, OXP *n* = 2
OXP 30 µM	0,5060	CTR *n* = 2, OXP *n* = 2
OXP 300 µM	0,0080	CTR *n* = 2, OXP *n* = 2
**Figure 2d**		
**MCA205**		
Klf4		
DOX 0,25 µM	<0,0001	CTR *n* = 3, DOX *n* = 3
DOX 2,5 µM	<0,0001	CTR *n* = 3, DOX *n* = 3
DOX 25 µM	<0,0001	CTR *n* = 3, DOX *n* = 3
OXP 3 µM	<0,000	CTR *n* = 3, OXP *n* = 3
OXP 30 µM	0,0006	CTR *n* = 3, OXP *n* = 3
OXP 300 µM	0,0008	CTR *n* = 3, OXP *n* = 3
CDDP 1,5 µM	0,9865	CTR *n* = 3, CDDP *n* = 3
CDDP 15 µM	0,0995	CTR *n* = 3, CDDP *n* = 3
CDDP 150 µM	0,8222	CTR *n* = 3, CDDP *n* = 3
Myc		
DOX 0,25 µM	0,1271	CTR *n* = 3, DOX *n* = 3
DOX 2,5 µM	0,0378	CTR *n* = 3, DOX *n* = 3
DOX 25 µM	0,3729	CTR *n* = 3, DOX *n* = 3
OXP 3 µM	0,0019	CTR *n* = 3, OXP *n* = 3
OXP 30 µM	0,0006	CTR *n* = 3, OXP *n* = 3
OXP 300 µM	0,067	CTR *n* = 3, OXP *n* = 3
CDDP 1,5 µM	0,7514	CTR *n* = 3, CDDP *n* = 3
CDDP 15 µM	0,73	CTR *n* = 3, CDDP *n* = 3
CDDP 150 µM	0,7809	CTR *n* = 3, CDDP *n* = 3
Oct3/4		
DOX 0,25 µM	0,041	CTR *n* = 3, DOX *n* = 3
DOX 2,5 µM	0,0045	CTR *n* = 3, DOX *n* = 3
DOX 25 µM	0,0015	CTR *n* = 3, DOX *n* = 3
OXP 3 µM	0,0081	CTR *n* = 3, OXP *n* = 3
OXP 30 µM	0,0009	CTR *n* = 3, OXP *n* = 3

OXP 300 µM	<0,0001	CTR *n* = 3, OXP *n* = 3
CDDP 1,5 µM	0,9696	CTR *n* = 3, CDDP *n* = 3
CDDP 15 µM	0,0023	CTR *n* = 3, CDDP *n* = 3
CDDP 150 µM	0,0216	CTR *n* = 3, CDDP *n* = 3
Sox2		
DOX 0,25 µM	0,011	CTR *n* = 3, DOX *n* = 3
DOX 2,5 µM	0,0004	CTR *n* = 3, DOX *n* = 3
DOX 25 µM	0,0002	CTR *n* = 3, DOX *n* = 3
OXP 3 µM	0,0249	CTR *n* = 3, OXP *n* = 3
OXP 30 µM	0,0019	CTR *n* = 3, OXP *n* = 3
OXP 300 µM	<0,0001	CTR *n* = 3, OXP *n* = 3
CDDP 1,5 µM	0,8696	CTR *n* = 3, CDDP *n* = 3
CDDP 15 µM	0,0127	CTR *n* = 3, CDDP *n* = 3
CDDP 150 µM	0,7231	CTR *n* = 3, CDDP *n* = 3
Nanog		
DOX 0,25 µM	0,1149	CTR *n* = 3, DOX *n* = 3
DOX 2,5 µM	0,0214	CTR *n* = 3, DOX *n* = 3
DOX 25 µM	0,0103	CTR *n* = 3, DOX *n* = 3
OXP 3 µM	<0,0001	CTR *n* = 3, OXP *n* = 3
OXP 30 µM	0,3215	CTR *n* = 3, OXP *n* = 3
OXP 300 µM	0,9999	CTR *n* = 3, OXP *n* = 3
CDDP 1,5 µM	0,4971	CTR *n* = 3, CDDP *n* = 3
CDDP 15 µM	0,2518	CTR *n* = 3, CDDP *n* = 3
CDDP 150 µM	0,8603	CTR *n* = 3, CDDP *n* = 3
Hes1		
DOX 0,25 µM	0,001	CTR *n* = 3, DOX *n* = 3
DOX 2,5 µM	0,0012	CTR *n* = 3, DOX *n* = 3
DOX 25 µM	<0,0001	CTR *n* = 3, DOX *n* = 3
OXP 3 µM	0,0574	CTR *n* = 3, OXP *n* = 3
OXP 30 µM	0,0031	CTR *n* = 3, OXP *n* = 3
OXP 300 µM	<0,0001	CTR *n* = 3, OXP *n* = 3
CDDP 1,5 µM	0,7941	CTR *n* = 3, CDDP *n* = 3
CDDP 15 µM	0,0044	CTR *n* = 3, CDDP *n* = 3
CDDP 150 µM	0,7171	CTR *n* = 3, CDDP *n* = 3
Nes		
DOX 0,25 µM	<0,0001	CTR *n* = 3, DOX *n* = 3
DOX 2,5 µM	0,0262	CTR *n* = 3, DOX *n* = 3
DOX 25 µM	0,1079	CTR *n* = 3, DOX *n* = 3
OXP 3 µM	0,0039	CTR *n* = 3, OXP *n* = 3
OXP 30 µM	0,0403	CTR *n* = 3, OXP *n* = 3
OXP 300 µM	0,595	CTR *n* = 3, OXP *n* = 3
CDDP 1,5 µM	0,8662	CTR *n* = 3, CDDP *n* = 3
CDDP 15 µM	0,6453	CTR *n* = 3, CDDP *n* = 3
CDDP 150 µM	0,8485	CTR *n* = 3, CDDP *n* = 3
**AT3**		
Klf4		
DOX 0,25 µM	0,067	CTR *n* = 3, DOX *n* = 3

DOX 2,5 µM	0,0014	CTR *n* = 3, DOX *n* = 3
DOX 25 µM	<0,0001	CTR *n* = 3, DOX *n* = 3
OXP 3 µM	<0,0001	CTR *n* = 3, OXP *n* = 5
OXP 30 µM	0,9423	CTR *n* = 3, OXP *n* = 4
OXP 300 µM	0,6838	CTR *n* = 3, OXP *n* = 4
CDDP 1,5 µM	0,4944	CTR *n* = 3, CDDP *n* = 3
CDDP 15 µM	0,0113	CTR *n* = 3, CDDP *n* = 3
CDDP 150 µM	0,7844	CTR *n* = 3, CDDP *n* = 3
Myc		
DOX 0,25 µM	0,1812	CTR *n* = 3, DOX *n* = 3
DOX 2,5 µM	0,0383	CTR *n* = 3, DOX *n* = 3
DOX 25 µM	0,8548	CTR *n* = 3, DOX *n* = 3
OXP 3 µM	0,0065	CTR *n* = 3, OXP *n* = 4
OXP 30 µM	0,5783	CTR *n* = 3, OXP *n* = 4
OXP 300 µM	0,6602	CTR *n* = 3, OXP *n* = 3
CDDP 1,5 µM	0,9675	CTR *n* = 3, CDDP *n* = 2
CDDP 15 µM	0,9676	CTR *n* = 3, CDDP *n* = 2
CDDP 150 µM	0,8022	CTR *n* = 3, CDDP *n* = 3
Oct3/4		
DOX 0,25 µM	0,0063	CTR *n* = 3, DOX *n* = 3
DOX 2,5 µM	<0,0001	CTR *n* = 3, DOX *n* = 3
DOX 25 µM	0,0567	CTR *n* = 3, DOX *n* = 2
OXP 3 µM	<0,0001	CTR *n* = 3, OXP *n* = 3
OXP 30 µM	0,2527	CTR *n* = 3, OXP *n* = 3
OXP 300 µM	0,9604	CTR *n* = 3, OXP *n* = 3
CDDP 1,5 µM	0,7402	CTR *n* = 3, CDDP *n* = 3
CDDP 15 µM	0,0204	CTR *n* = 3, CDDP *n* = 3
CDDP 150 µM	0,8882	CTR *n* = 3, CDDP *n* = 3
Sox2		
DOX 0,25 µM	0,1149	CTR *n* = 3, DOX *n* = 3
DOX 2,5 µM	0,0214	CTR *n* = 3, DOX *n* = 3
DOX 25 µM	0,0103	CTR *n* = 3, DOX *n* = 2
OXP 3 µM	<0,0001	CTR *n* = 3, OXP *n* = 4
OXP 30 µM	0,3215	CTR *n* = 3, OXP *n* = 4
OXP 300 µM	0,9999	CTR *n* = 3, OXP *n* = 3
CDDP 1,5 µM	0,4971	CTR *n* = 3, CDDP *n* = 3
CDDP 15 µM	0,2518	CTR *n* = 3, CDDP *n* = 3
CDDP 150 µM	0,8603	CTR *n* = 3, CDDP *n* = 3
Nanog		
DOX 0,25 µM	0,0342	CTR *n* = 3, DOX *n* = 3
DOX 2,5 µM	0,0171	CTR *n* = 3, DOX *n* = 3
DOX 25 µM	0,0028	CTR *n* = 3, DOX *n* = 2
OXP 3 µM	0,0001	CTR *n* = 3, OXP *n* = 5
OXP 30 µM	0,0892	CTR *n* = 3, OXP *n* = 3
OXP 300 µM	0,3257	CTR *n* = 3, OXP *n* = 5
CDDP 1,5 µM	0,3353	CTR *n* = 3, CDDP *n* = 3
CDDP 15 µM	0,1928	CTR *n* = 3, CDDP *n* = 2

CDDP 150 µM	0,8114	CTR *n* = 3, CDDP *n* = 3
Hes1		
DOX 0,25 µM	0,1918	CTR *n* = 3, DOX *n* = 3
DOX 2,5 µM	0,0864	CTR *n* = 3, DOX *n* = 3
DOX 25 µM	0,1437	CTR *n* = 3, DOX *n* = 2
OXP 3 µM	0,0256	CTR *n* = 3, OXP *n* = 4
OXP 30 µM	0,161	CTR *n* = 3, OXP *n* = 4
OXP 300 µM	0,5915	CTR *n* = 3, OXP *n* = 4
CDDP 1,5 µM	0,0752	CTR *n* = 3, CDDP *n* = 2
CDDP 15 µM	0,3308	CTR *n* = 3, CDDP *n* = 2
CDDP 150 µM	0,7781	CTR *n* = 3, CDDP *n* = 3
Nes		
DOX 0,25 µM	0,577	CTR *n* = 3, DOX *n* = 3
DOX 2,5 µM	0,8032	CTR *n* = 3, DOX *n* = 3
DOX 25 µM	<0,0001	CTR *n* = 3, DOX *n* = 2
OXP 3 µM	<0,0001	CTR *n* = 3, OXP *n* = 3
OXP 30 µM	<0,0001	CTR *n* = 3, OXP *n* = 3
OXP 300 µM	<0,0001	CTR *n* = 3, OXP *n* = 3
CDDP 1,5 µM	0,8467	CTR *n* = 3, CDDP *n* = 3
CDDP 15 µM	0,9085	CTR *n* = 3, CDDP *n* = 3
CDDP 150 µM	0,7936	CTR *n* = 3, CDDP *n* = 3
**Figure 3d**		
Klf4		
CytoD	>0,9999	CTR *n* = 3, CytoD *n* = 2
EVs	>0,9999	CTR *n* = 3, EVs *n* = 3
EVs+CytoD	>0,9999<br>>0,9999 (*vs* EVs)	CTR *n* = 3, EVs+CytoD *n* = 2
Myc		
CytoD	>0,9999	CTR *n* = 3, CytoD *n* = 2
EVs	0,0081	CTR *n* = 3, EVs *n* = 3
EVs+CytoD	>0,9999<br>0,0082 (*vs* EVs)	CTR *n* = 3, EVs+CytoD *n* = 2
Oct3/4		
CytoD	>0,9999	CTR *n* = 3, CytoD *n* = 2
EVs	0,0004	CTR *n* = 3, EVs *n* = 3
EVs+CytoD	0,7715<br>0,003 (*vs* EVs)	CTR *n* = 3, EVs+CytoD *n* = 2
Sox2		
CytoD	>0,9999	CTR *n* = 3, CytoD *n* = 2
EVs	>0,9999	CTR *n* = 3, EVs *n* = 3
EVs+CytoD	>0,9999<br>>0,9999 (*vs* EVs)	CTR *n* = 3, EVs+CytoD *n* = 2
Nanog		
CytoD	>0,9999	CTR *n* = 3, CytoD *n* = 2
EVs	<0,0001	CTR *n* = 3, EVs *n* = 3
EVs+CytoD	<0,0001<br><0,0001 (*vs* EVs)	CTR *n* = 3, EVs+CytoD *n* = 2
Hes1		

CytoD	>0,9999	CTR *n* = 3, CytoD *n* = 2
EVs	0,1292	CTR *n* = 3, EVs *n* = 3
EVs+CytoD	>0,9999<br>0,1031 (*vs* EVs)	CTR *n* = 3, EVs+CytoD *n* = 2
Nes		
CytoD	>0,9999	CTR *n* = 3, CytoD *n* = 2
EVs	>0,9999	CTR *n* = 3, EVs *n* = 3
EVs+CytoD	>0,9999<br>>0,9999 (*vs* EVs)	CTR *n* = 3, EVs+CytoD *n* = 2
**Figure 3e**		
Klf4	0,2313	CTR *n* = 4, OXP *n* = 4
Myc	0,0033	CTR *n* = 4, OXP *n* = 4
Oct3/4	<0,0001	CTR *n* = 3, OXP *n* = 4
Sox2	<0,0001	CTR *n* = 4, OXP *n* = 4
Nanog	<0,0001	CTR *n* = 4, OXP *n* = 4
Hes1	<0,0001	CTR *n* = 3, OXP *n* = 4
Nes	0,0314	CTR *n* = 4, OXP *n* = 4
Snail	0,1421	CTR *n* = 4, OXP *n* = 4
Twist	0,0039	CTR *n* = 4, OXP *n* = 4
Ncad	0,4687	CTR *n* = 2, OXP *n* = 4
Vim	0,7955	CTR *n* = 3, OXP *n* = 3
Fn1	0,2581	CTR *n* = 4, OXP *n* = 4
Pdl1	0,0003	CTR *n* = 6, OXP *n* = 9
Pdcd1lg2	<0,0001	CTR *n* = 6, OXP *n* = 7
Lgals9	0,0198	CTR *n* = 6, OXP *n* = 10
Ifnb1	<0,0001	CTR *n* = 4, OXP *n* = 4
**Extended Data Figure 6b**		
**MCA205**		
Klf4		CTR *n*=6
PAR *vs. Kdm1b*^{OVER}	0,9371	*Kdm1b*^{OVER} *n*=4
PAR *vs. Kdm1b*^{KD}	>0,9999	*Kdm1b*^{KD} *n*=4
Myc		CTR *n*=6
PAR *vs. Kdm1b*^{OVER}	>0,9999	*Kdm1b*^{OVER} *n*=3
PAR *vs. Kdm1b*^{KD}	>0,9999	*Kdm1b*^{KD} *n*=4
Oct3/4		CTR *n*=9
PAR *vs. Kdm1b*^{OVER}	0,4129	*Kdm1b*^{OVER} *n*=4
PAR *vs. Kdm1b*^{KD}	>0,9999	*Kdm1b*^{KD} *n*=4
Sox2		CTR *n*=9
PAR *vs. Kdm1b*^{OVER}	0,1063	*Kdm1b*^{OVER} *n*=3
PAR *vs. Kdm1b*^{KD}	>0,9999	*Kdm1b*^{KD} *n*=4
Nanog		CTR *n*=8
PAR *vs. Kdm1b*^{OVER}	<0,0001	*Kdm1b*^{OVER} *n*=4
PAR *vs. Kdm1b*^{KD}	>0,9999	*Kdm1b*^{KD} *n*=4
Hes1		CTR *n*=8
PAR *vs. Kdm1b*^{OVER}	0,9983	*Kdm1b*^{OVER} *n*=4
PAR *vs. Kdm1b*^{KD}	>0,9999	*Kdm1b*^{KD} *n*=4
Nes		CTR *n*=5
PAR *vs. Kdm1b*^{OVER}	0,0847	*Kdm1b*^{OVER} *n*=4
PAR *vs. Kdm1b*^{KD}	>0,9999	*Kdm1b*^{KD} *n*=4

**CT26**		
Klf4		CTR *n*=3
PAR *vs. Kdm1b*^{OVER}	>0,9999	*Kdm1b*^{OVER} *n*=4
PAR *vs. Kdm1b*^{KD}	>0,9999	*Kdm1b*^{KD} *n*=4
Myc		CTR *n*=4
PAR *vs. Kdm1b*^{OVER}	>0,9999	*Kdm1b*^{OVER} *n*=4
PAR *vs. Kdm1b*^{KD}	>0,9999	*Kdm1b*^{KD} *n*=4
Oct3/4		CTR *n*=5
PAR *vs. Kdm1b*^{OVER}	>0,9999	*Kdm1b*^{OVER} *n*=4
PAR *vs. Kdm1b*^{KD}	0,5142	*Kdm1b*^{KD} *n*=3
Sox2		CTR *n*=4
PAR *vs. Kdm1b*^{OVER}	0,4328	*Kdm1b*^{OVER} *n*=4
PAR *vs. Kdm1b*^{KD}	>0,9999	*Kdm1b*^{KD} *n*=4
Nanog		CTR *n*=6
PAR *vs. Kdm1b*^{OVER}	>0,9999	*Kdm1b*^{OVER} *n*=4
PAR *vs. Kdm1b*^{KD}	>0,9999	*Kdm1b*^{KD} *n*=4
Hes1		CTR *n*=6
PAR *vs. Kdm1b*^{OVER}	0,5399	*Kdm1b*^{OVER} *n*=4
PAR *vs. Kdm1b*^{KD}	>0,9999	*Kdm1b*^{KD} *n*=4
Nes		CTR *n*=3
PAR *vs. Kdm1b*^{OVER}	0,4001	*Kdm1b*^{OVER} *n*=4
PAR *vs. Kdm1b*^{KD}	>0,9999	*Kdm1b*^{KD} *n*=4
**B16.F10**		
Klf4		CTR *n*=4
PAR *vs. Kdm1b*^{OVER}	>0,9999	*Kdm1b*^{OVER} *n*=4
PAR *vs. Kdm1b*^{KD}	>0,9999	*Kdm1b*^{KD} *n*=4
Myc		CTR *n*=4
PAR *vs. Kdm1b*^{OVER}	0,1233	*Kdm1b*^{OVER} *n*=4
PAR *vs. Kdm1b*^{KD}	>0,9999	*Kdm1b*^{KD} *n*=4
Oct3/4		CTR *n*=6
PAR *vs. Kdm1b*^{OVER}	0,0244	*Kdm1b*^{OVER} *n*=4
PAR *vs. Kdm1b*^{KD}	>0,9999	*Kdm1b*^{KD} *n*=4
Sox2		CTR *n*=4
PAR *vs. Kdm1b*^{OVER}	<0,0001	*Kdm1b*^{OVER} *n*=4
PAR *vs. Kdm1b*^{KD}	>0,9999	*Kdm1b*^{KD} *n*=4
Nanog		CTR *n*=4
PAR *vs. Kdm1b*^{OVER}	<0,0001	*Kdm1b*^{OVER} *n*=4
PAR *vs. Kdm1b*^{KD}	>0,9999	*Kdm1b*^{KD} *n*=4
Hes1		CTR *n*=4
PAR *vs. Kdm1b*^{OVER}	0,1664	*Kdm1b*^{OVER} *n*=4
PAR *vs. Kdm1b*^{KD}	>0,9999	*Kdm1b*^{KD} *n*=4
Nes		CTR *n*=4
PAR *vs. Kdm1b*^{OVER}	0,0870	*Kdm1b*^{OVER} *n*=4
PAR *vs. Kdm1b*^{KD}	>0,9999	*Kdm1b*^{KD} *n*=4

Supplementary Table 2. Correlation and *P* values for clinical studies.

Heatmap row	Heatmap column	Correlation value	*P* value
**Figure 7a**			
**GSE6861**			
IFN-I S[31646105]	IFN-I S[19001271]	0.9275707384	0
IFN-I S[31646105]	Viral mimicry	0.8606136416	0
IFN-I S[19001271]	Viral mimicry	0.8045606165	0
IFN-I S[31646105]	Yamanaka	-0.02001092708	0.8008717841
IFN-I S[19001271]	Yamanaka	-0.02483321831	0.7542680217
Viral mimicry	Yamanaka	0.1164251208	0.1412389192
IFN-I S[31646105]	CSC S[21169407]	0.01150218542	0.884743057
IFN-I S[19001271]	CSC S[21169407]	0.03271509087	0.6800412637
Viral mimicry	CSC S[21169407]	0.126299747	0.1103309118
Yamanaka	CSC S[21169407]	0.4261329653	2.42E-08
IFN-I S[31646105]	CSC S[22909066]	0.3855129975	5.48E-07
IFN-I S[19001271]	CSC S[22909066]	0.4383080285	8.74E-09
Viral mimicry	CSC S[22909066]	0.3453904992	8.24E-06
Yamanaka	CSC S[22909066]	0.07617322291	0.3364887294
CSC S[21169407]	CSC S[22909066]	0.6205745342	0
IFN-I S[31646105]	*KDM1B*	0.04432370336	0.5766415502
IFN-I S[19001271]	*KDM1B*	-0.003091214553	0.9689560951
Viral mimicry	*KDM1B*	0.1378307869	0.08123215066
Yamanaka	*KDM1B*	0.2203417733	0.004975077377
CSC S[21169407]	*KDM1B*	0.3050381765	8.34E-05
CSC S[22909066]	*KDM1B*	0.2436337155	0.00184354536
IFN-I S[31646105]	*IFNB1*	-0.08768691051	0.2683797889
IFN-I S[19001271]	*IFNB1*	-0.09254083276	0.2427002209
Viral mimicry	*IFNB1*	0.2257275132	0.004062215337
Yamanaka	*IFNB1*	0.1269870025	0.108394872
CSC S[21169407]	*IFNB1*	0.101521164	0.1998207826
CSC S[22909066]	*IFNB1*	-0.05536289395	0.485063774
KDM1B	*IFNB1*	0.09177744118	0.2469061098
IFN-I S[31646105]	*MX1*	0.8426320182	1.35E-44
IFN-I S[19001271]	*MX1*	0.8840988642	2.08E-54
Viral mimicry	*MX1*	0.8558221588	2.23E-47
Yamanaka	*MX1*	0.0552205941	0.486590179
CSC S[21169407]	*MX1*	0.1557756468	0.04846902464
CSC S[22909066]	*MX1*	0.3854226948	4.45E-07
KDM1B	*MX1*	0.03719812112	0.6394444074
IFNB1	*MX1*	0.02742841861	0.7298080843
IFN-I S[31646105]	*CXCL10*	0.6754807018	8.52E-23
IFN-I S[19001271]	*CXCL10*	0.6289097574	4.14E-19
Viral mimicry	*CXCL10*	0.7420654633	2.03E-29
Yamanaka	*CXCL10*	0.09690598179	0.2213680056
CSC S[21169407]	*CXCL10*	0.2206120749	0.004920758363
CSC S[22909066]	*CXCL10*	0.4789973538	1.30E-10
KDM1B	*CXCL10*	0.2669847984	0.0006179234885

IFNB1	*CXCL10*	0.119618501	0.1306880301
MX1	*CXCL10*	0.586321869	3.04E-16
IFN-I S[31646105]	*DHX58*	0.7291867955	0
IFN-I S[19001271]	*DHX58*	0.7010495744	0
Viral mimicry	*DHX58*	0.7207902001	0
Yamanaka	*DHX58*	-0.05340464688	0.5006558911
CSC S[21169407]	*DHX58*	-0.1015154129	0.1998463598
CSC S[22909066]	*DHX58*	0.1632763975	0.03859789153
KDM1B	*DHX58*	-0.04608785232	0.5615496359
IFNB1	*DHX58*	-0.05900908673	0.4567412707
MX1	*DHX58*	0.729039229	5.71E-28
CXCL10	*DHX58*	0.4184354285	3.32E-08
IFN-I S[31646105]	*OASL*	0.7993530021	0
IFN-I S[19001271]	*OASL*	0.8007965263	0
Viral mimicry	*OASL*	0.8114072924	0
Yamanaka	*OASL*	0.0837962963	0.2902568713
CSC S[21169407]	*OASL*	0.04826029446	0.5428404217
CSC S[22909066]	*OASL*	0.2722969864	0.0004971140216
KDM1B	*OASL*	0.1085404507	0.1705166967
IFNB1	*OASL*	-0.0004428341385	0.995561098
MX1	*OASL*	0.8113776824	6.48E-39
CXCL10	*OASL*	0.6175412767	2.66E-18
DHX58	*OASL*	0.6367092248	0
IFN-I S[31646105]	*STAT1*	0.8601449275	0
IFN-I S[19001271]	*STAT1*	0.7649327122	0
Viral mimicry	*STAT1*	0.8112836439	0
Yamanaka	*STAT1*	0.05263112491	0.5068867858
CSC S[21169407]	*STAT1*	0.1015786749	0.1995651413
CSC S[22909066]	*STAT1*	0.3594116632	3.32E-06
KDM1B	*STAT1*	0.2343744496	0.002767006094
IFNB1	*STAT1*	0.01390326662	0.8609026227
MX1	*STAT1*	0.7105983369	4.69E-26
CXCL10	*STAT1*	0.7216591339	3.48E-27
DHX58	*STAT1*	0.5604267311	0
OASL	*STAT1*	0.6740338164	0
IFN-I S[31646105]	*CD274*	0.6651656315	0
IFN-I S[19001271]	*CD274*	0.5352800782	0
Viral mimicry	*CD274*	0.6904675638	0
Yamanaka	*CD274*	0.1514435243	0.05521752792
CSC S[21169407]	*CD274*	0.1892023234	0.01634302578
CSC S[22909066]	*CD274*	0.3958333333	2.57E-07
KDM1B	*CD274*	0.3213281583	3.24E-05
IFNB1	*CD274*	0.09049344375	0.2533146448
MX1	*CD274*	0.5120862895	3.83E-12
CXCL10	*CD274*	0.7748222731	1.75E-33
DHX58	*CD274*	0.3357832988	1.50E-05
OASL	*CD274*	0.5900592363	0

STAT1	*CD274*	0.7596819646	0
IFN-I S[31646105]	*PDCD1LG2*	0.2556964573	0.001097756022
"19001271"	*PDCD1LG2*	0.1441741431	0.06809496538
Viral mimicry	*PDCD1LG2*	0.2617753623	0.0008260175685
Yamanaka	*PDCD1LG2*	0.2321399816	0.003110072419
CSC S[21169407]	*PDCD1LG2*	0.1265240396	0.1096961074
CSC S[22909066]	*PDCD1LG2*	0.1134776858	0.1516402774
KDM1B	*PDCD1LG2*	0.1662268276	0.03508014205
IFNB1	*PDCD1LG2*	0.02309351277	0.7709884384
MX1	*PDCD1LG2*	0.1169169232	0.139670113
CXCL10	*PDCD1LG2*	0.2719880607	0.0004825024358
DHX58	*PDCD1LG2*	0.05266850702	0.5065847369
OASL	*PDCD1LG2*	0.2245916724	0.004255946772
STAT1	*PDCD1LG2*	0.3265125374	2.62E-05
CD274	*PDCD1LG2*	0.3925810904	3.27E-07
IFN-I S[31646105]	*KLF4*	-0.1151081205	0.1458172054
IFN-I S[19001271]	*KLF4*	-0.1482286634	0.06063971519
Viral mimicry	*KLF4*	-0.05571658615	0.4822756834
Yamanaka	*KLF4*	0.5304549114	0
CSC S[21169407]	*KLF4*	-0.08782781228	0.267609179
CSC S[22909066]	*KLF4*	-0.2982027835	0.0001306591408
KDM1B	*KLF4*	-0.08165551074	0.3031321185
IFNB1	*KLF4*	0.1180699333	0.1356760835
MX1	*KLF4*	-0.1512912291	0.05539629023
CXCL10	*KLF4*	-0.1263285932	0.110300361
DHX58	*KLF4*	-0.1330831608	0.09236840581
OASL	*KLF4*	-0.02001380262	0.8008437651
STAT1	*KLF4*	-0.1073729009	0.1750105156
CD274	*KLF4*	-0.01653151599	0.8349509045
PDCD1LG2	*KLF4*	0.2277864044	0.003731193619
IFN-I S[31646105]	*MYC*	0.09034404524	0.254395583
IFN-I S[19001271]	*MYC*	0.129674387	0.1011106626
Viral mimicry	*MYC*	0.1793624617	0.02280916405
Yamanaka	*MYC*	0.5710915158	2.57E-15
CSC S[21169407]	*MYC*	0.5958902632	7.52E-17
CSC S[22909066]	*MYC*	0.4742458509	2.09E-10
KDM1B	*MYC*	0.2972588076	0.0001284926567
IFNB1	*MYC*	0.04679527087	0.5555514303
MX1	*MYC*	0.2074193569	0.008287527216
CXCL10	*MYC*	0.2227518032	0.004509174482
DHX58	*MYC*	0.07267234971	0.3595956326
OASL	*MYC*	0.08284748512	0.2961047052
STAT1	*MYC*	0.1282668051	0.1049003321
CD274	*MYC*	0.2034351326	0.009644701931
PDCD1LG2	*MYC*	0.1131140918	0.1531011124
KLF4	*MYC*	-0.1695295168	0.03156198922
IFN-I S[31646105]	*POU5F1*	0.007194622149	0.9278268197

IFN-I S[19001271]	*POU5F1*	-0.02310215601	0.7711367689
Viral mimicry	*POU5F1*	0.1165359131	0.1409738569
Yamanaka	*POU5F1*	0.6294676138	3.77E-19
CSC S[21169407]	*POU5F1*	0.2343672607	0.002767862243
CSC S[22909066]	*POU5F1*	-0.07357665745	0.3536323503
KDM1B	*POU5F1*	0.2449523306	0.001737792066
IFNB1	*POU5F1*	0.0558115193	0.4819316103
MX1	*POU5F1*	0.06963720617	0.3800639689
CXCL10	*POU5F1*	0.1248276467	0.1146296675
DHX58	*POU5F1*	0.01873707351	0.813498602
OASL	*POU5F1*	0.0962532323	0.224510049
STAT1	*POU5F1*	0.0931088201	0.2400879834
CD274	*POU5F1*	0.202832559	0.009866199545
PDCD1LG2	*POU5F1*	0.04464720256	0.5738599472
KLF4	*POU5F1*	0.1498477038	0.05779286975
MYC	*POU5F1*	0.372113754	1.17E-06
IFN-I S[31646105]	*SOX2*	-0.1207643202	0.1269286002
IFN-I S[19001271]	*SOX2*	-0.1074764205	0.174593159
Viral mimicry	*SOX2*	-0.1161116862	0.1423184377
Yamanaka	*SOX2*	0.07445364619	0.3475343667
CSC S[21169407]	*SOX2*	0.08449217851	0.2862588468
CSC S[22909066]	*SOX2*	0.07047964113	0.3739255433
KDM1B	*SOX2*	-0.07337824461	0.3549354143
IFNB1	*SOX2*	-0.02205831608	0.7809887056
MX1	*SOX2*	-0.121713338	0.1240340292
CXCL10	*SOX2*	-0.1467464248	0.06323093408
DHX58	*SOX2*	-0.1151541293	0.1456553867
OASL	*SOX2*	-0.132720842	0.09326487192
STAT1	*SOX2*	-0.1203818726	0.1281430054
CD274	*SOX2*	-0.1864820566	0.0179733249
PDCD1LG2	*SOX2*	-0.1100845411	0.1643200948
KLF4	*SOX2*	0.06793190706	0.391475262
MYC	*SOX2*	-0.1015457522	0.1999312482
POU5F1	*SOX2*	-0.2827684918	0.0002785919613
IFN-I S[31646105]	*NANOG*	-0.2558936162	0.001051573851
IFN-I S[19001271]	*NANOG*	-0.236256496	0.002550934998
Viral mimicry	*NANOG*	-0.1801142887	0.0222356798
Yamanaka	*NANOG*	-0.06561138832	0.408282487
CSC S[21169407]	*NANOG*	-0.1211626705	0.1257572442
CSC S[22909066]	*NANOG*	-0.06921157495	0.3829899838
KDM1B	*NANOG*	-0.03998884286	0.6145077243
IFNB1	*NANOG*	0.2270389256	0.00377621885
MX1	*NANOG*	-0.198858694	0.01144264432
CXCL10	*NANOG*	-0.1658675033	0.03548217883
DHX58	*NANOG*	-0.1017527187	0.1990112981
OASL	*NANOG*	-0.24629216	0.001636024508
STAT1	*NANOG*	-0.3161076001	4.41E-05

CD274	*NANOG*	-0.2020992941	0.01014178393
PDCD1LG2	*NANOG*	-0.2005076781	0.01076343402
KLF4	*NANOG*	0.06698446269	0.3985220593
MYC	*NANOG*	-0.05487273164	0.4893437901
POU5F1	*NANOG*	-0.1930026354	0.01416877942
SOX2	*NANOG*	0.08974011512	0.2575975122
IFN-I S[31646105]	*HES1*	-0.04348401196	0.5835195527
IFN-I S[19001271]	*HES1*	-0.003787094548	0.9619436903
Viral mimicry	*HES1*	-0.101273867	0.2009227461
Yamanaka	*HES1*	-0.2395416379	0.00226531534
CSC S[21169407]	*HES1*	-0.1274758454	0.1070340912
CSC S[22909066]	*HES1*	-0.01720151829	0.828362485
KDM1B	*HES1*	-0.1511273228	0.0556642142
IFNB1	*HES1*	-0.1658557626	0.03559962806
MX1	*HES1*	-0.06207733931	0.4340438216
CXCL10	*HES1*	-0.1865152595	0.01783624948
DHX58	*HES1*	0.004388083736	0.9559077798
OASL	*HES1*	-0.08854382333	0.2637165667
STAT1	*HES1*	-0.09263285024	0.242230571
CD274	*HES1*	-0.2532752473	0.001227200648
PDCD1LG2	*HES1*	-0.2759661836	0.0004144917316
KLF4	*HES1*	-0.03159362779	0.6904358937
MYC	*HES1*	-0.2133572196	0.006578341864
POU5F1	*HES1*	-0.2649084605	0.0006837838737
SOX2	*HES1*	0.1809782609	0.02170879573
NANOG	*HES1*	0.1007965988	0.2032868918
IFN-I S[31646105]	*NES*	-0.07959512307	0.315183435
IFN-I S[19001271]	*NES*	0.009227628249	0.9074235607
Viral mimicry	*NES*	-0.06446112261	0.4161664009
Yamanaka	*NES*	0.000983436853	0.9901247273
CSC S[21169407]	*NES*	0.1905567058	0.01558053915
CSC S[22909066]	*NES*	0.1890585461	0.01642584896
KDM1B	*NES*	0.03982059453	0.6159990599
IFNB1	*NES*	-0.07376063952	0.3520498756
MX1	*NES*	0.01866087148	0.8142433338
CXCL10	*NES*	-0.07715671523	0.3306369147
DHX58	*NES*	-0.005610190936	0.9436419027
OASL	*NES*	-0.09161490683	0.2474614699
STAT1	*NES*	-0.1006556246	0.2036968999
CD274	*NES*	-0.06946457327	0.380858984
PDCD1LG2	*NES*	-0.1557798482	0.04854527135
KLF4	*NES*	-0.2018403497	0.01034615188
MYC	*NES*	0.2159178973	0.005943729079
POU5F1	*NES*	0.06744167489	0.3953031404
SOX2	*NES*	-0.03925695882	0.6206556203
NANOG	*NES*	0.01691253804	0.8313744025
HES1	*NES*	-0.09551702323	0.2278307108

**GSE16446**			
IFN-I S[31646105]	IFN-I S[19001271]	0.9253698173	0
IFN-I S[31646105]	Viral mimicry	0.8840891729	0
IFN-I S[19001271]	Viral mimicry	0.8680047226	0
IFN-I S[31646105]	Yamanaka	0.2023890548	0.02678989538
IFN-I S[19001271]	Yamanaka	0.1999722203	0.02869130926
Viral mimicry	Yamanaka	0.2409889576	0.008147432072
IFN-I S[31646105]	CSC S[21169407]	0.2165011459	0.01770427586
IFN-I S[19001271]	CSC S[21169407]	0.3179943052	0.0004278410305
Viral mimicry	CSC S[21169407]	0.3192582818	0.0004048759001
Yamanaka	CSC S[21169407]	0.4532050837	2.78E-07
IFN-I S[31646105]	CSC S[22909066]	0.399909716	7.24E-06
IFN-I S[19001271]	CSC S[22909066]	0.4309674283	1.15E-06
Viral mimicry	CSC S[22909066]	0.351281339	9.25E-05
Yamanaka	CSC S[22909066]	0.2638030419	0.003694816136
CSC S[21169407]	CSC S[22909066]	0.7407875547	0
IFN-I S[31646105]	*KDM1B*	-0.003694701021	0.9680344492
IFN-I S[19001271]	*KDM1B*	0.067393569	0.4640205356
Viral mimicry	*KDM1B*	0.06585874019	0.4742767827
Yamanaka	*KDM1B*	0.3098062365	0.0006082603827
CSC S[21169407]	*KDM1B*	0.3540870894	8.07E-05
CSC S[22909066]	*KDM1B*	0.1214042642	0.1862779801
IFN-I S[31646105]	*IFNB1*	0.2908054219	0.001272085689
IFN-I S[19001271]	*IFNB1*	0.304809891	0.0007117813941
Viral mimicry	*IFNB1*	0.4835656705	2.21E-08
Yamanaka	*IFNB1*	0.09995850399	0.2773672846
CSC S[21169407]	*IFNB1*	0.03742627513	0.6848636443
CSC S[22909066]	*IFNB1*	-0.07889797711	0.3916803828
KDM1B	*IFNB1*	0.08564498515	0.3523275163
IFN-I S[31646105]	*MX1*	0.7746510174	0
IFN-I S[19001271]	*MX1*	0.8559899993	0
Viral mimicry	*MX1*	0.8194874644	0
Yamanaka	*MX1*	0.1700743107	0.06336810607
CSC S[21169407]	*MX1*	0.3937356761	1.02E-05
CSC S[22909066]	*MX1*	0.3678102646	4.06E-05
KDM1B	*MX1*	0.06954649628	0.4498423597
IFNB1	*MX1*	0.3390032971	0.0001521626024
IFN-I S[31646105]	*CXCL10*	0.7715952497	0
IFN-I S[19001271]	*CXCL10*	0.686165706	0
Viral mimicry	*CXCL10*	0.6855267727	0
Yamanaka	*CXCL10*	0.2606153205	0.004143105671
CSC S[21169407]	*CXCL10*	0.3096603931	0.0006120313571
CSC S[22909066]	*CXCL10*	0.5215362178	1.56E-09
KDM1B	*CXCL10*	0.02189735398	0.812105661
IFNB1	*CXCL10*	0.1024413196	0.2655476054
MX1	*CXCL10*	0.5148482534	2.85E-09
IFN-I S[31646105]	*DHX58*	0.63886381	0

IFN-I S[19001271]	*DHX58*	0.5819154108	0
Viral mimicry	*DHX58*	0.6698659629	0
Yamanaka	*DHX58*	0.0794499618	0.3878440999
CSC S[21169407]	*DHX58*	0.1168831169	0.2033065993
CSC S[22909066]	*DHX58*	0.2065837905	0.02374512319
KDM1B	*DHX58*	-0.1278561011	0.1638198054
IFNB1	*DHX58*	0.280183138	0.001938777172
MX1	*DHX58*	0.5892631433	0
CXCL10	*DHX58*	0.4061948746	5.06E-06
IFN-I S[31646105]	*OASL*	0.7834849642	0
IFN-I S[19001271]	*OASL*	0.8698659629	0
Viral mimicry	*OASL*	0.8148274186	0
Yamanaka	*OASL*	0.2036877561	0.02581341448
CSC S[21169407]	*OASL*	0.39857629	7.81E-06
CSC S[22909066]	*OASL*	0.3605458712	5.86E-05
KDM1B	*OASL*	0.1444961456	0.1152744655
IFNB1	*OASL*	0.3212798133	0.0003463030832
MX1	*OASL*	0.8499965275	0
CXCL10	*OASL*	0.4893395375	2.20E-08
DHX58	*OASL*	0.5587193555	0
IFN-I S[31646105]	*STAT1*	0.8515938607	0
IFN-I S[19001271]	*STAT1*	0.7624696159	0
Viral mimicry	*STAT1*	0.7518230433	0
Yamanaka	*STAT1*	0.2149871519	0.01852989909
CSC S[21169407]	*STAT1*	0.2571359122	0.004687659994
CSC S[22909066]	*STAT1*	0.4692478644	9.36E-08
KDM1B	*STAT1*	-0.08310993819	0.366304451
IFNB1	*STAT1*	0.1547262913	0.09153063007
MX1	*STAT1*	0.6147649142	0
CXCL10	*STAT1*	0.7954094034	0
DHX58	*STAT1*	0.5318285992	5.51E-10
OASL	*STAT1*	0.6366553233	0
IFN-I S[31646105]	*CD274*	0.7231625862	1.09E-20
IFN-I S[19001271]	*CD274*	0.5610954946	2.63E-11
Viral mimicry	*CD274*	0.5603766935	2.83E-11
Yamanaka	*CD274*	0.2212831122	0.01514666918
CSC S[21169407]	*CD274*	0.1200536844	0.191523237
CSC S[22909066]	*CD274*	0.4063900382	4.11E-06
KDM1B	*CD274*	-0.1250019533	0.173723239
IFNB1	*CD274*	0.0717482872	0.4361323902
MX1	*CD274*	0.3713042074	2.98E-05
CXCL10	*CD274*	0.797508156	1.16E-27
DHX58	*CD274*	0.4181999066	2.01E-06
OASL	*CD274*	0.3892499292	1.11E-05
STAT1	*CD274*	0.8339413049	3.00E-32
IFN-I S[31646105]	*PDCD1LG2*	0.5385244436	2.24E-10
"19001271"	*PDCD1LG2*	0.4660158102	8.14E-08

Viral mimicry	*PDCD1LG2*	0.4698806686	6.15E-08
Yamanaka	*PDCD1LG2*	0.03456843282	0.7077893747
CSC S[21169407]	*PDCD1LG2*	0.0205813261	0.8234413998
CSC S[22909066]	*PDCD1LG2*	0.2467883999	0.006581725951
KDM1B	*PDCD1LG2*	0.1142754953	0.2139421693
IFNB1	*PDCD1LG2*	0.09017671427	0.32733588
MX1	*PDCD1LG2*	0.2043617688	0.02516090594
CXCL10	*PDCD1LG2*	0.5549526963	4.79E-11
DHX58	*PDCD1LG2*	0.1973959952	0.03069208892
OASL	*PDCD1LG2*	0.3311624612	0.0002202809949
STAT1	*PDCD1LG2*	0.5510357508	6.97E-11
CD274	*PDCD1LG2*	0.5520402529	6.33E-11
IFN-I S[31646105]	*KLF4*	0.003340509758	0.9711002993
IFN-I S[19001271]	*KLF4*	0.01191054934	0.8971427559
Viral mimicry	*KLF4*	0.04125286478	0.6541790725
Yamanaka	*KLF4*	0.4645183693	1.30E-07
CSC S[21169407]	*KLF4*	0.08618654073	0.3487819769
CSC S[22909066]	*KLF4*	0.04647544968	0.6137523199
KDM1B	*KLF4*	0.1028890895	0.2630500741
IFNB1	*KLF4*	0.09941679877	0.2799924234
MX1	*KLF4*	-0.03925272588	0.6699403406
CXCL10	*KLF4*	0.02012639767	0.8270555774
DHX58	*KLF4*	-0.02231404959	0.8085976571
OASL	*KLF4*	0.01710535454	0.8526993919
STAT1	*KLF4*	0.02290436836	0.8036345163
CD274	*KLF4*	0.001312593431	0.9886478866
PDCD1LG2	*KLF4*	-0.1301620428	0.1564931244
IFN-I S[31646105]	*MYC*	0.2083377463	0.02240360103
IFN-I S[19001271]	*MYC*	0.2355723237	0.009594407671
Viral mimicry	*MYC*	0.2851105721	0.001597740822
Yamanaka	*MYC*	0.543809542	1.37E-10
CSC S[21169407]	*MYC*	0.5406495949	1.84E-10
CSC S[22909066]	*MYC*	0.3554141875	6.79E-05
KDM1B	*MYC*	0.3254294142	0.000286924342
IFNB1	*MYC*	0.1648661882	0.07194756786
MX1	*MYC*	0.279509479	0.001990208146
CXCL10	*MYC*	0.2287836461	0.01195749646
DHX58	*MYC*	0.1653798274	0.07105366954
OASL	*MYC*	0.2941112128	0.001112035545
STAT1	*MYC*	0.1583723841	0.08405194616
CD274	*MYC*	0.1007156772	0.2737257925
PDCD1LG2	*MYC*	0.1309296858	0.1540432513
KLF4	*MYC*	-0.09972584854	0.2784927132
IFN-I S[31646105]	*POU5F1*	0.1245294812	0.1751331031
IFN-I S[19001271]	*POU5F1*	0.08440169456	0.358882176
Viral mimicry	*POU5F1*	0.06893534273	0.4538422228
Yamanaka	*POU5F1*	0.5326758803	5.00E-10

CSC S[21169407]	*POU5F1*	0.1694978818	0.06427940983
CSC S[22909066]	*POU5F1*	0.05286478228	0.5658413392
KDM1B	*POU5F1*	0.1613098132	0.07841959384
IFNB1	*POU5F1*	-0.06763675887	0.4629429439
MX1	*POU5F1*	-0.0349190916	0.7045798219
CXCL10	*POU5F1*	0.2018751302	0.02718494139
DHX58	*POU5F1*	0.01769567331	0.8476755068
OASL	*POU5F1*	0.05173970415	0.5741476074
STAT1	*POU5F1*	0.2177790124	0.01703245012
CD274	*POU5F1*	0.2301934686	0.01142861689
PDCD1LG2	*POU5F1*	0.1175708688	0.2009378414
KLF4	*POU5F1*	-0.03586360164	0.696975274
MYC	*POU5F1*	0.201441422	0.02736640288
IFN-I S[31646105]	*SOX2*	-0.09322538583	0.3111789711
IFN-I S[19001271]	*SOX2*	-0.08642281829	0.3479552317
Viral mimicry	*SOX2*	-0.103931009	0.2586225715
Yamanaka	*SOX2*	0.135408944	0.1403186672
CSC S[21169407]	*SOX2*	-0.05727838266	0.5343389685
CSC S[22909066]	*SOX2*	-0.1012155379	0.2713395561
KDM1B	*SOX2*	-0.1903017402	0.03735193119
IFNB1	*SOX2*	0.03504769445	0.7039257443
MX1	*SOX2*	0.0019515278	0.9831226706
CXCL10	*SOX2*	-0.07298088926	0.4282704452
DHX58	*SOX2*	-0.1091848552	0.2351906169
OASL	*SOX2*	-0.06606373023	0.473435321
STAT1	*SOX2*	-0.09125302322	0.3215714746
CD274	*SOX2*	0.09210741061	0.3170424976
PDCD1LG2	*SOX2*	-0.2381076398	0.008823566538
KLF4	*SOX2*	0.1007189748	0.2737100041
MYC	*SOX2*	-0.26527976	0.003408968094
POU5F1	*SOX2*	-0.1554242576	0.09005954045
IFN-I S[31646105]	*NANOG*	-0.1026323054	0.264652803
IFN-I S[19001271]	*NANOG*	-0.08608251629	0.3498638987
Viral mimicry	*NANOG*	-0.1388744685	0.1303532334
Yamanaka	*NANOG*	0.1021808844	0.266771102
CSC S[21169407]	*NANOG*	-0.04562130291	0.6207531889
CSC S[22909066]	*NANOG*	-0.1445380661	0.1152460307
KDM1B	*NANOG*	0.0927184053	0.3138291616
IFNB1	*NANOG*	0.02787008775	0.7625265502
MX1	*NANOG*	0.01243838537	0.8927425587
CXCL10	*NANOG*	-0.1396939713	0.1280779247
DHX58	*NANOG*	-0.03022090114	0.7431663822
OASL	*NANOG*	0.02869301461	0.7557319258
STAT1	*NANOG*	-0.0448052726	0.6270210723
CD274	*NANOG*	-0.02990843082	0.7457308145
PDCD1LG2	*NANOG*	-0.2168422003	0.01736429434
KLF4	*NANOG*	-0.001079937982	0.9906599332

MYC	*NANOG*	0.07566523948	0.4114333789
POU5F1	*NANOG*	0.2020595216	0.0268863435
SOX2	*NANOG*	0.06618885405	0.4725960244
IFN-I S[31646105]	*HES1*	-0.2826724078	0.001825146008
IFN-I S[19001271]	*HES1*	-0.1649836794	0.07179246904
Viral mimicry	*HES1*	-0.1609347871	0.07912295369
Yamanaka	*HES1*	-0.1246961595	0.1745528614
CSC S[21169407]	*HES1*	-0.04607958886	0.616778176
CSC S[22909066]	*HES1*	-0.267303285	0.003253270645
KDM1B	*HES1*	0.07853323147	0.3933566069
IFNB1	*HES1*	0.08082519763	0.3801797164
MX1	*HES1*	-0.1503993333	0.1010437469
CXCL10	*HES1*	-0.3666921314	4.30E-05
DHX58	*HES1*	-0.3020695882	0.0008407708655
OASL	*HES1*	-0.04770470172	0.6043978645
STAT1	*HES1*	-0.3075005209	0.0006704578124
CD274	*HES1*	-0.394100969	8.44E-06
PDCD1LG2	*HES1*	-0.1072437448	0.2436687598
KLF4	*HES1*	0.05010070144	0.5863494051
MYC	*HES1*	-0.05274333763	0.5672371289
POU5F1	*HES1*	-0.1327800542	0.1480881944
SOX2	*HES1*	-0.1037296058	0.2595515299
NANOG	*HES1*	0.05754576281	0.5324294407
IFN-I S[31646105]	*NES*	-0.1252934232	0.1724853332
IFN-I S[19001271]	*NES*	-0.1297451212	0.1576432559
Viral mimicry	*NES*	-0.1225710119	0.1820580284
Yamanaka	*NES*	0.04525314258	0.6231160852
CSC S[21169407]	*NES*	0.0007708868671	0.9933526073
CSC S[22909066]	*NES*	0.02241127856	0.807779668
KDM1B	*NES*	0.1412042503	0.1238621747
IFNB1	*NES*	-0.04748949144	0.6065062372
MX1	*NES*	-0.1388082506	0.1304176326
CXCL10	*NES*	-0.07224112786	0.4324445142
DHX58	*NES*	0.08420723661	0.3599934809
OASL	*NES*	-0.1198555455	0.1919894424
STAT1	*NES*	-0.1617265088	0.07764400217
CD274	*NES*	-0.09803128348	0.2867823455
PDCD1LG2	*NES*	-0.05065291106	0.5827173304
KLF4	*NES*	-0.07792207792	0.3970575858
MYC	*NES*	0.2713839006	0.00271558153
POU5F1	*NES*	-0.02422390444	0.7925690276
SOX2	*NES*	-0.178078648	0.05166450322
NANOG	*NES*	-0.002875204659	0.9751366114
HES1	*NES*	-0.1039377735	0.2581958418
**GSE32646**			
IFN-I S[31646105]	IFN-I S[19001271]	0.9250374813	0
IFN-I S[31646105]	Viral mimicry	0.8383808096	0

IFN-I S[19001271]	Viral mimicry	0.8375364949	0
IFN-I S[31646105]	Yamanaka	-0.09135958337	0.3310191161
IFN-I S[19001271]	Yamanaka	-0.0652173913	0.4880831738
Viral mimicry	Yamanaka	0.002635524343	0.9776964573
IFN-I S[31646105]	CSC S[21169407]	0.0803519293	0.392755804
IFN-I S[19001271]	CSC S[21169407]	0.05366527263	0.5684142274
Viral mimicry	CSC S[21169407]	0.1101317762	0.2409356736
Yamanaka	CSC S[21169407]	0.2546516216	0.006150192973
IFN-I S[31646105]	CSC S[22909066]	0.2463820721	0.008085239283
IFN-I S[19001271]	CSC S[22909066]	0.1628264815	0.08211465198
Viral mimicry	CSC S[22909066]	0.1034167127	0.2709628972
Yamanaka	CSC S[22909066]	0.121944291	0.1939055207
CSC S[21169407]	CSC S[22909066]	0.7150161761	0
IFN-I S[31646105]	*KDM1B*	0.01377732187	0.8836619582
IFN-I S[19001271]	*KDM1B*	0.09940819064	0.2900496886
Viral mimicry	*KDM1B*	0.159401878	0.08882728733
Yamanaka	*KDM1B*	0.09325337331	0.3210610364
CSC S[21169407]	*KDM1B*	0.3773534285	3.68E-05
CSC S[22909066]	*KDM1B*	0.2712380652	0.003463792866
IFN-I S[31646105]	*IFNB1*	0.2199163576	0.01836443542
IFN-I S[19001271]	*IFNB1*	0.238554407	0.01039522951
Viral mimicry	*IFNB1*	0.3738735895	4.39E-05
Yamanaka	*IFNB1*	0.0927089087	0.3239039352
CSC S[21169407]	*IFNB1*	-0.005389410558	0.954375075
CSC S[22909066]	*IFNB1*	-0.01628659355	0.8626595608
KDM1B	*IFNB1*	-0.09855598517	0.2942202446
IFN-I S[31646105]	*MX1*	0.7786948631	0
IFN-I S[19001271]	*MX1*	0.8532865146	0
Viral mimicry	*MX1*	0.8404403062	0
Yamanaka	*MX1*	0.01193087667	0.8991692707
CSC S[21169407]	*MX1*	0.1414897814	0.1313417779
CSC S[22909066]	*MX1*	0.1070701491	0.2543253455
KDM1B	*MX1*	0.07902627634	0.4006302015
IFNB1	*MX1*	0.3130434783	0.0006967484289
IFN-I S[31646105]	*CXCL10*	0.7252899866	0
IFN-I S[19001271]	*CXCL10*	0.6250769352	0
Viral mimicry	*CXCL10*	0.6257792157	0
Yamanaka	*CXCL10*	-0.001546595123	0.9869244028
CSC S[21169407]	*CXCL10*	0.4183303085	4.06E-06
CSC S[22909066]	*CXCL10*	0.5884794445	0
KDM1B	*CXCL10*	0.2323206818	0.01263204934
IFNB1	*CXCL10*	0.1500591809	0.1093797754
MX1	*CXCL10*	0.5036139825	1.54E-08
IFN-I S[31646105]	*DHX58*	0.3924406218	1.69E-05
IFN-I S[19001271]	*DHX58*	0.3953523238	1.45E-05
Viral mimicry	*DHX58*	0.6075041427	0
Yamanaka	*DHX58*	-0.0598674347	0.5245166898

CSC S[21169407]	*DHX58*	-0.08190641521	0.3836414036
CSC S[22909066]	*DHX58*	-0.2024382546	0.03018856801
KDM1B	*DHX58*	0.1269312712	0.1761953681
IFNB1	*DHX58*	0.0823798627	0.3808911183
MX1	*DHX58*	0.4549277993	4.46E-07
CXCL10	*DHX58*	0.1240037876	0.1864409825
IFN-I S[31646105]	*OASL*	0.8396354454	0
IFN-I S[19001271]	*OASL*	0.8411189142	0
Viral mimicry	*OASL*	0.7719876904	0
Yamanaka	*OASL*	-0.02450879823	0.7945859197
CSC S[21169407]	*OASL*	0.1805807623	0.0535571459
CSC S[22909066]	*OASL*	0.2176911544	0.01960221607
KDM1B	*OASL*	0.09475262369	0.3133164237
IFNB1	*OASL*	0.2619821668	0.004792105898
MX1	*OASL*	0.7525605618	0
CXCL10	*OASL*	0.6328572556	0
DHX58	*OASL*	0.393150793	1.63E-05
IFN-I S[31646105]	*STAT1*	0.8677818985	0
IFN-I S[19001271]	*STAT1*	0.7789473684	0
Viral mimicry	*STAT1*	0.7505563008	0
Yamanaka	*STAT1*	-0.001333543754	0.9887301113
CSC S[21169407]	*STAT1*	0.2778742208	0.002726389941
CSC S[22909066]	*STAT1*	0.3516294484	0.0001283642192
KDM1B	*STAT1*	0.1146768721	0.2219777715
IFNB1	*STAT1*	0.2084273653	0.02556100391
MX1	*STAT1*	0.686490965	0
CXCL10	*STAT1*	0.8309713564	0
DHX58	*STAT1*	0.298484968	0.001250166947
OASL	*STAT1*	0.7876114574	0
IFN-I S[31646105]	*CD274*	0.7327467845	0
IFN-I S[19001271]	*CD274*	0.5923459323	0
Viral mimicry	*CD274*	0.5815828928	0
Yamanaka	*CD274*	0.1052947211	0.26232054
CSC S[21169407]	*CD274*	0.2135721613	0.02208446079
CSC S[22909066]	*CD274*	0.394137142	1.54E-05
KDM1B	*CD274*	0.0664246824	0.4800524919
IFNB1	*CD274*	0.1127673006	0.2298097224
MX1	*CD274*	0.4543912254	4.61E-07
CXCL10	*CD274*	0.8376154028	0
DHX58	*CD274*	0.1759883216	0.06001165932
OASL	*CD274*	0.6070543676	0
STAT1	*CD274*	0.8062021621	0
IFN-I S[31646105]	*PDCD1LG2*	0.5761066835	0
"19001271"	*PDCD1LG2*	0.4517793735	5.44E-07
Viral mimicry	*PDCD1LG2*	0.4462558195	7.69E-07
Yamanaka	*PDCD1LG2*	-0.03354375444	0.7215481685
CSC S[21169407]	*PDCD1LG2*	-0.03150793025	0.7378139429

CSC S[22909066]	*PDCD1LG2*	0.2728556774	0.003269142237
KDM1B	*PDCD1LG2*	0.01492148663	0.8740744424
IFNB1	*PDCD1LG2*	0.08097530182	0.3890852933
MX1	*PDCD1LG2*	0.2875404403	0.001904453171
CXCL10	*PDCD1LG2*	0.6299613351	0
DHX58	*PDCD1LG2*	0.07204292591	0.4436365022
OASL	*PDCD1LG2*	0.4283200505	2.28E-06
STAT1	*PDCD1LG2*	0.5472737316	2.44E-10
CD274	*PDCD1LG2*	0.7429653594	0
IFN-I S[31646105]	*KLF4*	-0.2575209403	0.005461738184
IFN-I S[19001271]	*KLF4*	-0.1986593492	0.03330616809
Viral mimicry	*KLF4*	-0.2152064042	0.02090178855
Yamanaka	*KLF4*	0.3416686723	0.0001861741495
CSC S[21169407]	*KLF4*	-0.07951187372	0.3982820071
CSC S[22909066]	*KLF4*	-0.1189304779	0.2055299341
KDM1B	*KLF4*	0.1334535369	0.1550656742
IFNB1	*KLF4*	-0.02706946686	0.7739828236
MX1	*KLF4*	-0.2167766779	0.01996430509
CXCL10	*KLF4*	-0.2909070106	0.001610180719
DHX58	*KLF4*	-0.1755116212	0.06063022717
OASL	*KLF4*	-0.255820468	0.005789442138
STAT1	*KLF4*	-0.3051617816	0.0009124492251
CD274	*KLF4*	-0.3010940626	0.00107613566
PDCD1LG2	*KLF4*	-0.2217912956	0.01720892216
IFN-I S[31646105]	*MYC*	0.009595202399	0.9188393531
IFN-I S[19001271]	*MYC*	0.007740866409	0.9344914909
Viral mimicry	*MYC*	0.08236408112	0.380982601
Yamanaka	*MYC*	0.5314605855	1.51E-09
CSC S[21169407]	*MYC*	0.3155290776	0.0006287574594
CSC S[22909066]	*MYC*	0.1846524106	0.04832492455
KDM1B	*MYC*	0.1489623609	0.1120187768
IFNB1	*MYC*	-0.07822141561	0.4054565603
MX1	*MYC*	0.03658959994	0.6974455831
CXCL10	*MYC*	0.06232147084	0.5076351071
DHX58	*MYC*	0.09361634972	0.3191747628
OASL	*MYC*	-0.0439122544	0.6407605024
STAT1	*MYC*	-0.01742286751	0.8531796545
CD274	*MYC*	0.09855598517	0.2942202446
PDCD1LG2	*MYC*	0.02673400142	0.7764036621
KLF4	*MYC*	-0.003349654185	0.9716581432
IFN-I S[31646105]	*POU5F1*	0.05056419159	0.5909961704
IFN-I S[19001271]	*POU5F1*	0.06945474631	0.460215027
Viral mimicry	*POU5F1*	0.1502643415	0.1088915951
Yamanaka	*POU5F1*	0.3949893474	1.47E-05
CSC S[21169407]	*POU5F1*	0.09096504379	0.3331183454
CSC S[22909066]	*POU5F1*	0.02097372366	0.8236959462
KDM1B	*POU5F1*	0.01758068334	0.8518646275

IFNB1	*POU5F1*	0.1208316894	0.1980273188
MX1	*POU5F1*	0.1798232463	0.05458046456
CXCL10	*POU5F1*	0.1196243983	0.2025712736
DHX58	*POU5F1*	0.1098161446	0.2422928793
OASL	*POU5F1*	0.2139509193	0.02184543212
STAT1	*POU5F1*	0.1816539099	0.05213463541
CD274	*POU5F1*	0.2651621558	0.004291590757
PDCD1LG2	*POU5F1*	0.1539651227	0.1003752773
KLF4	*POU5F1*	-0.3344209517	0.000259352492
MYC	*POU5F1*	-0.0190957153	0.839261621
IFN-I S[31646105]	*SOX2*	0.0106841527	0.9097718287
IFN-I S[19001271]	*SOX2*	-0.02712464911	0.7735344774
Viral mimicry	*SOX2*	-0.03440786398	0.7150662622
Yamanaka	*SOX2*	0.3038118516	0.0009640577552
CSC S[21169407]	*SOX2*	0.1833310647	0.04985744482
CSC S[22909066]	*SOX2*	0.1977317875	0.03415525951
KDM1B	*SOX2*	-0.1329009171	0.1567979133
IFNB1	*SOX2*	0.1442478976	0.1240380264
MX1	*SOX2*	0.02878566398	0.7600745409
CXCL10	*SOX2*	0.1172771192	0.2119466983
DHX58	*SOX2*	-0.1024266204	0.2760341367
OASL	*SOX2*	0.07600030774	0.4195066078
STAT1	*SOX2*	0.1288608239	0.1699041229
CD274	*SOX2*	0.1213882297	0.1962506076
PDCD1LG2	*SOX2*	0.009697801818	0.9180711879
KLF4	*SOX2*	-0.09074069325	0.334822023
MYC	*SOX2*	-0.2172457533	0.0196913791
POU5F1	*SOX2*	0.189209716	0.04284535695
IFN-I S[31646105]	*NANOG*	-0.1978300324	0.03421764597
IFN-I S[19001271]	*NANOG*	-0.1704174229	0.06868432294
Viral mimicry	*NANOG*	-0.2330939793	0.01233340769
Yamanaka	*NANOG*	0.000441884321	0.9962878506
CSC S[21169407]	*NANOG*	-0.2810621005	0.002425368996
CSC S[22909066]	*NANOG*	-0.3530340093	0.0001202128148
KDM1B	*NANOG*	-0.2177779531	0.01955261527
IFNB1	*NANOG*	-0.05552749941	0.5550528463
MX1	*NANOG*	-0.1601988479	0.08722731645
CXCL10	*NANOG*	-0.3301901681	0.0003372214456
DHX58	*NANOG*	-0.06659827981	0.4789036474
OASL	*NANOG*	-0.1905073779	0.04155168476
STAT1	*NANOG*	-0.2408506273	0.009663832613
CD274	*NANOG*	-0.2381519766	0.01052831453
PDCD1LG2	*NANOG*	-0.1400931113	0.1352214918
KLF4	*NANOG*	-0.001956924	0.9834399494
MYC	*NANOG*	-0.0521186775	0.5796253992
POU5F1	*NANOG*	-0.009319024698	0.9211686975
SOX2	*NANOG*	0.07287160276	0.4389581613

IFN-I S[31646105]	*HES1*	-0.001246745049	0.9894657872
IFN-I S[19001271]	*HES1*	0.05862858045	0.5331457433
Viral mimicry	*HES1*	0.1259054683	0.1797370962
Yamanaka	*HES1*	-0.04023514559	0.668991667
CSC S[21169407]	*HES1*	-0.003803361477	0.9678031358
CSC S[22909066]	*HES1*	-0.07750335359	0.4097913205
KDM1B	*HES1*	0.1040085221	0.2682188723
IFNB1	*HES1*	0.07495462795	0.4253960187
MX1	*HES1*	0.02476130356	0.7925169177
CXCL10	*HES1*	-0.0524895447	0.5769276638
DHX58	*HES1*	3.95E-05	0.9996990138
OASL	*HES1*	-0.06752150241	0.4728189288
STAT1	*HES1*	-0.1108024935	0.2380692324
CD274	*HES1*	-0.1670875089	0.07433774655
PDCD1LG2	*HES1*	-0.09075199242	0.3342554583
KLF4	*HES1*	0.1324237845	0.1583051408
MYC	*HES1*	0.1992030301	0.03297219106
POU5F1	*HES1*	-0.3033851495	0.001030130927
SOX2	*HES1*	-0.2384207339	0.01028899382
NANOG	*HES1*	0.08632525842	0.3584409943
IFN-I S[31646105]	*NES*	-0.06538309792	0.4869766932
IFN-I S[19001271]	*NES*	-0.019561272	0.8353968485
Viral mimicry	*NES*	-0.05402824903	0.5657979835
Yamanaka	*NES*	0.114716326	0.2218179749
CSC S[21169407]	*NES*	-0.1975933086	0.03443635181
CSC S[22909066]	*NES*	-0.2500355086	0.007172200908
KDM1B	*NES*	0.03896472816	0.6788566349
IFNB1	*NES*	-0.2212656829	0.01764732527
MX1	*NES*	-0.04766038034	0.612504428
CXCL10	*NES*	-0.2593229701	0.005250117591
DHX58	*NES*	0.1270180699	0.1758980638
OASL	*NES*	-0.1847392093	0.04821818604
STAT1	*NES*	-0.2285015387	0.01420173826
CD274	*NES*	-0.1305689261	0.1640508452
PDCD1LG2	*NES*	0.1025171625	0.2751701733
KLF4	*NES*	0.1360969624	0.1469774556
MYC	*NES*	0.4456087746	8.01E-07
POU5F1	*NES*	-0.1198374497	0.2017639823
SOX2	*NES*	-0.3439800047	0.0001672100296
NANOG	*NES*	-0.04421210447	0.6384802267
HES1	*NES*	0.122362503	0.1923724271
**Supplementary Figure 7a**			
**GSE20271**			
IFN-I S[31646105]	IFN-I S[19001271]	0.8895491042	0
IFN-I S[31646105]	Viral mimicry	0.7216789779	0
IFN-I S[19001271]	Viral mimicry	0.6927959452	0
IFN-I S[31646105]	Yamanaka	0.04665139601	0.5359841414

IFN-I S[19001271]	Yamanaka	0.117990827	0.1166842666
Viral mimicry	Yamanaka	0.05956726519	0.4292627446
IFN-I S[31646105]	CSC S[21169407]	0.0507814952	0.5004757647
IFN-I S[19001271]	CSC S[21169407]	0.1012895655	0.1783561601
Viral mimicry	CSC S[21169407]	0.2390063505	0.00135094123
Yamanaka	CSC S[21169407]	0.1871003023	0.0124919309
IFN-I S[31646105]	CSC S[22909066]	0.30449002	3.91E-05
IFN-I S[19001271]	CSC S[22909066]	0.3634902211	7.38E-07
Viral mimicry	CSC S[22909066]	0.2661722322	0.0003447625883
Yamanaka	CSC S[22909066]	0.1731886132	0.02089150242
"21169407"	CSC S[22909066]	0.6902829895	0
IFN-I S[31646105]	*IFNB1*	-0.08602675629	0.2535488226
IFN-I S[19001271]	*IFNB1*	-0.08931636821	0.2357784159
Viral mimicry	*IFNB1*	0.1572151499	0.03610125756
Yamanaka	*IFNB1*	0.1016173039	0.1771158324
CSC S[21169407]	*IFNB1*	-0.1715885836	0.022011993
CSC S[22909066]	*IFNB1*	-0.2197848028	0.003200788019
IFN-I S[31646105]	*MX1*	0.7494616609	2.39E-33
IFN-I S[19001271]	*MX1*	0.8227428058	4.82E-45
Viral mimicry	*MX1*	0.7666534032	1.06E-35
Yamanaka	*MX1*	0.07807512916	0.3002508483
CSC S[21169407]	*MX1*	0.2258875148	0.002431264734
CSC S[22909066]	*MX1*	0.2831227498	0.0001284727692
IFNB1	*MX1*	-0.03857425174	0.6092045586
IFN-I S[31646105]	*CXCL10*	0.5673420904	1.50E-16
IFN-I S[19001271]	*CXCL10*	0.570766819	8.95E-17
Viral mimicry	*CXCL10*	0.5492800782	2.03E-15
Yamanaka	*CXCL10*	0.3348339794	4.92E-06
CSC S[21169407]	*CXCL10*	0.4263462593	2.96E-09
CSC S[22909066]	*CXCL10*	0.5839018606	1.18E-17
"3456 (IFNB1)"	*CXCL10*	-0.07229064354	0.3375900777
"4599 (MX1)"	*CXCL10*	0.4447938804	4.96E-10
IFN-I S[31646105]	*DHX58*	0.3089626982	2.97E-05
IFN-I S[19001271]	*DHX58*	0.1861300162	0.01296227409
Viral mimicry	*DHX58*	0.4629998649	1.08E-10
Yamanaka	*DHX58*	-0.1707926875	0.02274936574
CSC S[21169407]	*DHX58*	0.01539158809	0.8382699346
CSC S[22909066]	*DHX58*	-0.1142713971	0.1287278795
IFNB1	*DHX58*	-0.004386503867	0.9536600314
MX1	*DHX58*	0.2586921551	0.0004891847237
"3627 (CXCL10)"	*DHX58*	-0.00249487062	0.9736337027
IFN-I S[31646105]	*OASL*	0.5459168638	3.25E-15
IFN-I S[19001271]	*OASL*	0.4707131511	3.35E-11
Viral mimicry	*OASL*	0.5935620532	2.52E-18
Yamanaka	*OASL*	0.08452039361	0.2619872286
CSC S[21169407]	*OASL*	0.08770468345	0.2443724259
CSC S[22909066]	*OASL*	-0.07549735245	0.3165396395

IFNB1	*OASL*	0.2101557941	0.004868816671
MX1	*OASL*	0.5823596751	1.51E-17
CXCL10	*OASL*	0.2946709131	6.53E-05
DHX58	*OASL*	0.3590097954	8.61E-07
IFN-I S[31646105]	*STAT1*	0.8116787607	5.89E-43
IFN-I S[19001271]	*STAT1*	0.7701128331	3.37E-36
Viral mimicry	*STAT1*	0.7308630391	5.22E-31
Yamanaka	*STAT1*	0.08729387395	0.2465974311
CSC S[21169407]	*STAT1*	0.2901380265	8.55E-05
CSC S[22909066]	*STAT1*	0.4381333261	9.58E-10
IFNB1	*STAT1*	-0.1126299036	0.1344321346
MX1	*STAT1*	0.69589729	4.23E-27
CXCL10	*STAT1*	0.6874547838	3.07E-26
DHX58	*STAT1*	0.2373382656	0.001423306655
OASL	*STAT1*	0.412721633	1.04E-08
IFN-I S[31646105]	*PDCD1LG2*	0.08491075912	0.2597822186
IFN-I S[19001271]	*PDCD1LG2*	-0.04508643215	0.5501107936
Viral mimicry	*PDCD1LG2*	0.02018346086	0.7891526984
Yamanaka	*PDCD1LG2*	-0.005574895098	0.9411258149
CSC S[21169407]	*PDCD1LG2*	0.0489771557	0.5161943135
CSC S[22909066]	*PDCD1LG2*	-0.004587585432	0.9515382975
IFNB1	*PDCD1LG2*	0.006417516245	0.9322470794
MX1	*PDCD1LG2*	0.04833034906	0.5217572775
CXCL10	*PDCD1LG2*	0.02288899942	0.7616874483
DHX58	*PDCD1LG2*	0.04544390634	0.5469497659
OASL	*PDCD1LG2*	0.2145988088	0.00402081918
STAT1	*PDCD1LG2*	0.02263366057	0.7642674279
IFN-I S[31646105]	*KLF4*	0.06925739564	0.3583034692
IFN-I S[19001271]	*KLF4*	0.1101264617	0.1433651721
Viral mimicry	*KLF4*	-0.06043225628	0.4229468649
Yamanaka	*KLF4*	0.384168583	1.20E-07
CSC S[21169407]	*KLF4*	-0.09094521551	0.2273105822
CSC S[22909066]	*KLF4*	0.1290832228	0.08593467659
IFNB1	*KLF4*	-0.09314862415	0.2162011698
MX1	*KLF4*	-0.09133044836	0.2253397135
CXCL10	*KLF4*	0.1179813773	0.1167735487
DHX58	*KLF4*	-0.07355133928	0.3292101552
OASL	*KLF4*	-0.2457064462	0.0009468375237
STAT1	*KLF4*	-0.04435871684	0.5565731797
PDCD1LG2	*KLF4*	-0.2126429964	0.004376207906
IFN-I S[31646105]	*MYC*	0.1847672456	0.01354792813
IFN-I S[19001271]	*MYC*	0.2816086543	0.000140086021
Viral mimicry	*MYC*	0.1947126829	0.009201773784
Yamanaka	*MYC*	0.5062629764	5.63E-13
CSC S[21169407]	*MYC*	0.4317934822	1.77E-09
CSC S[22909066]	*MYC*	0.4540696447	1.94E-10
IFNB1	*MYC*	-0.06823607766	0.3654524953

MX1	*MYC*	0.2684132553	0.0002917070931
CXCL10	*MYC*	0.3207192466	1.27E-05
DHX58	*MYC*	-0.06599650931	0.3814354046
OASL	*MYC*	-0.01566665053	0.8355730621
STAT1	*MYC*	0.2418637385	0.001143710033
PDCD1LG2	*MYC*	-0.1195368792	0.1119952009
KLF4	*MYC*	0.01323718412	0.8607892974
IFN-I S[31646105]	*POU5F1*	-0.1003694434	0.182522011
IFN-I S[19001271]	*POU5F1*	-0.1212199877	0.1069985563
Viral mimicry	*POU5F1*	0.02749680162	0.7156060614
Yamanaka	*POU5F1*	0.5009428919	1.07E-12
CSC S[21169407]	*POU5F1*	0.2762787555	0.0001892333803
CSC S[22909066]	*POU5F1*	0.1132746941	0.1322019209
IFNB1	*POU5F1*	0.07460366603	0.3223185205
MX1	*POU5F1*	-0.03979992053	0.5978705647
CXCL10	*POU5F1*	0.1844643254	0.0137046657
DHX58	*POU5F1*	-0.08388306545	0.2656147124
OASL	*POU5F1*	0.07837110494	0.2984167063
STAT1	*POU5F1*	0.0353718959	0.6392541463
PDCD1LG2	*POU5F1*	0.1136046276	0.1310717863
KLF4	*POU5F1*	-0.2091066647	0.005091113846
MYC	*POU5F1*	0.2009007082	0.007167982879
IFN-I S[31646105]	*SOX2*	-0.1690776644	0.02416529226
IFN-I S[19001271]	*SOX2*	-0.2026482851	0.006754027029
Viral mimicry	*SOX2*	-0.08354567207	0.2672686574
Yamanaka	*SOX2*	0.1271723715	0.09071151787
CSC S[21169407]	*SOX2*	-0.130872651	0.08164895246
CSC S[22909066]	*SOX2*	-0.3340113987	5.91E-06
IFNB1	*SOX2*	0.3148654392	1.86E-05
MX1	*SOX2*	-0.04964210025	0.5105073077
CXCL10	*SOX2*	-0.1456142675	0.05245274345
DHX58	*SOX2*	-0.04000408541	0.5956605117
OASL	*SOX2*	0.3015734201	4.30E-05
STAT1	*SOX2*	-0.1098572923	0.144351914
PDCD1LG2	*SOX2*	0.1600345984	0.03285441739
KLF4	*SOX2*	-0.2794201899	0.000158613416
MYC	*SOX2*	-0.2856387481	0.0001111430671
POU5F1	*SOX2*	-0.01553630466	0.8369219593
IFN-I S[31646105]	*NANOG*	-0.009144322301	0.9035773897
IFN-I S[19001271]	*NANOG*	-0.1060730748	0.1587761045
Viral mimicry	*NANOG*	0.09157408417	0.2240995244
Yamanaka	*NANOG*	-0.03917429802	0.6036439636
CSC S[21169407]	*NANOG*	-0.02449125066	0.745559027
CSC S[22909066]	*NANOG*	-0.1537714345	0.04043105756
IFNB1	*NANOG*	0.0970107253	0.1976740701
MX1	*NANOG*	-0.008217663828	0.9133082013
CXCL10	*NANOG*	-0.05661609891	0.452873159

DHX58	*NANOG*	0.1120394924	0.1364994502
OASL	*NANOG*	0.1770890352	0.01804283023
STAT1	*NANOG*	-0.04670043525	0.5359099361
PDCD1LG2	*NANOG*	0.1685333906	0.02452619385
KLF4	*NANOG*	-0.02719785685	0.7185668143
MYC	*NANOG*	-0.1920867259	0.01020905245
POU5F1	*NANOG*	0.04275123786	0.5709768893
SOX2	*NANOG*	0.1779584064	0.0174760809
IFN-I S[31646105]	*HES1*	-0.2183069147	0.003479647875
IFN-I S[19001271]	*HES1*	-0.08966741105	0.2336911051
Viral mimicry	*HES1*	-0.0412297099	0.5844338649
Yamanaka	*HES1*	0.09988307627	0.1844842732
CSC S[21169407]	*HES1*	0.127604319	0.08961387158
CSC S[22909066]	*HES1*	0.04037219833	0.5922783618
IFNB1	*HES1*	-0.1132384006	0.1323266939
MX1	*HES1*	0.01177430612	0.8760428666
CXCL10	*HES1*	-0.04036796416	0.5926501638
DHX58	*HES1*	-0.1688584989	0.02435159373
OASL	*HES1*	-0.2402399335	0.001237655367
STAT1	*HES1*	-0.10259291	0.1729722365
PDCD1LG2	*HES1*	-0.2272312347	0.002286223524
KLF4	*HES1*	0.2344795375	0.001630774355
MYC	*HES1*	0.09933308757	0.1871032954
POU5F1	*HES1*	0.02448912283	0.7455803745
SOX2	*HES1*	-0.06152485986	0.4142323958
NANOG	*HES1*	-0.06905426216	0.3597183708
IFN-I S[31646105]	*NES*	0.01880996141	0.8031982026
IFN-I S[19001271]	*NES*	0.1928712587	0.009898382552
Viral mimicry	*NES*	-0.004955701371	0.9476549754
Yamanaka	*NES*	0.2465296526	0.0009089364566
CSC S[21169407]	*NES*	0.02441890465	0.7462849504
CSC S[22909066]	*NES*	0.1692928444	0.02387922731
IFNB1	*NES*	-0.01262864031	0.8671287243
MX1	*NES*	0.09971417978	0.185408961
CXCL10	*NES*	0.1599475278	0.03295084574
DHX58	*NES*	-0.3189575792	1.43E-05
OASL	*NES*	-0.04958371701	0.5110053324
STAT1	*NES*	0.0365379439	0.6282408838
PDCD1LG2	*NES*	-0.09645435042	0.2002696527
KLF4	*NES*	0.03858065573	0.6091450941
MYC	*NES*	0.2727477772	0.0002301830803
POU5F1	*NES*	0.09902694467	0.1884725955
SOX2	*NES*	-0.04943785558	0.5122506685
NANOG	*NES*	-0.1231229905	0.1015618869
HES1	*NES*	0.006037699716	0.9362482541
**GSE25065**			
IFN-I S[31646105]	IFN-I S[19001271]	0.9279245018	0

IFN-I S[31646105]	Viral mimicry	0.8080047986	0
IFN-I S[19001271]	Viral mimicry	0.810753506	0
IFN-I S[31646105]	Yamanaka	0.05507695376	0.4405670809
IFN-I S[19001271]	Yamanaka	0.095713918	0.1796473118
Viral mimicry	Yamanaka	0.1297975804	0.06840103199
IFN-I S[31646105]	CSC S[21169407]	0.087223535	0.221537684
IFN-I S[19001271]	CSC S[21169407]	0.1504917295	0.03440712724
Viral mimicry	CSC S[21169407]	0.176115928	0.01315801846
Yamanaka	CSC S[21169407]	0.1790238688	0.01170512831
IFN-I S[31646105]	CSC S[22909066]	0.3808714392	4.00E-08
IFN-I S[19001271]	CSC S[22909066]	0.4013800737	6.32E-09
Viral mimicry	CSC S[22909066]	0.2868109197	4.51E-05
Yamanaka	CSC S[22909066]	0.1656219878	0.01980111483
"21169407"	CSC S[22909066]	0.7887823984	0
IFN-I S[31646105]	*IFNB1*	-0.08058446385	0.2590746784
IFN-I S[19001271]	*IFNB1*	-0.08908257991	0.212013029
Viral mimicry	*IFNB1*	0.1585578093	0.02567365325
Yamanaka	*IFNB1*	0.09607416257	0.1781604989
CSC S[21169407]	*IFNB1*	-0.1969856191	0.005409323066
CSC S[22909066]	*IFNB1*	-0.279054973	6.86E-05
IFN-I S[31646105]	*MX1*	0.8145901074	3.02E-48
IFN-I S[19001271]	*MX1*	0.872340812	7.96E-63
Viral mimicry	*MX1*	0.8225919718	6.11E-50
Yamanaka	*MX1*	0.06218914222	0.384092204
CSC S[21169407]	*MX1*	0.2416977371	0.000602563028
CSC S[22909066]	*MX1*	0.3826625756	2.64E-08
IFNB1	*MX1*	-0.06154450266	0.3890499215
IFN-I S[31646105]	*CXCL10*	0.7399584292	1.35E-35
IFN-I S[19001271]	*CXCL10*	0.7194938227	7.31E-33
Viral mimicry	*CXCL10*	0.662070794	2.40E-26
Yamanaka	*CXCL10*	0.2570169058	0.0002569349307
CSC S[21169407]	*CXCL10*	0.4007313923	4.91E-09
CSC S[22909066]	*CXCL10*	0.5792206367	3.92E-19
"3456 (IFNB1)"	*CXCL10*	-0.1150470763	0.1065340247
"4599 (MX1)"	*CXCL10*	0.5990755946	1.13E-20
IFN-I S[31646105]	*DHX58*	0.4467878541	5.87E-11
IFN-I S[19001271]	*DHX58*	0.4163928394	1.50E-09
Viral mimicry	*DHX58*	0.6038862208	0
Yamanaka	*DHX58*	-0.02768727502	0.6983640692
CSC S[21169407]	*DHX58*	-0.1090091281	0.1262575642
CSC S[22909066]	*DHX58*	-0.07774528696	0.2760281689
IFNB1	*DHX58*	0.01992272543	0.7805592726
MX1	*DHX58*	0.4331132795	1.85E-10
"3627 (CXCL10)"	*DHX58*	0.1695853048	0.01691756774
IFN-I S[31646105]	*OASL*	0.6601015075	3.79E-26
IFN-I S[19001271]	*OASL*	0.6559204698	9.86E-26
Viral mimicry	*OASL*	0.7314821543	1.97E-34

Yamanaka	*OASL*	0.1011001022	0.1564153733
CSC S[21169407]	*OASL*	0.07195187749	0.3137672997
CSC S[22909066]	*OASL*	0.09518527508	0.1822281292
IFNB1	*OASL*	0.2220913312	0.001662670977
MX1	*OASL*	0.6512859459	2.80E-25
CXCL10	*OASL*	0.469896328	2.87E-12
DHX58	*OASL*	0.4552314373	1.60E-11
IFN-I S[31646105]	*STAT1*	0.8692643611	7.01E-62
IFN-I S[19001271]	*STAT1*	0.8354195355	7.65E-53
Viral mimicry	*STAT1*	0.7170743415	1.48E-32
Yamanaka	*STAT1*	0.0922340097	0.1962232249
CSC S[21169407]	*STAT1*	0.2538690429	0.0003074478149
CSC S[22909066]	*STAT1*	0.5118858838	1.28E-14
IFNB1	*STAT1*	-0.1034043494	0.14714091
MX1	*STAT1*	0.7344828546	7.71E-35
CXCL10	*STAT1*	0.8033329301	5.37E-46
DHX58	*STAT1*	0.2981830774	1.98E-05
OASL	*STAT1*	0.5509858371	4.08E-17
IFN-I S[31646105]	*PDCD1LG2*	0.1369818964	0.05430658256
IFN-I S[19001271]	*PDCD1LG2*	0.0850755954	0.2333781386
Viral mimicry	*PDCD1LG2*	0.1174711379	0.09930934413
Yamanaka	*PDCD1LG2*	-0.004783192967	0.9466774541
CSC S[21169407]	*PDCD1LG2*	-0.160761044	0.02366517374
CSC S[22909066]	*PDCD1LG2*	-0.1645362726	0.02053746018
IFNB1	*PDCD1LG2*	0.04774382853	0.5041702119
MX1	*PDCD1LG2*	0.107784209	0.1306707538
CXCL10	*PDCD1LG2*	0.05853313901	0.4127168179
DHX58	*PDCD1LG2*	0.2088356891	0.003151115311
OASL	*PDCD1LG2*	0.1726704623	0.01499063744
STAT1	*PDCD1LG2*	0.05099497873	0.4755462709
IFN-I S[31646105]	*KLF4*	0.1552115891	0.02900329302
IFN-I S[19001271]	*KLF4*	0.1621498363	0.02246990943
Viral mimicry	*KLF4*	0.0354000565	0.6205123862
Yamanaka	*KLF4*	0.3321106116	1.75E-06
CSC S[21169407]	*KLF4*	-0.1555053206	0.02869694373
CSC S[22909066]	*KLF4*	0.01650307239	0.8174979754
IFNB1	*KLF4*	-0.07086275158	0.3211674257
MX1	*KLF4*	-0.006898828011	0.9231539497
CXCL10	*KLF4*	0.06051419324	0.3970534966
DHX58	*KLF4*	0.09331927029	0.1909887059
OASL	*KLF4*	-0.01717480323	0.810207409
STAT1	*KLF4*	0.09349477235	0.1901518806
PDCD1LG2	*KLF4*	0.004244428366	0.9526761089
IFN-I S[31646105]	*MYC*	0.05855544227	0.4125384779
IFN-I S[19001271]	*MYC*	0.1231122898	0.08399637991
Viral mimicry	*MYC*	0.1638188849	0.02110265884
Yamanaka	*MYC*	0.4953895696	1.17E-13

CSC S[21169407]	*MYC*	0.3932110842	1.00E-08
CSC S[22909066]	*MYC*	0.3606934236	1.79E-07
IFNB1	*MYC*	-0.1556910759	0.02850463961
MX1	*MYC*	0.1646823656	0.02042397924
CXCL10	*MYC*	0.2921530964	2.96E-05
DHX58	*MYC*	-0.1028795769	0.1492154981
OASL	*MYC*	-0.020030981	0.7793974243
STAT1	*MYC*	0.06527110493	0.3609242537
PDCD1LG2	*MYC*	-0.06711630914	0.3474789965
KLF4	*MYC*	-0.1176535609	0.09878174296
IFN-I S[31646105]	*POU5F1*	0.03670485693	0.6076813158
IFN-I S[19001271]	*POU5F1*	0.01677130211	0.814584853
Viral mimicry	*POU5F1*	0.1378745271	0.05273997406
Yamanaka	*POU5F1*	0.5508820451	4.14E-17
CSC S[21169407]	*POU5F1*	0.2027219645	0.004179437977
CSC S[22909066]	*POU5F1*	0.1416814434	0.04647349846
IFNB1	*POU5F1*	0.07659209359	0.2834960723
MX1	*POU5F1*	0.09724297991	0.172914464
CXCL10	*POU5F1*	0.2273795389	0.00127494973
DHX58	*POU5F1*	-0.02041280113	0.7753034758
OASL	*POU5F1*	0.1057241379	0.1382314982
STAT1	*POU5F1*	0.1314712288	0.06485256395
PDCD1LG2	*POU5F1*	-0.03848819139	0.5903372527
KLF4	*POU5F1*	-0.1758873307	0.01318851044
MYC	*POU5F1*	0.1513755936	0.0332674982
IFN-I S[31646105]	*SOX2*	-0.2974841907	2.27E-05
IFN-I S[19001271]	*SOX2*	-0.2723825248	0.0001097542225
Viral mimicry	*SOX2*	-0.222083344	0.001701546823
Yamanaka	*SOX2*	0.2720393229	0.000112035629
CSC S[21169407]	*SOX2*	-0.08485513245	0.2343753648
CSC S[22909066]	*SOX2*	-0.2822673589	6.00E-05
IFNB1	*SOX2*	0.4083053354	2.35E-09
MX1	*SOX2*	-0.2365868091	0.000791465474
CXCL10	*SOX2*	-0.2425738021	0.0005747115549
DHX58	*SOX2*	-0.07928505781	0.2666070511
OASL	*SOX2*	0.09245589001	0.1951446396
STAT1	*SOX2*	-0.2627134529	0.0001846101906
PDCD1LG2	*SOX2*	0.09932468705	0.163856334
KLF4	*SOX2*	-0.09375832159	0.1889002692
MYC	*SOX2*	-0.243942747	0.0005335630181
POU5F1	*SOX2*	0.06829569188	0.3390531822
IFN-I S[31646105]	*NANOG*	-0.1162241883	0.1029757435
IFN-I S[19001271]	*NANOG*	-0.1504485591	0.03437442139
Viral mimicry	*NANOG*	-0.04535757186	0.5257393938
Yamanaka	*NANOG*	-0.02785425965	0.6968780856
CSC S[21169407]	*NANOG*	-0.1365674215	0.05504699705
CSC S[22909066]	*NANOG*	-0.1950254232	0.005898621172

IFNB1	*NANOG*	0.1492829661	0.03581036957
MX1	*NANOG*	-0.0805574719	0.2592347759
CXCL10	*NANOG*	-0.1902912276	0.00724728727
DHX58	*NANOG*	0.06733874521	0.3458798248
OASL	*NANOG*	0.0855478524	0.2307841339
STAT1	*NANOG*	-0.1952767165	0.005833757337
PDCD1LG2	*NANOG*	0.05304648582	0.4579498449
KLF4	*NANOG*	-0.1306425961	0.06657456617
MYC	*NANOG*	-0.1243993952	0.08078302647
POU5F1	*NANOG*	0.04136362688	0.5628588805
SOX2	*NANOG*	0.2547858928	0.0002918565357
IFN-I S[31646105]	*HES1*	-0.1487154276	0.03661010238
IFN-I S[19001271]	*HES1*	-0.09203299995	0.1970272644
Viral mimicry	*HES1*	-0.1310513497	0.06575730985
Yamanaka	*HES1*	-0.01468425035	0.8371782529
CSC S[21169407]	*HES1*	-0.08148495129	0.2535324824
CSC S[22909066]	*HES1*	-0.1975954221	0.00533227813
IFNB1	*HES1*	0.008070659431	0.9101518208
MX1	*HES1*	-0.1037830685	0.145657365
CXCL10	*HES1*	-0.1521384064	0.03237950316
DHX58	*HES1*	-0.04243568249	0.5524583001
OASL	*HES1*	-0.1663610827	0.01915845325
STAT1	*HES1*	-0.1405427926	0.04827902167
PDCD1LG2	*HES1*	-0.178176257	0.01202476954
KLF4	*HES1*	0.06946518064	0.3308276492
MYC	*HES1*	-0.06448573352	0.3667438796
POU5F1	*HES1*	-0.01204145017	0.8662910115
SOX2	*HES1*	0.02470358252	0.7295256572
NANOG	*HES1*	-0.0726552874	0.3090475889
IFN-I S[31646105]	*NES*	-0.07711147536	0.2802342857
IFN-I S[19001271]	*NES*	-0.007848814851	0.9126115862
Viral mimicry	*NES*	-0.07020723917	0.3256754311
Yamanaka	*NES*	0.1459946038	0.04013773918
CSC S[21169407]	*NES*	0.1252934899	0.07860971906
CSC S[22909066]	*NES*	0.1439485321	0.0430463257
IFNB1	*NES*	-0.04435192758	0.5349675408
MX1	*NES*	-0.001937082689	0.9783922461
CXCL10	*NES*	0.07942743913	0.2659984235
DHX58	*NES*	-0.1674926577	0.01834428398
OASL	*NES*	-0.1689946838	0.01731010538
STAT1	*NES*	-0.0951705885	0.1822959056
PDCD1LG2	*NES*	-0.1255874641	0.0779055472
KLF4	*NES*	-0.04217444875	0.5552226739
MYC	*NES*	0.3324960684	1.70E-06
POU5F1	*NES*	0.01218909366	0.8646671663
SOX2	*NES*	-0.1057139666	0.1382696409
NANOG	*NES*	-0.04920856692	0.4911637669

HES1	*NES*	-0.1448281806	0.0417748935
**GSE41998**			
IFN-I S[31646105]	IFN-I S[19001271]	0.920372642	0
IFN-I S[31646105]	Viral mimicry	0.8131369559	0
IFN-I S[19001271]	Viral mimicry	0.8027693974	0
IFN-I S[31646105]	Yamanaka	0.209274608	0.0004450962996
IFN-I S[19001271]	Yamanaka	0.2441291207	3.96E-05
Viral mimicry	Yamanaka	0.1710633337	0.004204583416
IFN-I S[31646105]	CSC S[21169407]	0.2410171402	4.99E-05
IFN-I S[19001271]	CSC S[21169407]	0.3412414769	6.14E-09
Viral mimicry	CSC S[21169407]	0.3058294378	2.17E-07
Yamanaka	CSC S[21169407]	0.283351015	1.67E-06
IFN-I S[31646105]	CSC S[22909066]	0.3896131021	2.12E-11
IFN-I S[19001271]	CSC S[22909066]	0.4465095205	0
Viral mimicry	CSC S[22909066]	0.309119893	1.59E-07
Yamanaka	CSC S[22909066]	0.2992921792	3.99E-07
"21169407"	CSC S[22909066]	0.8453917051	0
IFN-I S[31646105]	*IFNB1*	0.05666077	0.3457166161
IFN-I S[19001271]	*IFNB1*	0.05289622299	0.3787559595
Viral mimicry	*IFNB1*	0.3430230036	4.02E-09
Yamanaka	*IFNB1*	0.03050067702	0.6119495712
CSC S[21169407]	*IFNB1*	0.003667850144	0.951366912
CSC S[22909066]	*IFNB1*	-0.06317454695	0.2930123883
IFN-I S[31646105]	*MX1*	0.8041324383	1.60E-64
IFN-I S[19001271]	*MX1*	0.87986931	1.71E-91
Viral mimicry	*MX1*	0.8149970245	1.34E-67
Yamanaka	*MX1*	0.2135172306	0.0003282273613
CSC S[21169407]	*MX1*	0.424044317	1.33E-13
CSC S[22909066]	*MX1*	0.4087957701	1.15E-12
IFNB1	*MX1*	0.04607083132	0.4433866872
IFN-I S[31646105]	*CXCL10*	0.7418782545	5.34E-50
IFN-I S[19001271]	*CXCL10*	0.7204319865	6.25E-46
Viral mimicry	*CXCL10*	0.6516141821	4.17E-35
Yamanaka	*CXCL10*	0.2582495236	1.25E-05
CSC S[21169407]	*CXCL10*	0.5999158737	1.16E-28
CSC S[22909066]	*CXCL10*	0.6852456412	5.07E-40
"3456 (IFNB1)"	*CXCL10*	0.01556406151	0.7957744293
"4599 (MX1)"	*CXCL10*	0.6288715562	4.03E-32
IFN-I S[31646105]	*DHX58*	0.5449553882	5.57E-23
IFN-I S[19001271]	*DHX58*	0.515890213	2.24E-20
Viral mimicry	*DHX58*	0.6605347895	2.39E-36
Yamanaka	*DHX58*	-0.01074027056	0.8582536614
CSC S[21169407]	*DHX58*	0.009143389174	0.8791536344
CSC S[22909066]	*DHX58*	0.03540960146	0.5558704506
IFNB1	*DHX58*	0.1016710899	0.09007302926
MX1	*DHX58*	0.5497525523	1.95E-23
"3627 (CXCL10)"	*DHX58*	0.2557360337	1.53E-05

IFN-I S[31646105]	*OASL*	0.8181143062	1.61E-68
IFN-I S[19001271]	*OASL*	0.8649346509	6.46E-85
Viral mimicry	*OASL*	0.7819245383	8.54E-59
Yamanaka	*OASL*	0.2199900596	0.0002126174132
CSC S[21169407]	*OASL*	0.4610835187	4.31E-16
CSC S[22909066]	*OASL*	0.4642620828	2.55E-16
IFNB1	*OASL*	0.09921460024	0.09815731769
MX1	*OASL*	0.8633131071	2.99E-84
CXCL10	*OASL*	0.6815419237	1.90E-39
DHX58	*OASL*	0.4894125077	3.28E-18
IFN-I S[31646105]	*STAT1*	0.880247533	1.14E-91
IFN-I S[19001271]	*STAT1*	0.8366996744	2.11E-74
Viral mimicry	*STAT1*	0.7385484637	2.43E-49
Yamanaka	*STAT1*	0.2316589484	9.41E-05
CSC S[21169407]	*STAT1*	0.4486119576	3.21E-15
CSC S[22909066]	*STAT1*	0.559290941	2.32E-24
IFNB1	*STAT1*	0.03936490018	0.5125802089
MX1	*STAT1*	0.7510562069	7.25E-52
CXCL10	*STAT1*	0.8504522171	2.99E-79
DHX58	*STAT1*	0.4101756488	9.54E-13
OASL	*STAT1*	0.777559519	9.51E-58
IFN-I S[31646105]	*PDCD1LG2*	0.4239885677	1.34E-13
IFN-I S[19001271]	*PDCD1LG2*	0.38668235	2.20E-11
Viral mimicry	*PDCD1LG2*	0.2956869728	4.91E-07
Yamanaka	*PDCD1LG2*	0.1603897714	0.007265787871
CSC S[21169407]	*PDCD1LG2*	0.291680682	7.11E-07
CSC S[22909066]	*PDCD1LG2*	0.4158859198	4.28E-13
IFNB1	*PDCD1LG2*	0.006790334672	0.9100994959
MX1	*PDCD1LG2*	0.3069035687	1.69E-07
CXCL10	*PDCD1LG2*	0.4740118606	4.92E-17
DHX58	*PDCD1LG2*	0.1097306918	0.06722519817
OASL	*PDCD1LG2*	0.3563826121	8.87E-10
STAT1	*PDCD1LG2*	0.4276290279	7.85E-14
IFN-I S[31646105]	*KLF4*	-0.03495923122	0.5609099433
IFN-I S[19001271]	*KLF4*	-0.04039994928	0.5015473931
Viral mimicry	*KLF4*	-0.1165586747	0.05179823611
Yamanaka	*KLF4*	0.410547528	9.06E-13
CSC S[21169407]	*KLF4*	-0.1161155268	0.05270107789
CSC S[22909066]	*KLF4*	-0.08528856692	0.1553766095
IFNB1	*KLF4*	-0.007270500984	0.9037720722
MX1	*KLF4*	-0.1226824167	0.04058529354
CXCL10	*KLF4*	-0.08676339214	0.1483301031
DHX58	*KLF4*	-0.1268706034	0.03415732887
OASL	*KLF4*	-0.1226213256	0.04068612855
STAT1	*KLF4*	-0.06182579802	0.303459543
PDCD1LG2	*KLF4*	-0.06836362411	0.2550774706
IFN-I S[31646105]	*MYC*	0.2173394604	0.0002543838566

IFN-I S[19001271]	*MYC*	0.2966441492	4.49E-07
Viral mimicry	*MYC*	0.2096067504	0.0004240616224
Yamanaka	*MYC*	0.5714812219	1.37E-25
CSC S[21169407]	*MYC*	0.5988019535	1.55E-28
CSC S[22909066]	*MYC*	0.576850108	3.80E-26
IFNB1	*MYC*	0.04079807535	0.497337228
MX1	*MYC*	0.3213843573	4.01E-08
CXCL10	*MYC*	0.4306400522	5.03E-14
DHX58	*MYC*	0.03740295075	0.5338331526
OASL	*MYC*	0.3201415753	4.55E-08
STAT1	*MYC*	0.2689831932	5.19E-06
PDCD1LG2	*MYC*	0.2960265988	4.76E-07
KLF4	*MYC*	-0.1364401399	0.02264105347
IFN-I S[31646105]	*POU5F1*	0.1760874493	0.003166146354
IFN-I S[19001271]	*POU5F1*	0.1935434409	0.001157812421
Viral mimicry	*POU5F1*	0.1812364184	0.002374759252
Yamanaka	*POU5F1*	0.6023882188	6.07E-29
CSC S[21169407]	*POU5F1*	0.1468004601	0.01411381024
CSC S[22909066]	*POU5F1*	0.157250903	0.008508258134
IFNB1	*POU5F1*	0.004019274726	0.9467138662
MX1	*POU5F1*	0.1892162233	0.001497977732
CXCL10	*POU5F1*	0.1965397975	0.000965570042
DHX58	*POU5F1*	0.02195518813	0.7150206869
OASL	*POU5F1*	0.1857381225	0.001835306665
STAT1	*POU5F1*	0.2039597382	0.0006089324046
PDCD1LG2	*POU5F1*	0.1024653017	0.0875763886
KLF4	*POU5F1*	-0.06046345155	0.3142589814
MYC	*POU5F1*	0.2386749655	5.65E-05
IFN-I S[31646105]	*SOX2*	0.04020932387	0.5035698726
IFN-I S[19001271]	*SOX2*	-0.02411012282	0.6884431542
Viral mimicry	*SOX2*	-0.01185918117	0.8436671252
Yamanaka	*SOX2*	0.2993174444	3.50E-07
CSC S[21169407]	*SOX2*	-0.04651451275	0.4390038986
CSC S[22909066]	*SOX2*	-0.0169866021	0.7775764407
IFNB1	*SOX2*	-0.03624807729	0.5465471455
MX1	*SOX2*	-0.06514361635	0.2781963512
CXCL10	*SOX2*	-0.01663753786	0.7820310751
DHX58	*SOX2*	-0.002237292528	0.9703236058
OASL	*SOX2*	-0.03010589002	0.6165658175
STAT1	*SOX2*	0.04960469604	0.4091728329
PDCD1LG2	*SOX2*	0.01399342903	0.8159956351
KLF4	*SOX2*	0.06836968331	0.2550352583
MYC	*SOX2*	0.01068998075	0.8589104285
POU5F1	*SOX2*	-0.004803625194	0.9363354049
IFN-I S[31646105]	*NANOG*	-0.2463860589	3.16E-05
IFN-I S[19001271]	*NANOG*	-0.273978643	3.40E-06
Viral mimicry	*NANOG*	-0.2128383302	0.0003432680962

Yamanaka	*NANOG*	-0.2061568542	0.0005295680758
CSC S[21169407]	*NANOG*	-0.5115837991	5.20E-20
CSC S[22909066]	*NANOG*	-0.5126916687	4.19E-20
IFNB1	*NANOG*	-0.06280489734	0.2958514416
MX1	*NANOG*	-0.2476769605	2.87E-05
CXCL10	*NANOG*	-0.4521633449	1.83E-15
DHX58	*NANOG*	-0.0164627995	0.784263708
OASL	*NANOG*	-0.3196909236	4.76E-08
STAT1	*NANOG*	-0.344930285	3.25E-09
PDCD1LG2	*NANOG*	-0.2543664874	1.70E-05
KLF4	*NANOG*	0.1004365588	0.09406660368
MYC	*NANOG*	-0.3246616956	2.86E-08
POU5F1	*NANOG*	-0.1653132329	0.005641042963
SOX2	*NANOG*	-0.09882656943	0.0994854799
IFN-I S[31646105]	*HES1*	-0.2960678359	4.74E-07
IFN-I S[19001271]	*HES1*	-0.2687200292	5.30E-06
Viral mimicry	*HES1*	-0.1670117824	0.005161272296
Yamanaka	*HES1*	-0.06012970088	0.3169425226
CSC S[21169407]	*HES1*	-0.1695748018	0.004506544311
CSC S[22909066]	*HES1*	-0.2534245194	1.83E-05
IFNB1	*HES1*	0.03632266702	0.5457215028
MX1	*HES1*	-0.2322988697	8.99E-05
CXCL10	*HES1*	-0.2912146833	7.42E-07
DHX58	*HES1*	-0.08993014056	0.1340233081
OASL	*HES1*	-0.2504794784	2.31E-05
STAT1	*HES1*	-0.2737296304	3.47E-06
PDCD1LG2	*HES1*	-0.2346469795	7.58E-05
KLF4	*HES1*	0.1610575103	0.007023373066
MYC	*HES1*	-0.1956421198	0.001019829927
POU5F1	*HES1*	-0.1169435444	0.05102460477
SOX2	*HES1*	0.01056703755	0.8605164313
NANOG	*HES1*	0.1894775288	0.001475085695
IFN-I S[31646105]	*NES*	0.03703435613	0.5378748113
IFN-I S[19001271]	*NES*	0.03158286177	0.5993743387
Viral mimicry	*NES*	-0.0538585098	0.3701319213
Yamanaka	*NES*	0.1278407474	0.03279855663
CSC S[21169407]	*NES*	0.1257636982	0.03576580434
CSC S[22909066]	*NES*	0.171563213	0.004051225336
IFNB1	*NES*	-0.01168817068	0.8458931617
MX1	*NES*	0.040202721	0.5036400038
CXCL10	*NES*	0.02152418992	0.7203790679
DHX58	*NES*	-0.05148757319	0.3916000069
OASL	*NES*	-0.01518859905	0.8005964998
STAT1	*NES*	-0.02386149019	0.6914909124
PDCD1LG2	*NES*	0.1452744681	0.01515870707
KLF4	*NES*	-0.0962330473	0.1087323856
MYC	*NES*	0.3213786936	4.01E-08

POU5F1	*NES*	0.02672453746	0.6567078022
SOX2	*NES*	-0.01455813716	0.8087104701
NANOG	*NES*	-0.05216853165	0.3853585269
HES1	*NES*	-0.1178355528	0.04926850989

Supplementary Table 3. Statistic of box plot survival curves.

Value	Comparison	Gene	PI	Minimum	WiskerLow	Q1	Q2/Median	Q3	WiskerHigh	Maximum
**DSS**	*KDM1B vs.* IFN-I S[31646105]	*KDM1B*	Low	-0,997947371	-0,997947371	-0,558356254	-0,116630404	0,436512172	0,991953693	0,991953693
**DSS**	*KDM1B vs.* IFN-I S[31646105]	*KDM1B*	High	-0,999014738	-0,999014738	-0,4099922	0,10357568	0,549694158	0,999507369	0,999507369
**DSS**	*KDM1B vs.* IFN-I S[31646105]	IFN-I S[31646105]	Low	-0,692240014	-0,692240014	-0,456664216	-0,347236489	-0,232899206	0,074222139	0,074222139
**DSS**	*KDM1B vs.* IFN-I S[31646105]	IFN-I S[31646105]	High	-0,201870739	-0,201870739	0,162263852	0,325822747	0,49290255	0,760399004	0,760399004
**DSS**	*KDM1B vs.* IFN-I S[19001271]	*KDM1B*	Low	-0,994745269	-0,994745269	-0,565212037	-0,116630404	0,433474281	0,999261053	0,999261053
**DSS**	*KDM1B vs.* IFN-I S[19001271]	*KDM1B*	High	-0,999014738	-0,999014738	-0,406543783	0,118272507	0,578102549	0,999507369	0,999507369
**DSS**	*KDM1B vs.* IFN-I S[19001271]	IFN-I S[19001271]	Low	-0,659070857	-0,659070857	-0,40408291	-0,284092452	-0,156813425	0,098287115	0,098287115
**DSS**	*KDM1B vs.* IFN-I S[19001271]	IFN-I S[19001271]	High	-0,116638029	-0,116638029	0,149862026	0,287657582	0,42902922	0,706741835	0,706741835
**DSS**	*KDM1B vs.* viral mimicry	*KDM1B*	Low	-0,997947371	-0,997947371	-0,555975204	-0,095118847	0,436676382	0,991953693	0,991953693
**DSS**	*KDM1B vs.* viral mimicry	*KDM1B*	High	-0,999014738	-0,999014738	-0,40904799	0,098403054	0,576501498	0,999507369	0,999507369
**DSS**	*KDM1B vs.* viral mimicry	viral mimicry	Low	-0,873085607	-0,873085607	-0,581386391	-0,425346226	-0,250793752	0,073745586	0,073745586
**DSS**	*KDM1B vs.* viral mimicry	viral mimicry	High	-0,190571629	-0,190571629	0,144002616	0,400049914	0,586498762	0,90790522	0,90790522
**DSS**	*KDM1B vs.* YAMAKAKA	*KDM1B*	Low	-0,999014738	-0,999014738	-0,641282483	-0,210476621	0,266102878	0,990065274	0,990065274
**DSS**	*KDM1B vs.* YAMAKAKA	*KDM1B*	High	-0,997947371	-0,997947371	-0,304158627	0,24520711	0,652530892	0,999507369	0,999507369
**DSS**	*KDM1B vs.* YAMAKAKA	YAMAKAKA	Low	-0,869999977	-0,869999977	-0,449899599	-0,264483043	-0,113508104	0,21225165	0,21225165
**DSS**	*KDM1B vs.* YAMAKAKA	YAMAKAKA	High	-0,178579069	-0,178579069	0,123039361	0,295763381	0,497847424	0,867417443	0,867417443
**DSS**	*KDM1B vs.* CSC S[21169407]	*KDM1B*	Low	-0,999014738	-0,999014738	-0,559628885	-0,085019911	0,461020567	0,999261053	0,999261053
**DSS**	*KDM1B vs.* CSC S[21169407]	*KDM1B*	High	-0,998768422	-0,998768422	-0,412291145	0,106777782	0,537049961	0,999507369	0,999507369

**DSS**	*KDM1B vs.* CSC S[21169407]	CSC S[21169407]	Low	-0,742502088	-0,742502088	-0,539410996	-0,405212803	-0,22023117	0,051664727	0,051664727
**DSS**	*KDM1B vs.* CSC S[21169407]	CSC S[21169407]	High	0,002324565	0,002324565	0,223318053	0,37521267	0,504251765	0,735048976	0,735048976
**DSS**	*KDM1B vs.* CSC S[22909066]	*KDM1B*	Low	-0,999014738	-0,999014738	-0,565335194	-0,10800936	0,441684798	0,999261053	0,999261053
**DSS**	*KDM1B vs.* CSC S[22909066]	*KDM1B*	High	-0,997044214	-0,997044214	-0,40871957	0,106859888	0,542304692	0,999507369	0,999507369
**DSS**	*KDM1B vs.* CSC S[22909066]	CSC S[22909066]	Low	-0,476097458	-0,476097458	-0,257036429	-0,172118809	-0,095353256	-0,004927502	-0,004927502
**DSS**	*KDM1B vs.* CSC S[22909066]	CSC S[22909066]	High	-0,024953891	-0,024953891	0,077676107	0,16407475	0,273841589	0,491793609	0,491793609
**DRFI**	*KDM1B vs.* IFN-I S[31646105]	*KDM1B*	Low	-0,998768422	-0,998768422	-0,567141508	-0,137485118	0,400919578	0,991953693	0,991953693
**DRFI**	*KDM1B vs.* IFN-I S[31646105]	*KDM1B*	High	-0,999014738	-0,999014738	-0,399831684	0,136540909	0,584629911	0,999507369	0,999507369
**DRFI**	*KDM1B vs.* IFN-I S[31646105]	IFN-I S[31646105]	Low	-0,692240014	-0,692240014	-0,456664216	-0,347236489	-0,232718885	0,103199112	0,119338845
**DRFI**	*KDM1B vs.* IFN-I S[31646105]	IFN-I S[31646105]	High	-0,247840678	-0,247840678	0,162391408	0,325815104	0,492774674	0,760399004	0,760399004
**DRFI**	*KDM1B vs.* IFN-I S[19001271]	*KDM1B*	Low	-0,994745269	-0,994745269	-0,594975163	-0,159160885	0,363397512	0,999261053	0,999261053
**DRFI**	*KDM1B vs.* IFN-I S[19001271]	*KDM1B*	High	-0,999014738	-0,999014738	-0,374728027	0,170532452	0,59715095	0,999507369	0,999507369
**DRFI**	*KDM1B vs.* IFN-I S[19001271]	IFN-I S[19001271]	Low	-0,659070857	-0,659070857	-0,40408291	-0,284092452	-0,156099347	0,157934358	0,157934358
**DRFI**	*KDM1B vs.* IFN-I S[19001271]	IFN-I S[19001271]	High	-0,174356577	-0,174356577	0,14767826	0,287551494	0,428950417	0,706741835	0,706741835
**DRFI**	*KDM1B vs.* viral mimicry	*KDM1B*	Low	-0,997947371	-0,997947371	-0,565212037	-0,109979884	0,431298493	0,991953693	0,991953693
**DRFI**	*KDM1B vs.* viral mimicry	*KDM1B*	High	-0,999014738	-0,999014738	-0,400899052	0,108666201	0,579498337	0,999507369	0,999507369
**DRFI**	*KDM1B vs.* viral mimicry	viral mimicry	Low	-0,873085607	-0,873085607	-0,581386391	-0,425346226	-0,250793752	0,097156885	0,097156885
**DRFI**	*KDM1B vs.* viral mimicry	viral mimicry	High	-0,209223211	-0,209223211	0,143527616	0,399401857	0,586468312	0,90790522	0,90790522
**DRFI**	*KDM1B vs.* YAMAKAKA	*KDM1B*	Low	-0,999014738	-0,999014738	-0,652038261	-0,23363028	0,248286054	0,990065274	0,990065274
**DRFI**	*KDM1B vs.* YAMAKAKA	*KDM1B*	High	-0,997947371	-0,997947371	-0,293977585	0,263311302	0,667104561	0,999507369	0,999507369

**DRFI**	*KDM1B vs.* YAMAKAKA	YAMAKAKA	Low	-0,869999977	-0,869999977	-0,449899599	-0,264483043	-0,107964238	0,21225165	0,21225165
**DRFI**	*KDM1B vs.* YAMAKAKA	YAMAKAKA	High	-0,209832874	-0,209832874	0,120832407	0,295532131	0,497762729	0,867417443	0,867417443
**DRFI**	*KDM1B vs.* CSC S[21169407]	*KDM1B*	Low	-0,999014738	-0,999014738	-0,558807833	-0,079436758	0,46914898	0,999261053	0,999261053
**DRFI**	*KDM1B vs.* CSC S[21169407]	*KDM1B*	High	-0,998768422	-0,998768422	-0,415472721	0,10673673	0,536536804	0,999507369	0,999507369
**DRFI**	*KDM1B vs.* CSC S[21169407]	CSC S[21169407]	Low	-0,742502088	-0,742502088	-0,539410996	-0,405212803	-0,22023117	0,051664727	0,051664727
**DRFI**	*KDM1B vs.* CSC S[21169407]	CSC S[21169407]	High	-0,002073564	-0,002073564	0,222947393	0,375210063	0,503703749	0,735048976	0,735048976
**DRFI**	*KDM1B vs.* CSC S[22909066]	*KDM1B*	Low	-0,999014738	-0,999014738	-0,586313067	-0,134939858	0,405106942	0,999261053	0,999261053
**DRFI**	*KDM1B vs.* CSC S[22909066]	*KDM1B*	High	-0,997044214	-0,997044214	-0,392626955	0,130506178	0,563426249	0,999507369	0,999507369
**DRFI**	*KDM1B vs.* CSC S[22909066]	CSC S[22909066]	Low	-0,476097458	-0,476097458	-0,257036429	-0,172118809	-0,095353256	0,019834827	0,019834827
**DRFI**	*KDM1B vs.* CSC S[22909066]	CSC S[22909066]	High	-0,041784917	-0,041784917	0,077451115	0,163842265	0,273816487	0,491793609	0,491793609

Supplementary Table 4. Patient characteristics.

Parameter	Value (*n* = 20)
**Age**	
**Median range**	53.5 (30-77)
**Sex**	
**Female**	20 (100%)
**Male**	0 (0%)
**Histological type**	
**Invasive ductal carcinoma**	19 (95%)
**Ductal carcinoma *in situ***	0 (0%)
**Invasive lobular carcinoma**	1 (5%)
**Histological grade at diagnosis**	
**II**	6 (30%)
**II/III**	6 (30%)
**III**	7 (35%)
**Unknown**	1 (5%)
**ER* *status* at diagnosis**	
**Positive**	15 (75%)
**Negative**	5 (25%)
**Unknown**	0 (0%)
**PR** *status* at diagnosis**	
**Positive**	13 (65%)
**Negative**	7 (35%)
**Unknown**	0 (0%)
**HER2*** *status* at diagnosis**	
**Positive**	16 (80%)
**Negative**	4 (20%)
**Unknown**	0 (0%)
**Ki-67 *status* at diagnosis**	
**Positive**	19 (95%)
**Negative**	0 (0%)
**Unknown**	1 (5%)
**Number of chemotherapy cycles**	
**4**	1 (5%)
**5**	1 (5%)
**7**	1 (5%)
**8**	14 (70%)
**10**	1 (5%)
**Unknown**	2 (10%)
**Histological grade at surgery**	
**II**	4 (20%)
**II/III**	0 (0%)
**III**	13 (65%)
**Unknown**	3 (15%)
**ER *status* at surgery**	
**Positive**	14 (70%)
**Negative**	6 (30%)
**Unknown**	0 (0%)
**PR *status* at surgery**	
**Positive**	10 (50%)
**Negative**	10 (50%)
**Unknown**	0 (0%)
**HER2 *status* at surgery**	
**Positive**	13 (65%)
**Negative**	7 (35%)
**Unknown**	0 (0%)
**Ki-67 *status* at surgery**	
**Positive**	19 (95%)
**Negative**	0 (0%)
**Unknown**	1 (5%)

Abbreviations: *ER, estrogen receptor; **PR, progesterone receptor; ***HER2, human epidermal growth factor receptor 2.

Supplementary Table 5. Methods: Key Resources Table.

REAGENT or RESOURCE	SOURCE	IDENTIFIER
Antibodies		
Rat monoclonal anti-CD133 (13A4)	eBioscience™	Cat# 17-1331-81
Rat monoclonal anti-CD24 (M1/69)	eBioscience™	Cat# 12-0242-82
Rat monoclonal anti-CD44 (IM7)	eBioscience™	Cat# 11-0441-82
Rat monoclonal CD44 (IM7)	BioLegend®	Cat#103020
Rat monoclonal anti-CD8a (53-6.7)	eBioscience™	Cat# 17-0081-82
Rat monoclonal anti-CD273 (122)	eBioscience™	Cat# 11-9972-81
Mouse monoclonal anti-CD66a (CC1)	eBioscience™	Cat# 12-0661-80
Mouse monoclonal anti-H2-K1 (AF6-88.5.5.3)	eBioscience™	Cat# 11-5958-80
Rat monoclonal anti-CD274 (10F.9G2)	BioLegend®	Cat# 124312
Rat monoclonal anti-Galectin-9 (108A2)	BioLegend®	Cat# 137903
Rat monoclonal anti-CD366 (RMT3-23)	eBioscience™	Cat# 11-5870-82
Rat monoclonal anti-CD45 (30-F11)	eBioscience™	Cat# MCD4528
Mouse monoclonal anti-CD271 (ME20.4)	BioLegend®	Cat#53-9400-42
Mouse monoclonal anti-CD133/1 (AC133)	Miltenyi Biotec	Cat# 130-113-106
Recombinant monoclonal anti-CD44 (REA690)	Miltenyi Biotec	Cat# 130-113-342
Human recombinant monoclonal anti-CD133/1 (REA753)	Miltenyi Biotec	Cat# 130-111-080
Human recombinant monoclonal anti-CD24 (REA832)	Miltenyi Biotec	Cat# 130-112-845
Human recombinant monoclonal anti-CD44 (REA690)	Miltenyi Biotec	Cat# 130-113-903
Human recombinant monoclonal anti-CD44 (REAL259)	Miltenyi Biotec	Cat# 130-120-881
Rat monoclonal anti-CD4 (GK1.5)	Miltenyi Biotec	Cat# 130-120-750
Mouse monoclonal anti-CD24 (ML5)	R&D Systems	Cat# BBA13
Mouse monoclonal anti-CD44v6 (2F10)	R&D Systems	Cat# BBA13
Rabbit polyclonal anti-MX1	Sigma-Aldrich	Cat# HPA030917
Rabbit monoclonal anti-CD44 (SP37)	Sigma-Aldrich	Cat# SAB5500068
Mouse monoclonal anti-CD24 (SN3)	Millipore	Cat# CBL561
Mouse monoclonal anti-CD45 (2B11+PD7/26)	Agilent Technologies	Cat# M0701
Rabbit monoclonal anti-CD133 (EPR16508)	Abcam	Cat# AB 222782
Rabbit monoclonal-IP10 (EPR24674-12)	Abcam	Cat# AB 283681
Goat anti-mouse Alexa Fluor® Plus 488	Thermo Scientific	Cat# A32723
Goat anti-mouse Alexa Fluor™ 488	Thermo Scientific	Cat# A21121
Goat anti-Rabbit IgG (H+L) Highly Cross-Adsorbed Secondary Antibody, Alexa Fluor™ 555	Invitrogen	Cat# A-21429
Rabbit recombinant anti-LSD2/AOF1 (EPR18508)	Abcam	Cat# AB193080
Mouse monoclonal anti-β-Actin (AC-15)	Sigma-Aldrich	Cat# A5441
Rabbit IgG HRP linked whole antibody	GE Healthcare	Cat# GEHNA9341ML
Mouse IgG HRP linked whole antibody	GE Healthcare	Cat# GEHNA9311ML
Rabbit anti-Numb (C29G11)	Cell Signaling Technology	Cat# 2756
InVivoMAb rat anti-CD4 (GK1.5)	Bio Cell	Cat# BE0003-1
InVivoMAb rat anti-CD8a (2.43)	Bio Cell	Cat# BE0061
Chemicals, Peptides, Recombinant Proteins and Oligonucleotides		
Purified mouse IFN-α/β and mock	Dr. E. Proietti, Istituto Superiore di Sanità, Rome	N/A
Recombinant human Roferon-A®	Hoffmann-La Roche Ltd	N/A
Cisplatin	Sigma-Aldrich	Cat# P4394
Doxorubicin hydrochloride	Sigma-Aldrich	Cat# D1515
Mitoxantrone dihydrochloride	Sigma-Aldrich	Cat# M6545
Oxaliplatin	Selleck Chemicals	Cat# S1224
Oxaliplatin	Sigma-Aldrich	Cat# O9512
Cytochalasin D	Sigma-Aldrich	Cat# C8273
Tranylcypromine	Calbiochem	Cat# 616431

Thalidomide	Sigma-Aldrich	Cat# T144
Amlexanox	InvivoGen	Cat# inh-amx
MRT67307	InvivoGen	Cat# inh-mrt
BX795	InvivoGen	Cat# tlrl-bx7
Verapamil	Sigma-Aldrich	Cat# V4629
Benzonase nuclease	Sigma-Aldrich	Cat# E8263
DNase I	Qiagen	Cat# 79254
Ribonuclease A	Sigma-Aldrich	Cat# R6513
Ribonuclease H	Thermo Scientific	Cat# EN0201
Collagenase A	Sigma-Aldrich	Cat# COLLA-RO
Trypan Blue Stain (0.4%)	Thermo Scientific	Cat# 15250061
DMSO	Sigma-Aldrich	Cat# 5879
Hoechst 33342	Thermo Scientific	Cat# H1399
DAPI	Thermo Scientific	Cat# D1306
PI	Thermo Scientific	Cat# P1304MP
CFSE	Sigma-Aldrich	Cat# 21888
SeaPlaque GTG Agarose	Lonza	Cat# 50111
Ppia (Mm00620857_s1) primers	Applied Biosystems™	Cat# 4331182
Klf4 (Mm00516104_m1) primers	Applied Biosystems™	Cat# 4331182
Myc (Mm00487804_m1) primers	Applied Biosystems™	Cat# 4331182
Pou5f1 (Mm03053917_g1) primers	Applied Biosystems™	Cat# 4331182
Sox2 (Mm03053810_s1) primers	Applied Biosystems™	Cat# 4331182
Nanog (Mm02019550_s1) primers	Applied Biosystems™	Cat# 4331182
Hes1 (Mm01342805_m1) primers	Applied Biosystems™	Cat# 4331182
Nes (Mm00450205_m1) primers	Applied Biosystems™	Cat# 4331182
Twist1 (Mm00442036_m1) primers	Applied Biosystems™	Cat# 4331182
Snai1 (Mm00441533_g1) primers	Applied Biosystems™	Cat# 4331182
Cdh2 (Mm01162497_m1) primers	Applied Biosystems™	Cat# 4331182
Vim (Mm00619195_g1) primers	Applied Biosystems™	Cat# 4331182
Fn1 (Mm01256744_m1) primers	Applied Biosystems™	Cat# 4331182
Lgals9 (Mm00495295_m1) primers	Applied Biosystems™	Cat# 4331182
Pdcd1lg2 (Mm00451734_m1) primers	Applied Biosystems™	Cat# 4331182
Pdl1 (fw: GCATTATATTCACAGCCTGC, rw: CCCTTCAAAAGCTGGTCCTT) primers	Sigma-Aldrich	Custom
Ifnb1 (Mm00439552_s1) primers	Applied Biosystems™	Cat# 4331182
Agarose gel	Sigma-Aldrich	Cat# A9539
Puromycin dihydrochloride	Sigma-Aldrich	Cat# P9620
Polybrene Infection/Transfection Reagent	Merck Millipore	Cat# TR-1003
SYBR Safe DNA gel stain	Thermo Scientific	Cat# S33102
SYTOX™ Blue Dead Cell Stain	Thermo Scientific	Cat# S34857
Viobility 405/452 Fixable Dye	Miltenyi Biotec	Cat# 130-109-816
Crystal Violet	Sigma-Aldrich	Cat# C0775
Matrigel	BD Biosciences	Cat# 354234
PKH26 Red Fluorescent Cell Linker	Sigma-Aldrich	Cat# PKH26GL
CD45 MicroBeads	Miltenyi	Cat# 130-052-301
Agencourt AMPure SPRI beads	Beckman Coulter	Cat# A63880
Critical Commercial Assays		
RNeasy Plus Mini Kit	Qiagen	Cat# 74134
RNeasy Mini Kit	Qiagen	Cat# 74104
GoTaq® Probe 1-Step RT-qPCR System	Promega	Cat# A6120

Mouse magnetic Luminex screening assay CCL11/Eotaxin (BR47), CCL12/MCP-5 (BR74), CCL2/MCP-1/JE (BR5), CCL22/MDC (BR48), CCL3/MIP-1 alpha (BR27), CCL4/MIP-1 beta (BR31), CCL5/RANTES (BR21), CCL8/MCP-2 (BR72), CXCL1/KC (BR2), CXCL10/IP-10 (BR20), CXCL12/SDF-1 alpha (BR34), CXCL2/MIP-2 (BR7)	R&D Systems	Cat# LXSAMSM-12
Mouse pluripotent stem cell transcription factor analysis kit assayssing Nanog (M55-312)	BD Biosciences	Cat# 560585
CellTiter-Glo® Luminescent Cell Viability Assay	Promega	Cat# G7572
ExoEasy Maxi Kit	Qiagen	Cat# 76064
Nextera Library Prep Kit	Illumina	Cat# 20018704
MinElute PCR purification kit	Qiagen	Cat# 28004
KAPA Library Quantification Kit for Illumina platforms	KAPA Biosystems - Hoffmann - La Roche	Cat# KR0405
TruSeq Stranded mRNA Library kit	Illumina	Cat# 20020594
ChromoPlex TM1 Dual Detection	Bond III, Leica Biosystems	Cat# DS9665
Experimental Models: Cell Lines		
MCA205 mouse fibrosarcoma cell line	Sigma-Aldrich	Cat#SCC173; RRID:CVCL_VR90
Panel of MCA-derived cancer cell clones	Pr. Laurence Zitvogel, Gustave Roussy cancer Campus, Villejuif	N/A
MCA205-OVA mouse fibrosarcoma cell line	Dr. Oliver Kepp, Gustave Roussy cancer Campus, Villejuif	N/A
AT3 mouse mammary carcinoma cell line	Sigma-Aldrich	Cat# SCC178, RRID:CVCL_VR89
CT26 WT mouse colon carcinoma cell line	ATCC	Cat# CRL-2638, RRID:CVCL_7256
B16.F10 murine melanoma cell line	ATCC	Cat# CRL-6475, RRID:CVCL_0159
MDA-MB-231 human breast carcinoma cell line	ATCC	Cat# HTB-26, RRID:CVCL_0062
U2OS human bone osteosarcoma cell line	ATCC	Cat# HTB-96, RRID:CVCL_0042
MCF7 human breast cell line	Dr. Ilio Vitale, I.R.C.C.S Institute of Candiolo	N/A
MCF10-A human breast epithelial cell line	Dr.Ilio Vitale, I.R.C.C.S Institute of Candiolo	N/A
HMLER human mammary epithelial cell line	Pr. Robert Weinberg, Whitehead Institute for Biomedical Research	N/A
Experimental Models: Organisms/Strains		
Mouse: C57BL/6J	Charles River	Cat# 000664; RRID:IMSR_JAX:000664
Mouse : NOD.Cg-Prkdcscid Il2rgtm1Wjl/SzJ (NSG)	Charles River	Cat# 005557; RRID:IMSR_JAX:005557
Mouse: C57BL/6-Tg(TcraTcrb)1100Mjb/Crl OT1	Charles River	Strain Code 642
Software and Algorithms		
Flowjo v.10.0.7	Flowjo, LLC	RRID: SCR_008520
Illustrator CC 2015and CC 2020	Adobe Systems Inc.	RRID: SCR_010279
Photoshop CC 2015	Adobe Systems Inc.	RRID: SCR_014199
ImageJ v.1.5	National Institute of Health	http://rsb.info.nih.gov/ij/; RRID: SCR_003070

NIS-Elements Software	Nikon Instruments Inc.	N/A
Leica Application Suite X (LAS X) Software	Leica Microsystems	N/A
ELDA: Extreme Limiting Dilution Analysis	Walter+Eliza Hall *Bioinformatics*	https://bioinf.wehi.edu.au/software/elda/
Bio-Plex Manager Software v.6.1	Bio-Rad	RRID:SCR_014330
BWA MEM v.0.7.17 algorithm		RRID:SCR_010910
STAR alignment – DESeq2 software		RRID:SCR_015687
"R" software	R Foundation for Statistical Computing	http://www.R-project.org/
HOMER software		RRID:SCR_010881
EnrichmentMap software		N/A
GraphPad Prism v.8.4.0	GraphPad Software	N/A
Microsoft Excel	Microsoft, Redmond	RRID:SCR_016137
SPSS software v.21	SPSS Inc.	N/A
Other		
Roswell Park Memorial Institute 1640 (RPMI 1640)	EuroClone	Cat# ECB9006L
Dulbecco's Modified Eagle Medium high glucose (DMEM)	EuroClone	Cat# ECB7501LX60
Fetal bovine serum (FBS)	EuroClone	Cat# ECS0180L
Penicillin G sodium salt and streptomycin sulfate	EuroClone	Cat# ECB3001D/1
L-glutamine 200 mM	EuroClone	Cat# ECB3004D
Dulbecco's Modified Eagle Medium (DMEM)	Thermo Scientific	Cat# 52100047
Ham's F-12 Nutrient Mix (F-12)	Thermo Scientific	Cat# 21700018
Dulbecco's Modified Eagle Medium/Nutrient Mixture F-12 (DMEM/F-12)	Thermo Scientific	Cat# 11330032
HuMEC Basal Serum-Free Medium (1X)	Thermo Scientific	Cat# 12753018
Bovine Pituitary Extract	Thermo Scientific	Cat# 13028014
HuMEC Supplement	Thermo Scientific	Cat# 12754016
Horse Serum	Sigma-Aldrich	Cat# H1270
Cholera Toxin from Vibrio cholerae	Sigma-Aldrich	Cat# C8052
Hydrocortisone 21-hemisuccinate sodium salt	Sigma-Aldrich	Cat# H2270
Bovine serum albumin (BSA)	US Biological, Salem	Cat# A1312
Penicillin-Streptomycin-Amphotericin B (PSF)	Lonza	Cat# 17-745E
Sodium Bicarbonate 7.5% solution	Thermo Scientific	Cat# 25080094
Hepes buffer 1M	Thermo Scientific	Cat# 15630056
L-glutamine 200mM	Thermo Scientific	Cat# 25030024
Human apotransferrin	Sigma-Aldrich	Cat# T2252
Heparin sodium salt	Sigma-Aldrich	Cat# H3393
D-(+)-Glucose solution	Sigma-Aldrich	Cat# G8769
Recombinant human insulin	Sigma-Aldrich	Cat# 91077C
Progesterone	Sigma-Aldrich	Cat# P8783
Putrescine dihydrochloride	Sigma-Aldrich	Cat# P5780
Sodium selenite	Sigma-Aldrich	Cat# S5261
Recombinant human epidermal growth factor (EGF)	PeproTech Inc.	Cat# AF-100-15
Recombinant human fibroblast growth factor basic (bFGF)	PeproTech Inc.	Cat# AF-100-18B
Nicotinamide	Sigma-Aldrich	Cat# N0636
Trypsin-EDTA w/ Phenol Red	EuroClone	Cat# ECM0920
Dulbecco's Phosphate-Buffered Saline (D-PBS)	EuroClone	Cat# ECB4053L
Paraformaldehyde	Sigma-Aldrich	Cat# 158127
TWEEN® 20	Sigma-Aldrich	Cat# P1379
Bio-Rad Protein Assay Dye Reagent Concentrate	Bio-Rad	Cat# 5000006
Blotting-Grade Blocker	Bio-Rad	Cat# 1706404
NuPAGE™ MOPS SDS Running Buffer (20X)	Invitrogen	Cat# NP0001

NuPAGE™ LDS Sample Buffer (4X)	Invitrogen	Cat# NP0007
NuPAGE™ Sample Reducing Agent (10X)	Invitrogen	Cat# NP0009
NuPAGE™ 4 to 12%, Bis-Tris, 1.0–1.5 mm, Mini Protein Gels	Invitrogen	Cat# NP0336PK2
SeeBlue™ Plus2 Pre-stained Protein Standard	Invitrogen	Cat# LC5925
SuperSignal™ West Dura Extended Duration Substrate	Thermo Scientific	Cat# 34075
T-PER™ Tissue Protein Extraction Reagent	Thermo Scientific	Cat# 78510
MACS columns	Miltenyi Biotec	Cat# 130-042-201, Cat# 130-042-401
MACS separators	Miltenyi Biotec	Cat# 130-042-109, Cat# 130-042-302
Transwell® Permeable Supports, Polycarbonate (PC) Membrane	Corning	Cat# 3422
KDM1B MISSION shRNA Lentiviral Transduction Particles Mouse TRCN0000076613 (Sequence:CCGGGCCAAGAAACTTCAGTTGATACTCGAGTATCAACTGAAGTTTCTTGGCTTTTTG) 1mL 10^7 VP hPGK-Puro-CMV-tGFP	Sigma-Aldrich	Cat# SHCLNV
MISSION® pLKO.1-puro-CMV-TurboGFP™ Positive Control Transduction Particles SHC003V	Sigma-Aldrich	Cat# SHCLNV
MISSION® pLKO.1-puro Non-Mammalian shRNA Control Transduction Particles SHC002V	Sigma-Aldrich	Cat# SHCLNV
LV *Kdm1b*^{OVER}	Dr. Eliana Ruggiero, IRCCS San Raffaele Scientific Institute	N/A

Supplementary Table 6. ELDA Statistics.

MCA205

The value of the confidence choice entered was "0.95"
The value of the observed choice is "TRUE"
The value of the test_unit_slope choice is "TRUE"
The value of the test_difference choice is "TRUE"
Limiting dilution data entered.

Counter	Dose	Tested	Response	Group
1	50	60	60	ki
2	25	60	60	ki
3	10	60	54	ki
4	5	60	40	ki
5	1	60	8	ki
6	50	60	60	ko
7	25	60	38	ko
8	10	60	42	ko
9	5	60	27	ko
10	1	60	8	ko

The number of lines of data entered = 10

Plot results has been checked
Confidence intervals for
1/(stem cell frequency)

Group	Lower	Estimate	Upper
ki	6.18	5.13	4.28
ko	15.85	13.25	11.09

Overall test for differences in stem cell frequencies between any of the groups

Chisq	DF	P.value
53.1	1	3.21e-13

Goodness of fit tests. These test whether the log-dose slope equals 1. Rejection of the tests may be due either to batch effects (heterogeneity in the stem cell frequencies or assay success rate) or to a failure of the stem cell hypothesis.
Estimated slope is 0.861

Test	Chisq	DF	P Value
Likelihood ratio test of single-hit model	3.62	1	0.057
Score test of heterogeneity	2.9	1	0.0888

The value of the confidence choice entered was "0.95"
The value of the observed choice is "TRUE"
The value of the test_unit_slope choice is "TRUE"
The value of the test_difference choice is "TRUE"
Limiting dilution data entered.

Counter	Dose	Tested	Response	Group
1	50	60	60	ctr

2	25	60	56	ctr
3	10	60	42	ctr
4	1	60	13	ctr
5	50	60	60	ko
6	25	60	38	ko
7	10	60	42	ko
8	1	60	27	ko
9	50	60	60	ki
10	25	60	60	ki
11	10	60	40	ki
12	1	60	8	ki

The number of lines of data entered = 12

Plot results has been checked
Confidence intervals for
1/(stem cell frequency)

Group	Lower	Estimate	Upper
ctr	10.38	8.39	6.80
ki	9.82	7.92	6.41
ko	14.68	12.02	9.86

Overall test for differences in stem cell frequencies between any of the groups

Chisq	DF	P.value
10.8	2	0.00455

Pairwise tests for differences in stem cell frequencies

Group 1	Group 2	Chisq	DF	Pr(>Chisq)
ctr	ki	0.145	1	0.703
ctr	ko	6.6	1	0.0102
ki	ko	8.37	1	0.00382

Goodness of fit tests. These test whether the log-dose slope equals 1. Rejection of the tests may be due either to batch effects (heterogeneity in the stem cell frequencies or assay success rate) or to a failure of the stem cell hypothesis.
Estimated slope is 0.674

Test	Chisq	DF	P Value
Likelihood ratio test of single-hit model	34.1	1	5.34e-09
Score test of heterogeneity	9.79	1	0.00176

The value of the confidence choice entered was "0.95"
The value of the observed choice is "TRUE"
The value of the test_unit_slope choice is "TRUE"
The value of the test_difference choice is "TRUE"
Limiting dilution data entered.

Counter	Dose	Tested	Response	Group
1	50	60	60	ctr
2	25	60	56	ctr

3	10	60	42	ctr
4	1	60	13	ctr
5	50	60	60	ko
6	25	60	38	ko
7	10	60	42	ko
8	1	60	27	ko

The number of lines of data entered = 8

Plot results has been checked
Confidence intervals for
1/(stem cell frequency)

Group	Lower	Estimate	Upper
ctr	10.4	8.39	6.80
ko	14.7	12.02	9.86

Overall test for differences in stem cell frequencies between any of the groups

Chisq	DF	P.value
6.6	1	0.0102

Goodness of fit tests. These test whether the log-dose slope equals 1. Rejection of the tests may be due either to batch effects (heterogeneity in the stem cell frequencies or assay success rate) or to a failure of the stem cell hypothesis.
Estimated slope is 0.547

Test	Chisq	DF	P Value
Likelihood ratio test of single-hit model	52	1	5.44e-13
Score test of heterogeneity	18.4	1	1.78e-05

The value of the confidence choice entered was "0.95"
The value of the observed choice is "TRUE"
The value of the test_unit_slope choice is "TRUE"
The value of the test_difference choice is "TRUE"
Limiting dilution data entered.

Counter	Dose	Tested	Response	Group
1	50	60	60	ctr
2	25	60	56	ctr
3	10	60	42	ctr
4	1	60	13	ctr
5	50	60	60	ki
6	25	60	60	ki
7	10	60	40	ki
8	1	60	8	ki

The number of lines of data entered = 8

Plot results has been checked
Confidence intervals for
1/(stem cell frequency)

Group	Lower	Estimate	Upper

ctr	10.38	8.39	6.80
ki	9.82	7.92	6.41

Overall test for differences in stem cell frequencies between any of the groups

Chisq	DF	P.value
0.145	1	0.703

Goodness of fit tests. These test whether the log-dose slope equals 1. Rejection of the tests may be due either to batch effects (heterogeneity in the stem cell frequencies or assay success rate) or to a failure of the stem cell hypothesis.

Estimated slope is 0.919

Test	Chisq	DF	P Value
Likelihood ratio test of single-hit model	0.957	1	0.328
Score test of heterogeneity	0.0205	1	0.886

CT26

The value of the confidence choice entered was "0.95"
The value of the observed choice is "TRUE"
The value of the test_unit_slope choice is "TRUE"
The value of the test_difference choice is "TRUE"
A short data line was detected and removed.
The short data line was
Limiting dilution data entered.

Counter	Dose	Tested	Response	Group
1	50	60	60	ki
2	25	60	60	ki
3	10	60	56	ki
4	5	60	44	ki
5	1	60	23	ki
6	50	60	60	ko
7	25	60	50	ko
8	10	60	32	ko
9	5	60	18	ko
10	1	60	11	ko

The number of lines of data entered = 10
Plot results has been checked

Confidence intervals for
1/(stem cell frequency)

Group	Lower	Estimate	Upper
ki	4.62	3.83	3.2
ko	15.15	12.66	10.6

Overall test for differences in stem cell frequencies between any of the groups

Chisq	DF	P.value
83.3	1	7.1e-20

Goodness of fit tests. These test whether the log-dose slope equals 1. Rejection of the tests may be due either to batch effects (heterogeneity in the stem cell frequencies or assay success rate) or to a failure of the stem cell hypothesis.
Estimated slope is 0.839

Test	Chisq	DF	P Value
Likelihood ratio test of single-hit model	4.58	1	0.0324
Score test of heterogeneity	0.00347	1	0.953

The value of the confidence choice entered was "0.95"
The value of the observed choice is "TRUE"
The value of the test_unit_slope choice is "TRUE"
The value of the test_difference choice is "TRUE"
Limiting dilution data entered.

Counter	Dose	Tested	Response	Group
1	50	60	60	ctr
2	25	60	60	ctr
3	10	60	59	ctr
4	5	60	46	ctr
5	1	60	26	ctr
6	50	60	60	ko
7	25	60	50	ko

8	10	60	32	ko
9	5	60	18	ko
10	1	60	11	ko
11	50	60	60	ki
12	25	60	60	ki
13	10	60	56	ki
14	5	60	44	ki
15	1	60	23	ki

The number of lines of data entered = 15
Plot results has been checked
Confidence intervals for
1/(stem cell frequency)

Group	Lower	Estimate	Upper
ctr	3.87	3.21	2.68
ki	4.62	3.83	3.20
ko	15.15	12.66	10.60

Overall test for differences in stem cell frequencies between any of the groups

Chisq	DF	P.value
142	2	1.78e-31

Pairwise tests for differences in stem cell frequencies

Group 1	Group 2	Chisq	DF	Pr(>Chisq)
ctr	ki	1.91	1	0.167
ctr	ko	108	1	2.64e-25
ki	ko	83.3	1	7.1e-20

Goodness of fit tests. These test whether the log-dose slope equals 1. Rejection of the tests may be due either to batch effects (heterogeneity in the stem cell frequencies or assay success rate) or to a failure of the stem cell hypothesis.
Estimated slope is 0.827

Test	Chisq	DF	P Value
Likelihood ratio test of single-hit model	7.28	1	0.00695
Score test of heterogeneity	0.0448	1	0.832

The value of the confidence choice entered was "0.95"
The value of the observed choice is "TRUE"
The value of the test_unit_slope choice is "TRUE"
The value of the test_difference choice is "TRUE"
Limiting dilution data entered.

Counter	Dose	Tested	Response	Group
1	50	60	60	ctr
2	25	60	60	ctr
3	10	60	59	ctr
4	5	60	46	ctr
5	1	60	26	ctr
6	50	60	60	ko
7	25	60	50	ko
8	10	60	32	ko
9	5	60	18	ko
10	1	60	11	ko

The number of lines of data entered = 10
Plot results has been checked
Confidence intervals for
1/(stem cell frequency)

Group	Lower	Estimate	Upper
ctr	3.87	3.21	2.68
ko	15.15	12.66	10.60

Overall test for differences in stem cell frequencies between any of the groups

Chisq	DF	P.value

108	1	2.64e-25

Goodness of fit tests. These test whether the log-dose slope equals 1. Rejection of the tests may be due either to batch effects (heterogeneity in the stem cell frequencies or assay success rate) or to a failure of the stem cell hypothesis.
Estimated slope is 0.85

Test	Chisq	DF	P Value
Likelihood ratio test of single-hit model	3.82	1	0.0507
Score test of heterogeneity	0.00456	1	0.946

The value of the confidence choice entered was "0.95"
The value of the observed choice is "TRUE"
The value of the test_unit_slope choice is "TRUE"
The value of the test_difference choice is "TRUE"
Limiting dilution data entered.

Counter	Dose	Tested	Response	Group
1	50	60	60	ctr
2	25	60	60	ctr
3	10	60	59	ctr
4	5	60	46	ctr
5	1	60	26	ctr
6	50	60	60	ki
7	25	60	60	ki
8	10	60	56	ki
9	5	60	44	ki
10	1	60	23	ki

The number of lines of data entered = 10
Plot results has been checked
Confidence intervals for
1/(stem cell frequency)

Group	Lower	Estimate	Upper

ctr	3.87	3.21	2.68
ki	4.62	3.83	3.20

Overall test for differences in stem cell frequencies between any of the groups

Chisq	DF	P.value
1.91	1	0.167

Goodness of fit tests. These test whether the log-dose slope equals 1. Rejection of the tests may be due either to batch effects (heterogeneity in the stem cell frequencies or assay success rate) or to a failure of the stem cell hypothesis.
Estimated slope is 0.784

Test	Chisq	DF	P Value
Likelihood ratio test of single-hit model	6.59	1	0.0103
Score test of heterogeneity	2.09	1	0.149

B16.F10

The value of the confidence choice entered was "0.95"
The value of the observed choice is "TRUE"
The value of the test_unit_slope choice is "TRUE"
The value of the test_difference choice is "TRUE"
Limiting dilution data entered.

Counter	Dose	Tested	Response	Group
1	50	60	44	ko
2	25	60	29	ko
3	10	60	20	ko
4	5	60	6	ko
5	1	60	3	ko
6	50	60	60	ki
7	25	60	60	ki
8	10	60	54	ki

9	5	60	40	ki
10	1	60	14	ki

The number of lines of data entered = 10
Plot results has been checked
Confidence intervals for
1/(stem cell frequency)

Group	Lower	Estimate	Upper
ki	5.78	4.8	4.01
ko	43.55	35.6	29.13

Overall test for differences in stem cell frequencies between any of the groups

Chisq	DF	P.value
217	1	3.81e-49

Goodness of fit tests. These test whether the log-dose slope equals 1. Rejection of the tests may be due either to batch effects (heterogeneity in the stem cell frequencies or assay success rate) or to a failure of the stem cell hypothesis.
Estimated slope is 0.929

Test	Chisq	DF	P Value
Likelihood ratio test of single-hit model	0.702	1	0.402
Score test of heterogeneity	0.624	1	0.43

The value of the confidence choice entered was "0.95"
The value of the observed choice is "TRUE"
The value of the test_unit_slope choice is "TRUE"
The value of the test_difference choice is "TRUE"
Limiting dilution data entered.

Counter	Dose	Tested	Response	Group
1	50	60	60	ctr
2	25	60	60	ctr
3	10	60	60	ctr
4	5	60	59	ctr

5	1	60	36	ctr
6	50	60	44	ko
7	25	60	29	ko
8	10	60	20	ko
9	5	60	6	ko
10	1	60	3	ko
11	50	60	60	ki
12	25	60	60	ki
13	10	60	54	ki
14	5	60	40	ki
15	1	60	14	ki

The number of lines of data entered = 15
Plot results has been checked
Confidence intervals for
1/(stem cell frequency)

Group	Lower	Estimate	Upper
ctr	2.06	1.7	1.45
ki	5.78	4.8	4.01
ko	43.55	35.6	29.13

Overall test for differences in stem cell frequencies between any of the groups

Chisq	DF	P.value
535	2	7.23e-117

Pairwise tests for differences in stem cell frequencies

Group 1	Group 2	Chisq	DF	Pr(>Chisq)
ctr	ki	58.5	1	2.07e-14
ctr	ko	455	1	5.29e-101

ki	ko	217	1	3.81e-49

Goodness of fit tests. These test whether the log-dose slope equals 1. Rejection of the tests may be due either to batch effects (heterogeneity in the stem cell frequencies or assay success rate) or to a failure of the stem cell hypothesis.
Estimated slope is 0.931

Test	Chisq	DF	P Value
Likelihood ratio test of single-hit model	0.811	1	0.368
Score test of heterogeneity	0.659	1	0.417

The value of the confidence choice entered was "0.95"
The value of the observed choice is "TRUE"
The value of the test_unit_slope choice is "TRUE"
The value of the test_difference choice is "TRUE"
Limiting dilution data entered.

Counter	Dose	Tested	Response	Group
1	50	60	60	ctr
2	25	60	60	ctr
3	10	60	60	ctr
4	5	60	59	ctr
5	1	60	36	ctr
6	50	60	44	ko
7	25	60	29	ko
8	10	60	20	ko
9	5	60	6	ko
10	1	60	3	ko

The number of lines of data entered = 10
Plot results has been checked
Confidence intervals for
1/(stem cell frequency)

Group	Lower	Estimate	Upper

ctr	2.06	1.7	1.45
ko	43.55	35.6	29.13

Overall test for differences in stem cell frequencies between any of the groups

Chisq	DF	P.value
455	1	5.29e-101

Goodness of fit tests. These test whether the log-dose slope equals 1. Rejection of the tests may be due either to batch effects (heterogeneity in the stem cell frequencies or assay success rate) or to a failure of the stem cell hypothesis.
Estimated slope is 0.903

Test	Chisq	DF	P Value
Likelihood ratio test of single-hit model	1	1	0.316
Score test of heterogeneity	0.848	1	0.357

The value of the confidence choice entered was "0.95"
The value of the observed choice is "TRUE"
The value of the test_unit_slope choice is "TRUE"
The value of the test_difference choice is "TRUE"
Limiting dilution data entered.

Counter	Dose	Tested	Response	Group
1	50	60	60	ctr
2	25	60	60	ctr
3	10	60	60	ctr
4	5	60	59	ctr
5	1	60	36	ctr
6	50	60	60	ki
7	25	60	60	ki
8	10	60	54	ki
9	5	60	40	ki
10	1	60	14	ki

The number of lines of data entered = 10
Plot results has been checked
Confidence intervals for
1/(stem cell frequency)

Group	Lower	Estimate	Upper
ctr	2.06	1.7	1.45
ki	5.78	4.8	4.01

Overall test for differences in stem cell frequencies between any of the groups

Chisq	DF	P.value
58.5	1	2.07e-14

Goodness of fit tests. These test whether the log-dose slope equals 1. Rejection of the tests may be due either to batch effects (heterogeneity in the stem cell frequencies or assay success rate) or to a failure of the stem cell hypothesis.
Estimated slope is 0.965

Test	Chisq	DF	P Value
Likelihood ratio test of single-hit model	0.114	1	0.736
Score test of heterogeneity	0.017	1	0.896
