# Supplementary material for: Type I IFNs promote cancer cell stemness by triggering the epigenetic regulator KDM1B
Source: Nat Immunol. 2022 Aug 24;23(9):1379–92. doi: 10.1038/s41590-022-01290-3 (PMC9477743; doi:10.1038/s41590-022-01290-3)
Supplement: Supplementary file 2 — Reporting Summary [file 41590_2022_1290_MOESM2_ESM.pdf]

nature research

Corresponding author(s): Antonella Sistigu, Ilio Vitale, Ruggero De Maria

Last updated by author(s): Jun 20, 2022

Reporting Summary

Nature Research wishes to improve the reproducibility of the work that we publish. This form provides structure for consistency and transparency in reporting. For further information on Nature Research policies, see our Editorial Policies and the Editorial Policy Checklist.

Statistics

For all statistical analyses, confirm that the following items are present in the figure legend, table legend, main text, or Methods section.

n/a	Confirmed	
☐	☒	The exact sample size (*n*) for each experimental group/condition, given as a discrete number and unit of measurement
☐	☒	A statement on whether measurements were taken from distinct samples or whether the same sample was measured repeatedly
☐	☒	The statistical test(s) used AND whether they are one- or two-sided<br>*Only common tests should be described solely by name; describe more complex techniques in the Methods section.*
☐	☒	A description of all covariates tested
☐	☒	A description of any assumptions or corrections, such as tests of normality and adjustment for multiple comparisons
☐	☒	A full description of the statistical parameters including central tendency (e.g. means) or other basic estimates (e.g. regression coefficient) AND variation (e.g. standard deviation) or associated estimates of uncertainty (e.g. confidence intervals)
☐	☒	For null hypothesis testing, the test statistic (e.g. *F*, *t*, *r*) with confidence intervals, effect sizes, degrees of freedom and *P* value noted<br>*Give P values as exact values whenever suitable.*
☒	☐	For Bayesian analysis, information on the choice of priors and Markov chain Monte Carlo settings
☒	☐	For hierarchical and complex designs, identification of the appropriate level for tests and full reporting of outcomes
☒	☐	Estimates of effect sizes (e.g. Cohen's *d*, Pearson's *r*), indicating how they were calculated

Our web collection on statistics for biologists contains articles on many of the points above.

Software and code

Policy information about availability of computer code

Data collection	Flow Cytometry: BD FACSDiva™(BD Biosciences), MACSQuant® VYB Analyzer 10 (Miltenyi Biotech), CytExpert (Beckman Counter)<br>Cell Sorting: BD FACSDiva™(BD Biosciences)<br>Luminescence Detection: Multimode Detection Software (Beckman Coulter)<br>Conventional immunofluorescence microscopy: EVOS® FL Imaging System operated by embedded Software v.1.4 (Life Technologies-Thermo Scientific), Leica DMI3000 B microscope (HCX PL Fluotar, AN 1.3), Leica DFC 310FX camera, LAS X acquisition software (all from Leica Microsystems, Wetzlar, Germany)<br>Stereo microscopy: ZEN lite imaging (Zeiss)<br>Immunohistochemistry microscopy: LAS V4.8 (Leica)<br>qRT-PCR: StepOnePlus™ Real-Time PCR System operated by embedded StepOne™ Software (ThermoFisher Scientific)<br>RNA quantification: Nanodrop 2000C Spectrophotometer operated by embedded software (ThermoFisher Scientific), Qubit 4 Fluorometer operated by embedded software (ThermoFisher Scientific)<br>Luminex: Bio-Plex Manager v.6.1 (Bio-Rad)
Data analysis	Wet lab:<br>Flow Cytometry: FlowJo v.10.0.7 (FlowJo LLC, TreeStar, Inc.), Prism v.8.4 (GraphPad)<br>Analysis of images: Photoshop CC2015, ImageJ v.1.5, Excel 2013 (Microsoft), Prism v.8.4 (GraphPad), LAS X software<br>Tumor growth: Prism v.8.4 (GraphPad)<br>Luminex assay: Bio-Plex Manager Software v.6.1<br>All other experiments: Excel 2013 (Microsoft), Prism v.8.4 (GraphPad)<br><br>In silico:<br>ATAC-seq and Chip-seq: BWA MEM v.0.7.17, MACS v.2.1.0, HOMER v.4.10, Python v.3.7, matplotlib v.3.2.2, seaborn v.0.11.1<br>RNA-seq: GFF (General Feature Format) GENCODE Release M24 GRCm38 biomaRt v.2.42.1, org.Mm.eg.db v.3.10.0, STAR v.2.7.3.a, GATK v.4.1.2.0, featureCounts v.2.0.0, DESeq2 v.1.26.0, edgeR v.3.28.1, limma-voom v.3.42.2, R v.3.6.3

Network analysis: clusterProfiler v.3.14.3, org.Mm.eg.db v.3.10.0, enrichplot v.1.6.1, R v.3.6.3
Correlation analysis: GEOquery v.2.54.1, affy v.1.64.0, U133x3p.db v.3.2.3, hgu133a.db v.3.2.3, hgu133plus2.db v.3.2.3, hgu133a2.db v.3.2.3, corrplot v.0.84, RColorBrewer v.1.1.2, ggplot2 v.3.1.1, R v.3.6.3
Patient survival analysis: Python v.3.7, pandas v.1.0.0, numpy v.1.17.4, lifelines v.0.26.0, Cox-Regression scipy v.1.5.1, (Wilcoxon rank-sum statistic for two samples) matplotlib v.3.3.1, seaborn v.0.9.0

Figure preparation: Illustrator 2020 (Adobe) CC 2015 and CC 2020
Movie preparation: ImageJ v.1.5 and https://veed.io/ for compression

For manuscripts utilizing custom algorithms or software that are central to the research but not yet described in published literature, software must be made available to editors and reviewers. We strongly encourage code deposition in a community repository (e.g. GitHub). See the Nature Research guidelines for submitting code & software for further information.

Data

Policy information about availability of data

All manuscripts must include a data availability statement. This statement should provide the following information, where applicable:

- Accession codes, unique identifiers, or web links for publicly available datasets
- A list of figures that have associated raw data
- A description of any restrictions on data availability

All data supporting the findings of this study will be available in a publicly accessible repository. The METABRIC patient dataset can be publicly accessed via https://www.cbioportal.org/study/clinicalData?id=brca_metabric. The molecular signature datasets can be publicly accessed at https://www.ncbi.nlm.nih.gov/geo/ with accession codes GSE6861, GSE20271, GSE25065, GSE16446, GSE41998 and GSE32646. All bulk ATAC-seq, Chip-seq, RNA-seq datasets have been uploaded to the Gene Expression Omnibus repository (accession no. HYPERLINK "https://www.ncbi.nlm.nih.gov/geo/query/acc.cgi?acc=GSE173851"GSE173851, https://www.ncbi.nlm.nih.gov/geo/query/acc.cgi?acc=GSE173851).

Field-specific reporting

Please select the one below that is the best fit for your research. If you are not sure, read the appropriate sections before making your selection.

☒ Life sciences ☐ Behavioural & social sciences ☐ Ecological, evolutionary & environmental sciences

For a reference copy of the document with all sections, see nature.com/documents/nr-reporting-summary-flat.pdf

Life sciences study design

All studies must disclose on these points even when the disclosure is negative.

Sample size	In vivo experiments: sample sizes were defined on the basis of our experience with the experimental models used in this study in order to detect differences of 20% or more in continuous endpoints between groups (0.05 significance level and 80% statistical power).<br>In vitro experiments: no statistical methods were used to determine sample size. A minimum of three biologically independent samples were tested, and experiments were performed in at least 2 independent instances (mostly 3) with similar results. When this turned out to be insufficient to clarify statistically sub-significant trends between groups, sample number was increased to improve statistical power.
Data exclusions	Outliers or mice with symptoms not linked to cancer were excluded.
Replication	All experiments were performed in at least 2 independent instances (mostly 3) with similar results. In each individual experiment, each technical replicate was measured once.
Randomization	Mice were randomly allocated to treatment group at tumor detection. For IHC analysis on breast biopsies, intensity score was obtained by calculating 8-10 different fields which were selected randomly. For IF experiments of symmetric vs. asymmetric division, >100 anaphases/telophases were randomly selected. For tracking experiments on microfluidic devices, trajectories of >800 splenocytes were randomly selected.
Blinding	In vivo and in vitro analyses were not blinded but kept as unbiased as possible. Data were analysed by software with objective outcomes, and hence blinding was not relevant for the study. For in vitro studies every sample was processed identically to avoid technical bias. Tumor injections and measurements were performed by the same researcher to ensure reproducibility. Proper internal controls and normalization methods were included in each study for internal bias.

Reporting for specific materials, systems and methods

We require information from authors about some types of materials, experimental systems and methods used in many studies. Here, indicate whether each material, system or method listed is relevant to your study. If you are not sure if a list item applies to your research, read the appropriate section before selecting a response.

Materials & experimental systems

n/a	Involved in the study
☐	☒ Antibodies
☐	☒ Eukaryotic cell lines
☒	☐ Palaeontology and archaeology
☐	☒ Animals and other organisms
☐	☒ Human research participants
☒	☐ Clinical data
☒	☐ Dual use research of concern

Methods

n/a	Involved in the study
☐	☒ ChIP-seq
☐	☒ Flow cytometry
☒	☐ MRI-based neuroimaging

Antibodies

Antibodies used

Rat monoclonal anti-CD133 (13A4) eBioscience™ Cat# 17-1331-81, RRID:AB_823120 https://www.thermofisher.com/antibody/product/CD133-Prominin-1-Antibody-clone-13A4-Monoclonal/17-1331-81

Rat monoclonal anti-CD24 (M1/69) eBioscience™ Cat# 12-0242-82, RRID:AB_465602 https://www.thermofisher.com/antibody/product/CD24-Antibody-clone-M1-69-Monoclonal/12-0242-82

Rat monoclonal anti-CD44 (IM7) eBioscience™ Cat# 11-0441-82, RRID:AB_465045 https://www.thermofisher.com/antibody/product/CD44-Antibody-clone-IM7-Monoclonal/11-0441-82

Rat monoclonal CD44 (IM7) BioLegend® Cat#103020, RRID:AB_493683 https://www.biolegend.com/en-us/products/pacific-blue-anti-mouse-human-cd44-antibody-3099

Rat monoclonal anti-CD8a (53-6.7) eBioscience™ Cat# 17-0081-82, RRID:AB_469335 https://www.thermofisher.com/antibody/product/CD8a-Antibody-clone-53-6-7-Monoclonal/17-0081-82

Rat monoclonal anti-CD273 (122) eBioscience™ Cat# 11-9972-81, RRID:AB_465461 https://www.thermofisher.com/antibody/product/CD273-B7-DC-Antibody-clone-122-Monoclonal/11-9972-81

Mouse monoclonal anti-CD66a (CC1) eBioscience™ Cat# 12-0661-80, RRID:AB_1311201 https://www.thermofisher.com/antibody/product/CD66a-CEACAM1-Antibody-clone-CC1-Monoclonal/12-0661-80

Mouse monoclonal anti-H2-K1 (AF6-88.5.5.3) eBioscience™ Cat# 11-5958-80, RRID:AB_11151335 https://www.thermofisher.com/antibody/product/MHC-Class-I-H-2Kb-Antibody-clone-AF6-88-5-5-3-Monoclonal/11-5958-80

Rat monoclonal anti-CD274 (10F.9G2) BioLegend® Cat# 124312, RRID:AB_10612741 https://www.biolegend.com/en-us/products/apc-anti-mouse-cd274-b7-h1-pd-l1-antibody-6655

Rat monoclonal anti-Galectin-9 (108A2) BioLegend® Cat# 137903, RRID:AB_10568785 https://www.biolegend.com/en-us/products/pe-anti-mouse-galectin-9-antibody-6563

Rat monoclonal anti-CD366 (RMT3-23) eBioscience™ Cat# 11-5870-82, RRID:AB_2688129 https://www.thermofisher.com/antibody/product/CD366-TIM3-Antibody-clone-RMT3-23-Monoclonal/11-5870-82

Rat monoclonal anti-CD45 (30-F11) eBioscience™ Cat# MCD4528, RRID:AB_10373710 https://www.thermofisher.com/antibody/product/CD45-Antibody-clone-30-F11-Monoclonal/MCD4528

Mouse monoclonal anti-CD271 (ME20.4) BioLegend® Cat#53-9400-42, RRID:AB_2802341 https://www.thermofisher.com/antibody/product/CD271-NGF-Receptor-Antibody-clone-ME20-4-Monoclonal/53-9400-42

Mouse monoclonal anti-CD133/1 (AC133) Miltenyi Biotec Cat# 130-113-106 https://www.miltenyibiotec.com/IT-en/products/cd133-1-antibody-anti-human-ac133.html#apc:100-tests-in-200-ul

Recombinant monoclonal anti-CD44 (REA690) Miltenyi Biotec Cat# 130-113-342 https://www.miltenyibiotec.com/IT-en/products/cd44-antibody-anti-human-reafinity-rea690.html#pe:100-tests-in-200-ul

Human recombinant monoclonal anti-CD133/1 (REA753) Miltenyi Biotec Cat# 130-111-080 https://www.miltenyibiotec.com/IT-en/products/cd133-1-antibody-anti-human-reafinity-rea753.html#gref

Human recombinant monoclonal anti-CD24 (REA832) Miltenyi Biotec Cat# 130-112-845 https://www.miltenyibiotec.com/IT-en/products/cd24-antibody-anti-human-reafinity-rea832.html#pe:100-tests-in-200-ul

Human recombinant monoclonal anti-CD44 (REA690) Miltenyi Biotec Cat# 130-113-903 https://www.miltenyibiotec.com/IT-en/products/cd44-antibody-anti-human-reafinity-rea690.html#fitc:30-tests-in-60-ul

Human recombinant monoclonal anti-CD44 (REAL259) Miltenyi Biotec Cat# 130-120-881 https://www.miltenyibiotec.com/IT-en/products/cd44-antibody-anti-human-realease-real259.html#fitc:100-tests-in-200-ul

Rat monoclonal anti-CD4 (GK1.5) Miltenyi Biotec Cat# 130-120-750 https://www.miltenyibiotec.com/IT-en/products/cd4-antibody-

anti-mouse-gk1-5.html#biotin:30-ug-in-1-ml

Mouse monoclonal anti-CD24 (ML5) BD Biosciences Cat# BBA13, RRID:AB_356935 https://www.rndsystems.com/products/human-cd24-alexa-fluor-700-conjugated-antibody-ml5_fab5247n

Mouse monoclonal anti-CD44v6 (2F10) R&D Systems Cat# BBA13, RRID:AB_356935 https://www.rndsystems.com/products/human-cd44v6-antibody-2f10_bba13

Rabbit polyclonal anti-MX1 Sigma-Aldrich Cat# HPA030917, RRID:AB_2680862 Lot. B115464 https://www.sigmaaldrich.com/catalog/product/sigma/hpa030917?lang=it&region=IT

Rabbit monoclonal anti-CD44 (SP37) Sigma-Aldrich Cat# SAB5500068 Lot. 161214C https://www.sigmaaldrich.com/catalog/product/sigma/sab5500068?lang=it&region=IT

Mouse monoclonal anti-CD24 (SN3) Millipore Cat# CBL561, RRID:AB_11212454 Lot. 2983172 https://www.merckmillipore.com/IT/it/product/Anti-CD24-Antibody-clone-SN3,MM_NF-CBL561

Mouse monoclonal anti-CD45 (2B11+PD7/26) Agilent Technologies Cat# M0701, RRID:AB_2661839 Lot. 20049267 https://www.agilent.com/store/productDetail.jsp?catalogId=M070101-2

Rabbit monoclonal anti-CD133 (EPR16508) Abcam Cat# AB 222782 https://www.abcam.com/cd133-antibody-epr16508-ab222782.html

Rabbit monoclonal-IP10 (EPR24674-12) Abcam Cat# AB 283681 https://www.abcam.com/ip10-antibody-epr24674-12-ab283681.html

Goat anti-mouse Alexa Fluor® Plus 488 Thermo Scientific Cat# A32723 https://www.thermofisher.com/antibody/product/Goat-anti-Mouse-IgG-H-L-Highly-Cross-Adsorbed-Secondary-Antibody-Polyclonal/A32723

Goat anti-mouse Alexa FluorTM 488 Thermo Scientific Cat# A21121 https://www.thermofisher.com/antibody/product/Goat-anti-Mouse-IgG1-Cross-Adsorbed-Secondary-Antibody-Polyclonal/A-21121

Goat anti-Rabbit IgG (H+L) Highly Cross-Adsorbed Secondary Antibody, Alexa Fluor™ 555 Invitrogen Cat# A-21429 https://www.thermofisher.com/antibody/product/Goat-anti-Rabbit-IgG-H-L-Highly-Cross-Adsorbed-Secondary-Antibody-Polyclonal/A-21429

Rabbit recombinant anti-LSD2/AOF1 (EPR18508) Abcam Cat# AB193080 https://www.abcam.com/lsd2--aof1-antibody-epr18508-ab193080.html

Mouse monoclonal anti-β-Actin Sigma-Aldrich Cat# A5441 https://www.sigmaaldrich.com/IT/en/product/sigma/a5441

Rabbit IgG HRP linked whole antibody GE Healthcare Cat# GEHNA9341ML https://www.euroclonegroup.it/search_result

Mouse IgG HRP linked whole antibody GE Healthcare Cat# GEHNA9311ML https://www.euroclonegroup.it/search_result

Rabbit anti-Numb (C29G11) Cell Signaling Technology Cat# 2756 https://www.cellsignal.com/products/primary-antibodies/numb-c29g11-rabbit-mab/2756

InVivoMAb rat anti-CD4 (GK1.5) Bio Cell Cat# BE0003-1 https://bxcell.com/product/m-cd4/

InVivoMAb rat anti-CD8a (2.43) Bio Cell Cat# BE0061 https://bxcell.com/product/invivoplus-anti-m-lyt-2-2-cd8a/

Validation

All antibodies were commercial. Specificity and validation were provided by manufacturer's technical datasheets and confirmed in literature. Link to technical datasheet has been provided above. No further validation was performed.

Eukaryotic cell lines

Policy information about cell lines

Cell line source(s)

MCA205 (#SCC173) and AT3 (#SCC178) cells were purchased from Merck Sigma-Aldrich, CT26, B16.F10, U2OS, MC7and MCF10A were from ATCC, MCA clones were kindly provided by Pr. Laurence Zitvogel (Gustave Roussy Cancer Campus, France), OVA-expressing MCA205 cells were kindly provided by Dr. Oliver Kepp (Gustave Roussy Cancer Campus, France), HMLER cells were kindly provided by Pr. Robert Weinberg, Kdm1b OVER and Kdm1b KD MCA205, CT26 and B16.F10 cells were specifically produced for this work.

Authentication

MCA205 and AT3 cells were used shortly after receipt from commercial vendors and hence were not authenticated. CT26, B16.F10, U2OS, MCF7, MCF10A were routinely validated at Candiolo Cancer Institute, just after thawing via STR Profile System using PowerPlex® 16 HS (Promega), HMLER and MCA.205-OVA cells were not authenticated but in all experiments low passage number cells were used. Properties relevant to the experiments (e.g., OVA and MHC-I expression) were routinely confirmed by flow cytometry or (e.g., Kdm1b overexpression or depletion) western blot and qRT-PCR.

Mycoplasma contamination

All cell lines were routinely confirmed to be free from Mycoplasma contamination by PCR.

Commonly misidentified lines (See ICLAC register)	none

Animals and other organisms

Policy information about studies involving animals; ARRIVE guidelines recommended for reporting animal research

Laboratory animals	Six-to-7 week-old female C57Bl/6J, NOD SCID gamma (NSG) and C57BL/6-Tg(TcraTcrb)1100Mjb/J OT1 mice were purchased from Charles River (Calco, Italy), housed in the animal facility at the Istituto Superiore di Sanità (Rome, Italy) and employed after an acclimatization period of 7 days. Mice were maintained in specific pathogen–free conditions in a temperature-controlled environment (20° +/- 2°C) with 12h light - 12h dark cycles and received food and water ad libitum.
Wild animals	None
Field-collected samples	None
Ethics oversight	All the in vivo experimentations were in compliance with the EU Directive 63/2010 and included in an experimental protocol approved by the Institutional Animal Experimentation Committee at the Istituto Superiore di Sanità (Rome) and the Italian Ministry of Health (approval number 858/2015-PR).

Note that full information on the approval of the study protocol must also be provided in the manuscript.

Human research participants

Policy information about studies involving human research participants

Population characteristics	Twenty breast cancer patients (all with bioptic material before, at diagnosis, and after, at surgery, neoadjuvant anthracycline-based chemotherapy) attending the Division of Medical Oncology 2 at the IRCCS Regina Elena National Cancer Institute (Rome, Italy) were included in this study as part of their standard-of-care clinical management, upon acquisition of written informed consent, between January 2015 and March 2018. Clinical characteristics: median age = 53.5 (30-77), histological type = invasive ductal carcinoma 95%, ductal carcinoma in situ 0%, invasive lobular carcinoma 5%, histological grade at diagnosis = II 30%, II/III 30%, III 35%, unknown 5%, ER status at diagnosis = positive 75%, negative 25%, PR status at diagnosis = positive 65%, negative 35%, HER2 status at diagnosis = positive 80%, negative 20%, Ki-67 status at diagnosis = positive 95%, unknown 5%, number of chemotherapy cycles = 4 5%, 5 5%, 7 5%, 8 70%, 10 5%, unknown 10%, histological grade at surgery = II 20%, III 65%, unknown 15%, ER status at surgery = positive 70%, negative 30%, PR status at surgery = positive 50%, negative 50%, HER2 status at surgery = positive 65%, negative 35%, Ki-67 status at surgery = positive 95%, unknown 5%. Diagnostic and surgical biopsies were studied.
Recruitment	Participants were retrospectivelly included in this study as a part of their standard-of-care management at the IRCCS Regina Elena National Cancer Institute (Rome, Italy). The only criteria fo inclusion were treatment with anthracyclines before surgical resection (neoadjuvant regimen) and sample availabillity.
Ethics oversight	IRCCS Regina Elena National Cancer Institute (Rome, Italy). This study was retrospective as a part of standard-of-care patient management, and hence did not require a dedicated study protocol. This study was conducted in accordance with the Declaration of Helsinki. All the patients signed a written informed consent to treatment and data collection.

Note that full information on the approval of the study protocol must also be provided in the manuscript.

ChIP-seq

Data deposition

☒ Confirm that both raw and final processed data have been deposited in a public database such as GEO.

☒ Confirm that you have deposited or provided access to graph files (e.g. BED files) for the called peaks.

Data access links<br>*May remain private before publication.*	*For "Initial submission" or "Revised version" documents, provide reviewer access links. For your "Final submission" document, provide a link to the deposited data.*
Files in database submission	*Provide a list of all files available in the database submission.*
Genome browser session (e.g. UCSC)	*Provide a link to an anonymized genome browser session for "Initial submission" and "Revised version" documents only, to enable peer review. Write "no longer applicable" for "Final submission" documents.*

Methodology

Replicates	*Describe the experimental replicates, specifying number, type and replicate agreement.*
Sequencing depth	*Describe the sequencing depth for each experiment, providing the total number of reads, uniquely mapped reads, length of reads and whether they were paired- or single-end.*
Antibodies	Rabbit recombinant anti-LSD2/AOF1 (EPR18508) Abcam Cat# AB193080

Peak calling parameters	*Specify the command line program and parameters used for read mapping and peak calling, including the ChIP, control and index files used.*
Data quality	*Describe the methods used to ensure data quality in full detail, including how many peaks are at FDR 5% and above 5-fold enrichment.*
Software	*Describe the software used to collect and analyze the ChIP-seq data. For custom code that has been deposited into a community repository, provide accession details.*

Flow Cytometry

Plots

Confirm that:

☒ The axis labels state the marker and fluorochrome used (e.g. CD4-FITC).

☒ The axis scales are clearly visible. Include numbers along axes only for bottom left plot of group (a 'group' is an analysis of identical markers).

☒ All plots are contour plots with outliers or pseudocolor plots.

☒ A numerical value for number of cells or percentage (with statistics) is provided.

Methodology

Sample preparation

In vitro experiments: to assess the expression of specific surface markers on putative-induced CSCs, 1 x 105 murine and human tumor cells were cultured in 6-well plates in 2 mL of growth medium and treated 72h with purified mouse IFN-α/β or recombinant human Roferon-A® (6000 U/mL) or with DOX (25 µM) or OXP (300 µM) alone or in combination with TCP (10 µM) for 48h. Cells were then collected, washed in Dulbecco's Phosphate-Buffered Saline (D-PBS) and stained with fluorescently labeled mAbs directed against human/murine CD44, CD133 and/or CD24, or with purified-CD44v6 mAb, at optimal mAb concentrations (dilution 1:20, as previously determined by titration), in a cold D-PBS solution containing 1% FBS (D-PBS-FBS 1%). Samples were incubated in the dark on ice for 30min and then washed twice with cold D-PBS-FBS 1% solution. Thereafter, cells stained with CD44v6 mAb, were co-stained with the appropriate Alexa Fluor® 488 secondary Ab (diluted at 1:500 in D-PBS-FBS 1%) on ice for 30min. Cells were washed twice before the addition of 150 µL growth medium supplemented with 1 µg/mL DAPI. For the assessment by flow cytometry of the expression of immune checkpoint molecules, FACS-isolated ICD-CSCs from AT3 and MCA205 cells were stained at 4°C for 30min in the dark with the following murine fluorochrome-conjugated mAbs directed against: PD-L1 (diluted at 1:100); PD-L2CD1LG (diluted at 1:100), LGALS9 (diluted at 1:20) and CEACAM1 (diluted at 1:100). DAPI was used to distinguish live and dead cells, and analysis of the expression of immune checkpoint molecules was made only in live cells. To evaluate how free nucleic acids contribute to the acquisition of CSC traits, 3 x 105 murine tumor cells were cultured in 6-well plates (2 mL of medium/well) and treated with 300 µM OXP for 24h (“donor” cells). Thereafter, “donor” cells were collected, washed from OXP and incubated at 37°C for up to 4h in 1,5 mL-eppendorf microtubes containing growth medium, supplemented or not, with 200 IU/mL BNZase, 10 IU/mL RNase A, 10 IU/mL RNase H or 100 IU/mL DNase. Next, such “donor cells” were cocultured with untreated live cells (“receiving” cells) for 24h in the presence or not of the indicated nucleases before cytofluorometric-mediated assessment of CSC surface markers on “receiving” cells. For the side-population (SP) assay, 1 x 105 murine tumor cells were cultured in 6-well plates (2 mL of medium/well) and treated with 6000 U/mL IFN-α/β for 72h, or 2.5 µM DOX for 48h. Cells were then collected, washed and incubated in pre-warmed growth medium in the presence or not of 100 µM VRP for 30min at 37°C. Five µg/mL Hoescht 33342 was added to cell suspension for 90min at 37°C in the dark. For T cell proliferation and cancer cell killing assays, MCA205-OVA were UV irradiated and co-cultured with BM-derived DCs at a 2:1 ratio for 24h. DCs were then cultured at a 5:1 ratio with splenic purified CD8+ OT-1cells for 72h. Cross-primed CD8+ OT-1 cells were then labelled with 1 µM CFSE dye for 10min at 37°C, and re-stimulated with live parental or CD44L MCA205-OVA cells at 1:5 ratio. Three days later, cells were recovered and analyzed for CFSE levels on live gated CD8+ cells and PI levels on CD45- cells.

Ex vivo experiments: tumors from mice either treated with CDDP, DOX, D-PBS, TCP, DOX+TCP, acute high dose IFNs-I, chronic low dose IFNs-I, chronic low dose IFNs-I+DOX were carefully removed 15 days after treatment. Tumor burdens were cut into small pieces with scissors within digesting buffer (400 U/ml Collagenase A and 200 U/ml DNase I in RPMI 1640) and incubated for 30min at 37°C. Single cell suspensions obtained by grinding the digested tissue and filtering them through a 70-µm cell strainer were then purified based on CD45 expression, by using mouse CD45 MicroBeads, MACS columns and separators (used following manufacter’s recommandations). After washing with D-PBS, CD45+ cells, uncluding tumor infiltrating lymphocytes (TILs), were resuspended at 1 x 107 cells/mL and stained at 4°C for 30min in the dark with the following murine-specific fluorochrome-conjugated mAbs directed against: CD45 (diluted at 1:25); CD8a (diluted at 1:150); and TIM-3 (diluted at 1:100). Similarly, the CD45- cellular fraction (including tumor cells) was stained as follows: CD45, CD133, CD44, CD24 and Nanog (diluted at 1:5). DAPI and Sytox blue were used to distinguish live and dead cells and only live cells were included in the analysis.

Instrument	BD FACSCantoTM II (BD Biosciences), MACSQuant® VYB Analyzer 10 (Miltenyi Biotec), CytoFLEX (Beckman Counter)
Software	FlowJo v.10.0.7 (FlowJo LLC, TreeStar, Inc.)
Cell population abundance	Sorted cells were >90% pure, as determined by FACS reanalysis
Gating strategy	A relevant gating strategy is described in Extended Data 1. Briefly, surface or intracellular markers were quantified within DAPI- cells upon gating on cells (SSC-A vs FSC-A) and singlets (SSC-A vs SSC-H).

☒ Tick this box to confirm that a figure exemplifying the gating strategy is provided in the Supplementary Information.
